# Blood-derived APLP1^+^ extracellular vesicles are potential biomarkers for the early diagnosis of brain diseases

**DOI:** 10.1126/sciadv.ado6894

**Published:** 2025-01-01

**Authors:** Yuri Choi, Jae Hyun Park, Ala Jo, Chul-Woo Lim, Ji-Min Park, Jin Woo Hwang, Kang Soo Lee, Young-Sang Kim, Hakho Lee, Jisook Moon

**Affiliations:** ^1^ Department of Biotechnology, College of Life Science, CHA University, Gyeonggi-do 13488, Republic of Korea.; ^2^ Center for Systems Biology, Massachusetts General Hospital, Harvard Medical School, Boston, MA 02114, USA.; ^3^ Department of Psychiatry, CHA Bundang Medical Center, CHA University College of Medicine, Gyeonggi-do 13496, Republic of Korea.; ^4^ Department of Family Medicine, CHA Bundang Medical Center, CHA University College of Medicine, Gyeonggi-do 13496, Republic of Korea.

## Abstract

The early detection of neurodegenerative diseases necessitates the identification of specific brain-derived biomolecules in peripheral blood. In this context, our investigation delineates the role of amyloid precursor-like protein 1 (APLP1)—a protein predominantly localized in oligodendrocytes and neurons—as a previously unidentified biomarker in extracellular vesicles (EVs). Through rigorous analysis, APLP1^+^ EVs from human sera were unequivocally determined to be of cerebral origin. This assertion was corroborated by distinctive small RNA expression patterns of APLP1^+^ EVs. The miRNAs’ putative targets within these EVs manifested pronounced expression in the brain, fortifying their neurospecific provenance. We subjected our findings to stringent validation using Thy-1 GFP M line mice, transgenic models wherein GFP expression is confined to hippocampal neurons. An amalgamation of these results with an exhaustive data analysis accentuates the potential of APLP1^+^ EVs as cerebrally originated biomarkers. Synthesizing our findings, APLP1^+^ EVs are postulated not merely as diagnostic markers but as seminal entities shaping the future trajectory of neurodegenerative disease diagnostics.

## Teaser

Noninvasive blood detection of brain-derived vesicles enables early and precise diagnosis of brain diseases.

## INTRODUCTION

Neurodegenerative diseases, including Alzheimer’s disease (AD) and Parkinson’s disease (PD), manifest a protracted preclinical phase characterized by silent pathological alterations. This asymptomatic stage poses substantial diagnostic challenges, underscoring the need for reliable biomarkers for early disease detection and progression monitoring (*1*–*3*). Prevailing imaging and cerebrospinal fluid (CSF) biomarkers, unfortunately, often lack the precision needed in clinical settings due to their limited dynamic range (*4*–*6*). Given the invasive nature of CSF-based diagnostics and the high costs associated with imaging modalities, there is a pressing demand for noninvasive, repeatable biomarker detection methods (*7*–*10*). Recent research trends point toward blood-derived extracellular vesicles (EVs) with neural origins as a promising avenue for direct pathogenic assessment in the brain (*11*–*13*). EVs, lipid-bilayer membrane vesicles approximately 30 to 1000 nm in diameter, have the capability to traverse the blood-brain barrier (*14*–*16*). Their ubiquitous circulation and diverse cargo, encompassing DNA, RNA, and proteins (*17*–*19*), make them potential goldmines for biomarker discovery, as documented in datasets like ExoCarta and Vesiclepedia (*20*, *21*). While brain-secreted EVs, potentially rich in brain pathology–related molecules, have piqued research interest (*11*–*13*), their full diagnostic potential remains untapped (*22*, *23*).

L1 cell adhesion molecule (L1CAM) has been used as a biomarker for isolating neuron-derived EVs (NDEVs) (*22*, *23*). However, its purported specificity for neurons has come under scrutiny given findings of its expression in diverse nonneuronal tissues (*24*, *25*). Furthermore, L1CAM’s involvement in various cellular interactions, especially in the context of cancer (*26*–*31*), raises concerns about its precision in distinguishing NDEVs. The overlap between L1CAM’s expression in neuronal and nonneuronal cells casts shadows on its utility as an exclusive marker for brain disease diagnosis (*32*, *33*).

Given L1CAM’s diagnostic limitations in neurodegeneration, our study investigates to identify a robust brain-specific biomarker. We pinpointed amyloid precursor-like protein 1 (APLP1), prevalently expressed in oligodendrocytes and neurons, as a previously unidentified EV biomarker. Rigorous assays corroborated the cerebral origin of APLP1^+^ EVs isolated from human sera, further strengthened by characteristic small RNA brain signatures and specific neural proteins. The robust detection of brain-associated targets within APLP1^+^ EVs underscored their neural specificity. Validation with Thy-1 green fluorescent protein (GFP) M line mice consistently demonstrated GFP and APLP1 coexpression, emphasizing APLP1^+^ EVs’ transformative potential in neurodegenerative diagnostics.

## RESULTS

### Potential of APLP1 as BDEV biomarkers in diagnosing neurodegenerative disorders

**Fig. 1.**
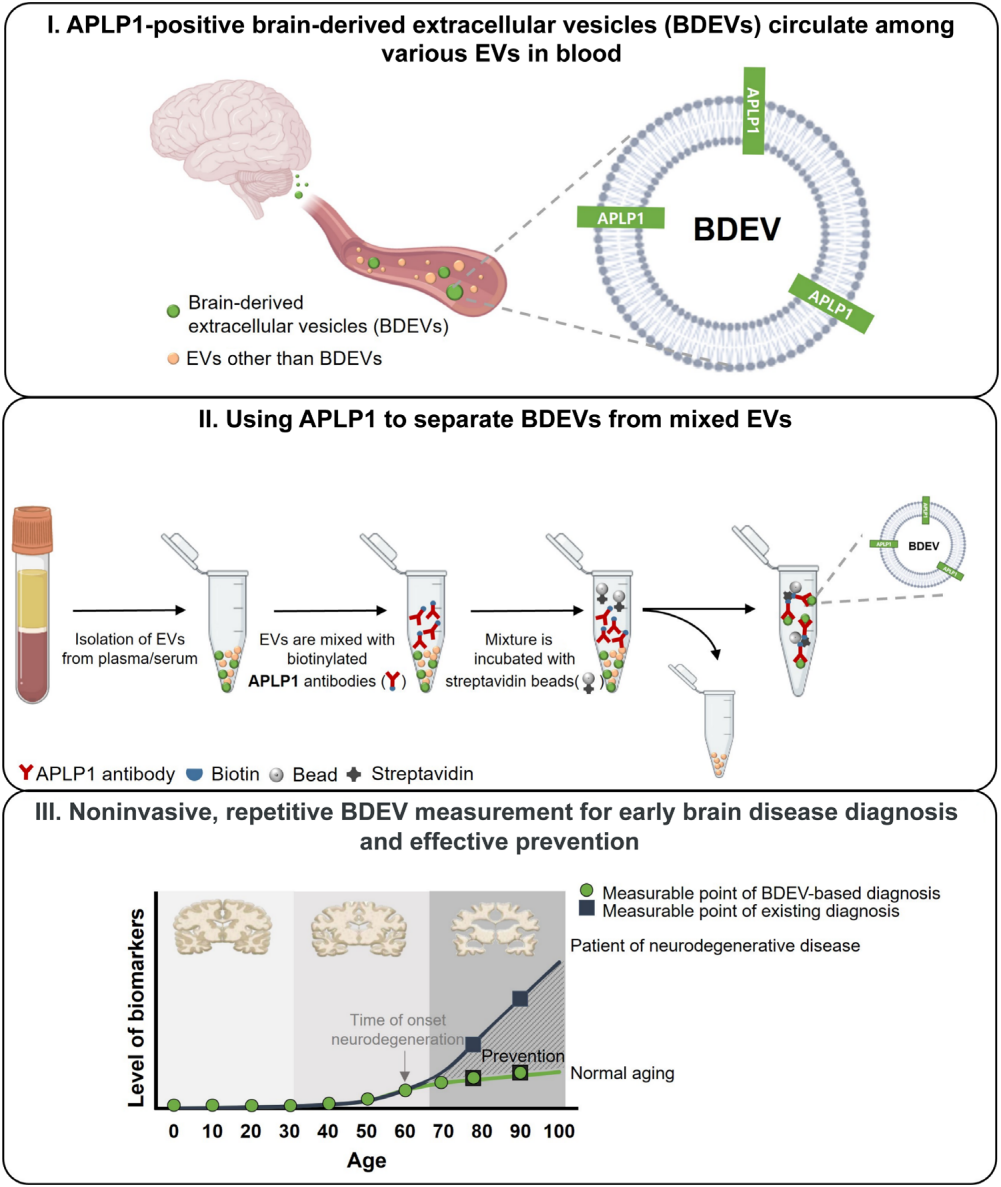
A schematic representation elucidating the diagnostic potential of APLP1^+^ BDEVs in blood for neurodegenerative conditions. The bloodstream contains a mixture of EVs: those derived from the brain (BDEVs, depicted as small green circles) and others originating from various organs (represented as small light orange circles). A distinguishing feature of BDEVs is the presence of the brain-specific protein APLP1 on their membrane (step I). Leveraging an APLP1-specific antibody, it becomes feasible to selectively isolate these BDEVs from the milieu of circulating EVs, filtering out vesicles released from other organs (step II). Periodic assessments leveraging this BDEV-centric approach can facilitate early disease detection, allowing for real-time monitoring of brain health. Such proactive tracking has the potential to preempt or mitigate disease progression, offering another avenue in neurodegenerative disease management (step III).

In Fig. 1, a conceptual diagnostic workflow illustrates the potential of APLP1^+^ brain-derived EVs (BDEVs) in the blood for diagnosing neurodegenerative diseases, presented as an example within a spectrum of brain diseases including brain tumors. EVs, originating from diverse cell types, including brain cells, freely circulate in the bloodstream. Among these, vesicles exhibiting the APLP1 on their surface can be selectively isolated. These APLP1^+^ BDEVs, when captured using immunocapture techniques, show promise as diagnostic tools for brain conditions, including both degenerative diseases and brain tumors (Fig. 1, step I and step II). The process of extracting BDEVs from the bloodstream stands out for its noninvasiveness, offering the advantage of regular monitoring. The molecular cargo within these APLP1^+^ EVs provides an intimate glimpse into the cerebral milieu. This could permit earlier detection of neuropathologies compared to conventional diagnostic methodologies. Such early pinpointing, facilitated by this BDEV-centric methodology, is instrumental in potentially halting the progression of certain brain disorders (Fig. 1, step III). Moreover, the diagnostic paradigm revolving around BDEVs offers distinct benefits: It facilitates early detection, is cost-effective, and requires only a minimal blood sample, contrasting sharply with some current diagnostic methods which might be expensive, invasive, or subjective. Harnessing BDEVs, therefore, promises not only early diagnosis but also timely therapeutic interventions, potentially revolutionizing the management of various brain diseases, especially neurodegenerative diseases as well as brain tumors.

### Strategic integration of diverse datasets to identify brain-specific BDEV biomarkers

**Fig. 2.**
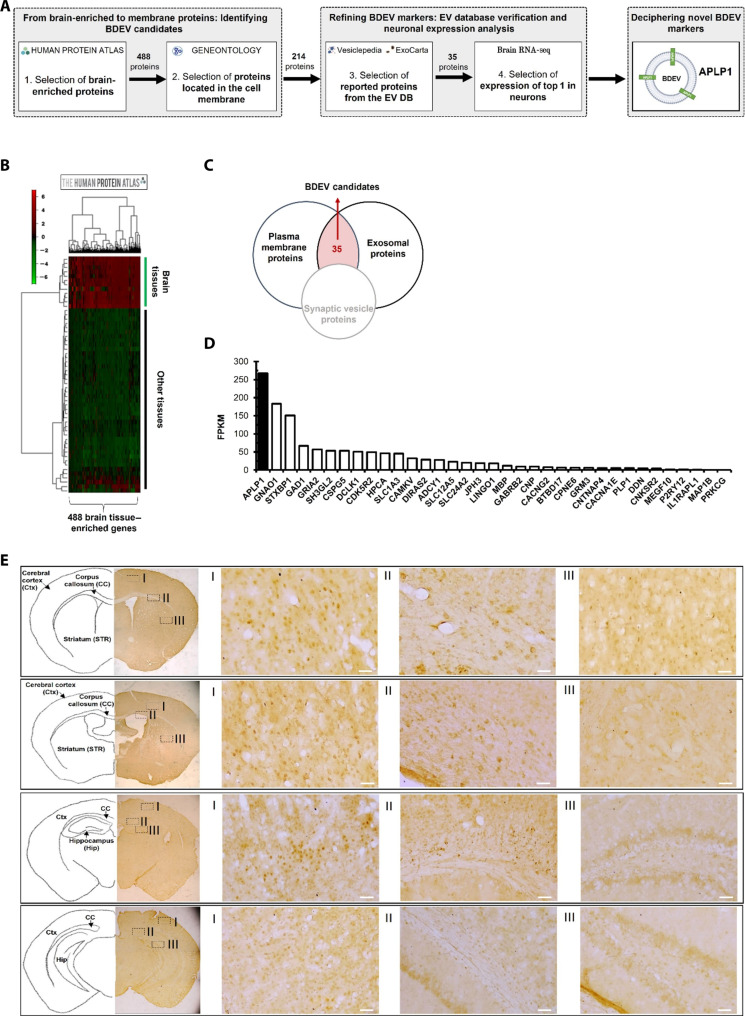
Comprehensive approach for BDEV biomarker identification. (**A**) An in-depth in silico workflow delineating the process for unearthing brain-specific BDEV markers. (**B**) A heatmap detailing the RNA expression for brain-specific proteins across different human tissues, providing a visual representation of tissue-specific expression. (**C**) A Venn diagram illustrating the convergence of our identified brain-specific plasma membrane proteins with entries from the ExoCarta and Vesiclepedia databases, resulting in 35 potential BDEV markers. (**D**) A comparative analysis of the expression magnitudes of the 35 BDEV candidates based on their FPKM (fragments per kilobase per million reads) values in neurons, drawing data from the Brain RNA-seq database. (**E**) Immunohistochemical panels from seven independent assays depict DAB staining localized to APLP1 within diverse cerebral territories. Brain sections derived from seven C57BL/6 mice. The images on the left provide an overarching perspective of the whole brain sections, emphasizing the pervasive presence of APLP1 (×2 magnification). Images on the right (I to III) are enlarged (×200 magnification) highlighting APLP1 distribution across specific brain areas. Additional data can be explored in fig. S1. Scale bars, 50 μm. Ctx, cerebral cortex; Str, striatum; CC, corpus callosum; Hip, hippocampus.

Figure 2 shows schematic representation of our strategic approach for the identification of brain-specific BDEV markers. Through a rigorous integration and analysis of diverse datasets, we aimed to unveil BDEV biomarkers that could enable precise brain disease diagnosis using blood specimens (Fig. 2A). To identify brain-specific BDEV markers, we explored various databases, including the Human Protein Atlas (HPA), Gene Ontology (GO), Vesiclepedia, Exocarta, and brain RNA sequencing (RNA-seq) databases. From the tissue-specific proteome in the HPA database, we initially identified 488 proteins with exclusive expression in the brain. The expression levels of these 488 proteins were then visualized using a heatmap. (Fig. 2B and table S1). Given that EVs originate either by the direct budding off from the cellular plasma membrane or from the endosomal membrane’s inward invagination, culminating in the fusion of the multivesicular body with the cellular plasma membrane (*19*, *34*, *35*), it became pivotal to pinpoint proteins integral to the EV membrane to ascertain their parent cells. This led us to focus on proteins situated on the cell plasma membrane out of the initial 488 brain-specific proteins. For this analysis, we performed GO annotation, specifically applying the cellular component term “GO:0005886 plasma membrane” to identify proteins located on the plasma membrane of brain cells. To ensure the focus on EV-related proteins, we excluded proteins associated with the term “GO:0008021 synaptic vesicle” to avoid including membrane proteins found in vesicles other than EVs. Consequently, 214 proteins were shortlisted, eliminating vesicles encapsulating neurotransmitters among EVs that had brain-specific proteins on the plasma membrane. To further refine our list to proteins that are present within EVs, we cross-referenced our selected proteins with those catalogued in the ExoCarta and Vesiclepedia databases (Fig. 2C and table S2). This strategy culminated in the selection of 35 proteins from the initial 214, which were exclusive to the brain, membrane bound, and documented within EVs. Next, to identify biomarkers that are easy to detect, we examined the expression levels of 35 proteins in the brain. Notably, APLP1 was prominent due to its pronounced expression in neurons and cells that commonly was affected in various types of brain diseases, positioning it as a leading candidate for a brain-specific EV marker (Fig. 2D). This selection rationale is grounded in the understanding that proteins ubiquitously expressed throughout the brain can most aptly elucidate the universal neural alterations and pathologies characteristic of brain disorders (*36*–*38*). To empirically validate our in silico deduction regarding APLP1’s widespread distribution in brain tissue, we assessed APLP1’s protein distribution in mouse brains via 3,3′-diaminobenzidine (DAB) staining. When we examined the protein expression of APLP1 in various brain regions, including the cerebral cortex, corpus callosum, striatum, and hippocampus, APLP1 expression was observed in all regions. These results demonstrate that APLP1 is widely expressed throughout the brain (Fig. 2E and fig. S1).

### Brain-specific expression of APLP1 relative to L1CAM

**Fig. 3.**
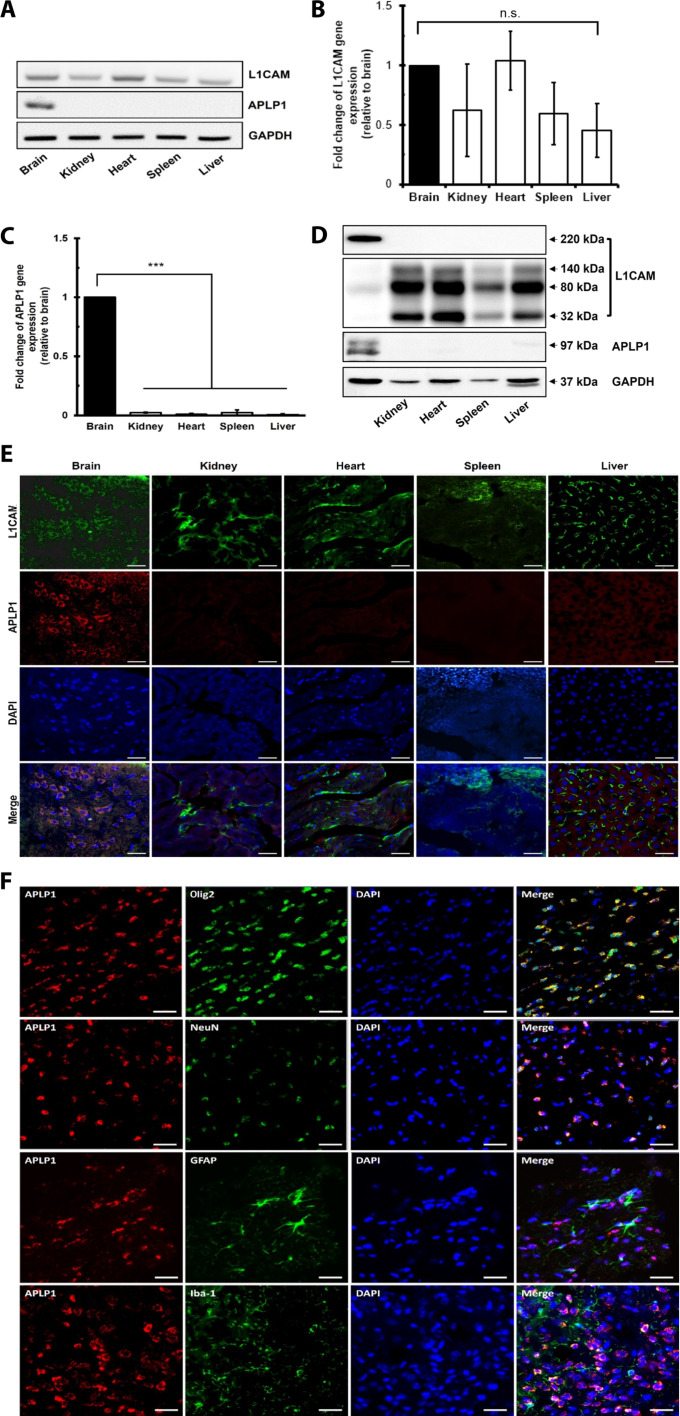
Brain-specific expression of APLP1. (**A**) mRNA profiles of *APLP1* and *L1CAM* across different mouse organs. (**B** and **C**) Relative mRNA expression levels quantified via reverse transcription qPCR, normalized to glyceraldehyde phosphate dehydrogenase (*GAPDH*). Data are presented as the means ± SEM of eight independent experiments, and statistical analysis was performed on 2^Δ*C*t^ values using the analysis of variance (ANOVA; post hoc: Tukey). Symbol “***” indicates a value of < 0.001, respectively, compared with the brain. (**D** and **E**) Western blot representation (D) and immunofluorescence imaging (E) of L1CAM and APLP1 across varied tissues derived from five C57BL/6 mice. (**F**) Use of RNAscope and IHC to detect APLP1 colocalization in mouse cerebral cortex cells. The mRNA of APLP1 is visualized in red, while oligodendrocytes (Olig2), neurons (NeuN), astrocytes (GFAP), or microglial cells (Iba-1) are visualized in green fluorescence. Scale bars, 50 μm. The experiment was performed with three C57BL/6 mice. n.s., not significant; DAPI, 4′,6-diamidino-2-phenylindole.

To elucidate the distribution of APLP1 across tissues, we extracted RNA and protein from diverse mouse organs: the brain, kidney, heart, spleen, and liver. Our comparative evaluation indicated that L1CAM manifested across multiple tissues, whereas APLP1’s expression was primarily confined to the brain (Fig. 3A). Reverse transcription quantitative polymerase chain reaction (qPCR) analysis further elucidated that *L1CAM* was uniformly distributed across the brain and other organs (Fig. 3B). In contrast, *APLP1* showed a pronounced brain-specific expression (Fig. 3C; *P* < 0.001). Analysis of mRNA expression confirmed that *APLP1* is exclusively expressed in the brain (Fig. 3, A to C). Western blotting and immunohistochemistry (IHC) confirmed these findings (Fig. 3, D and E). Notably, while the full-length 220-kDa L1CAM was present in the brain, its cleaved forms (140, 80, and 32 kDa) were more abundant in nonneuronal tissues (Fig. 3D). Our experimental findings align with the differential proteolytic pathways of L1CAM and APLP1. L1CAM is subject to a complex cleavage process, influenced by α-, β-, and γ-secretases. In contrast, APLP1’s cleavage is predominantly driven by γ-secretase, as highlighted by a recent scientific study (*39*). Specifically, while the brain exhibited the full-length 220-kDa L1CAM, its cleaved isoforms (140, 80, and 32 kDa) were more prevalent in nonneuronal tissues. Our results indicated a predominant presence of the full-length APLP1 in the examined samples. These distinctions underscore the potential of APLP1^+^ EVs as more reliable and brain-specific biomarkers compared to L1CAM. Immunofluorescence staining further corroborated these findings, with L1CAM detected in multiple organs, whereas APLP1 was exclusive to the brain (Fig. 3E).

Notably, APLP1 expression was also detected in astrocytes [glial fibrillary acidic protein (GFAP)^+^ cells] and microglia [ionized calcium-binding adapter molecule 1 (Iba-1)^+^ cells]. Among all the cell types analyzed, oligodendrocytes [oligodendrocyte transcription factor 2 (Olig2)^+^ cells] demonstrated the most pronounced APLP1 expression. Moreover, neurons [neuronal specific nuclear protein (NeuN)^+^ cells] also exhibited dominant expression. (Fig. 3F). These results align with recent data from bulk RNA-seq (sourced from brainrnaseq.org) and single-cell RNA-seq (portal.brain-map.org), indicating that APLP1 is predominantly expressed in oligodendrocytes and neurons in both mouse and human brains (fig. S2, C and D). To delve deeper, we first integrated data from the HPA to investigate tissue-specific expression levels of APLP1 (fig. S2, A and B). Our findings confirmed that both the RNA and protein of APLP1 are predominantly found in brain tissues. Building on this, we explored APLP1 expression in individual brain cells. We used databases that highlighted cell-specific expression through immunocapture in human or mouse brain cells and also drew from databases focused on human brain single-nuclei sequencing (http://adsn.ddnetbio.com) (fig. S2E). Our in-depth analysis revealed that APLP1 expression was most pronounced in oligodendrocytes among brain cells, followed closely by neurons. In a broader context, our collective data underscore the strong brain specificity of APLP1, especially when juxtaposed with L1CAM.

### APLP1 expression in EVs from mouse and human plasma

**Fig. 4.**
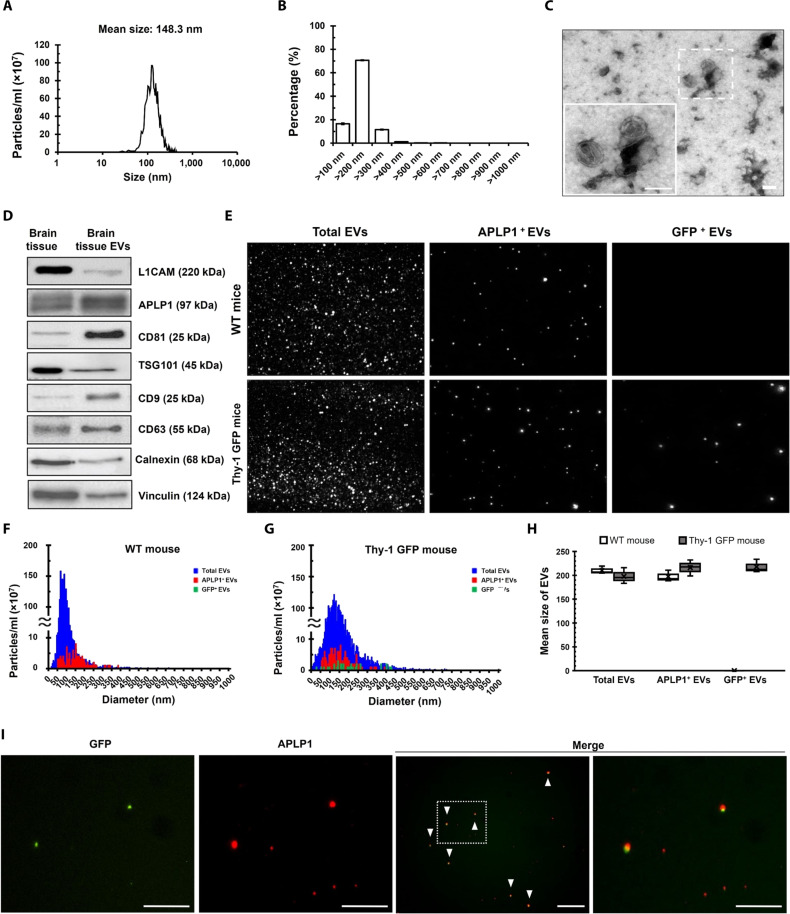
APLP1^+^ EVs originate from the brain. (**A**) NTA-derived graphs showing size and concentration metrics of the brain tissue EVs. (**B**) Percentage-based size distributions of EVs, derived from the data presented in (A). (**C**) TEM images, at ×10,000 and ×20,000 magnifications, depicting EV morphology. Scale bars, 100 nm. Error bars in the figures delineate the SE of the mean (±SEM) from triplicate measurements of brain tissue EVs. (**D**) Western blot of APLP1 and EV markers in brain tissues and brain tissue EVs from five C57BL/6 mice. (**E**) ZetaView analysis of plasma-derived EVs in WT and Thy-1 GFP M line mice. Total EVs appear in light-scatter mode, GFP^+^ EVs in 488-nm fluorescence mode, and APLP1^+^ EVs in 640-nm fluorescence mode. (**F** and **G**) EV size distribution in WT and Thy-1 GFP M line mice using ZetaView. (**H**) Average EV size ranges between 198 and 217.8 nm in both mouse types. (**I**) Immunostaining of APLP1 in plasma EVs from Thy-1 GFP M line mice, with white arrowheads highlighting APLP1^+^GFP^+^ EVs. Scale bars, 25 μm. All data were obtained from at least three mice.

**Fig. 5.**
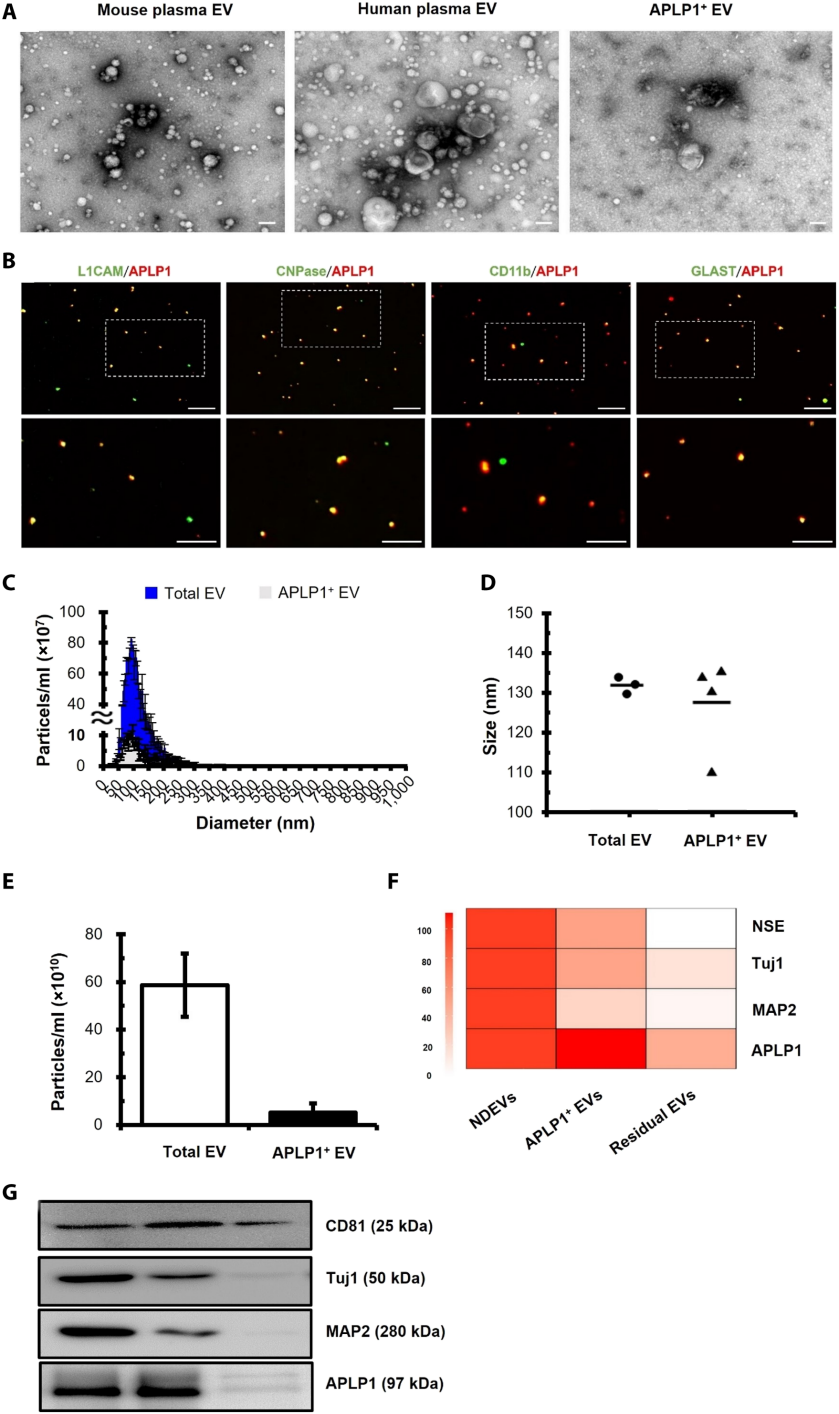
Comprehensive characterization of APLP1-enriched EVs from human plasma. (**A**) TEM images showing the morphology of mouse plasma EVs, human plasma EVs, and APLP1^+^ EVs. Scale bars, 100 nm. (**B**) Representative florescence staining images. Overlay of APLP1-stained EVs (in red) and brain cell markers (in green) from human plasma. Scale bars, 10 μm. (**C**) Size distribution patterns of various EV samples determined via NTA. (**D**) Modal size comparisons of EV populations. (**E**) Concentrations of EVs, highlighting the relative abundance of APLP1^+^ EVs in the overall plasma EV pool. Data are presented as means ± SEM. (**F**) A comparative analysis of mRNA expression levels for neuronal markers in human neuronal cell-derived EVs (hNDEVs), APLP1^+^ EVs, and the residual EVs. (**G**) Western blot showing the neuronal markers in APLP1^+^ EVs compared with those in residual EVs from human plasma. Results were based on at least three separate experiments. NSE, neuron-specific enolase.

To further confirm the specific expression of APLP1 in BDEVs, we isolated EVs from mouse brain tissues and extracted proteins from these brain tissue–derived EVs. We then characterized the isolated EVs using nanoparticle tracking analysis (NTA) and transmission electron microscopy (TEM) to determine their size, concentration, and morphology (Fig. 4, A to C). NTA using ZetaView showed that the diameter of these vesicles was mainly distributed between 50 and 400 nm, with the mean diameter of the isolated brain tissue EVs measured at 143.1 nm (Fig. 4A). Notably, EVs sized between 100 and 200 nm were the most abundant (Fig. 4B). Furthermore, the isolated brain tissue EVs exhibited typical EV morphology (Fig. 4C). To ascertain the extent of APLP1 expression in the brain, we tested the protein levels in both brain tissue and BDEVs (Fig. 4D). The analysis highlighted a marked presence of EV markers including CD9, CD63, CD81, and TSG101 in the BDEVs, surpassing the expression levels in the brain tissue. Despite the substantial findings, it is important to note a slight detection of calnexin, an endoplasmic reticulum marker, suggesting a minor contamination of the BDEVs with vesicles originating from the endoplasmic reticulum. For the Western blot analysis, vinculin served as a reliable loading control. A noteworthy observation from the comparative analysis of expression levels was the distinct patterns exhibited by L1CAM and APLP1 in both the brain tissue and BDEVs. While L1CAM was more abundantly expressed in the brain tissue as opposed to the BDEVs, APLP1 manifested a stronger expression within the BDEVs, contrasting its expression levels in the brain tissue. This differential expression underscores a marked variation in the expression profiles of these entities. To further validate our findings concerning the neural origin of APLP1^+^ EVs, we used Thy-1 GFP M line mice for additional experimentation. These transgenic mice are engineered to have enhanced GFP (EGFP) expression hippocampal neurons under the control of a modified Thy-1 promoter region that contains the sequences required for neuronal expression (*40*, *41*). As delineated in fig. S3, a substantial presence of labeled cells was observed within the hippocampal region, while their occurrence in the cortex was relatively sparse (fig. S3). When juxtaposing the expression levels of GFP and APLP1 in plasma EVs of wild-type (WT) and Thy-1 GFP M line mice, several insightful observations were made. First, NTA of plasma EVs from both mouse types revealed consistent concentrations of total EVs under light-scatter mode (Fig. 4E and fig. S4, A and D), underscoring the uniformity in EV isolation across both mouse strains. Using fluorescence mode, both APLP1^+^ EVs (640 nm) and GFP^+^ EVs (488 nm) were characterized. APLP1^+^ EVs were discernible in both WT and Thy-1 GFP M line mice, exhibiting analogous concentrations between the two (Fig. 4E and fig. S4, B and E). Conversely, GFP^+^ EVs marked their presence exclusively in plasma EVs from Thy-1 GFP M line mice, absent in the WT counterpart (Fig. 4E and fig. S4, C and F). The EV size distribution remained consistent across both mouse strains (Fig. 4, F and G), showing an average particle dimension ranging between 198 and 217.8 nm (Fig. 4H). Further investigation into the colocalization of GFP and APLP1 in plasma EVs by fluorescence staining validated that APLP1 was present within GFP^+^ EVs (Fig. 4I and fig. S5). Given the constraints of Thy-1 GFP M line mouse’s expression predominantly in the hippocampus and the prevailing expression of APLP1 in oligodendrocytes and neurons, this coexpression not only elucidates the origin and behavior of APLP1^+^ EVs in the circulatory system but also underscores the potential of using APLP1 as a reliable biomarker for brain-specific events, especially given its pronounced affinity for oligodendrocytes and neurons. To more conclusively confirm that APLP1^+^ EVs in the blood are of brain cell origin, including neurons, we isolated EVs from human plasma and investigated the colocalization of APLP1^+^ EV with well-known brain origin markers, such as 2’,3’-cyclic nucleotide 3’-phosphodiesterase (CNPase), L1CAM, CD11b, and GLAST. These findings indicate that APLP1 can be identified not only in L1CAM^+^ EVs but also in CNPase^+^ EVs (anticipated to be derived from oligodendrocytes), GLAST^+^ EVs (presumed to be derived from astrocytes), and CD11b^+^ EVs derived from microglia-derived (Fig. 5B). This coexpression pattern provides compelling evidence underscoring the predominant brain origin of APLP1^+^ EVs.

Subsequently, we systematically verified the feasibility of isolating BDEVs from human plasma samples by using APLP1 as a biomarker, thereby validating the observations noted in murine experiments. First, APLP1^+^ EVs were selectively separated from the human plasma EV pool using a biotinylated APLP1 antibody in conjunction with streptavidin-agarose resin. Using TEM, we examined the morphology of APLP1^+^ EVs isolated from human plasma and found that their appearance was identical to typical human plasma EVs (Fig. 5A).

Subsequent characterization of these APLP1^+^ EVs via NTA revealed a size distribution mirroring that of the total plasma EVs (Fig. 5C). Notably, the modal size remained consistent between the APLP1^+^ EVs and the total EVs (Fig. 5D). However, a disparity in concentration became evident: while the plasma yielded a total of 5.2 × 10^11^ EVs/ml and that of APLP1^+^ EVs was 5.2 × 10^10^ EVs/ml, indicating that APLP1^+^ EVs constituting approximately 10% of the overall EV population (Fig. 5E). To validate the brain-specific nature of EVs isolated using the APLP1 antibody, we embarked on a comprehensive analysis using PCR, Western blotting, and proteomics to scrutinize the expression of brain-related markers within APLP1^+^ EVs. Serving as a reference for the expression of these markers, we used human neuronal cell-derived EVs (hNDEVs). These hNDEVs were isolated from fetal brain tissue at 8 weeks of gestation, a primary neuronal progenitors established in our laboratory (*42*). The expression level of APLP1 in APLP1^+^ EVs paralleled that in hNDEVs (Fig. 5, F and G). Furthermore, a suite of neuron-specific markers, including neuron-specific enolase, microtubule-associated protein 2 (MAP2), and Tuj1 (neuron-specific class III β-tubulin), manifested pronounced expression in APLP1^+^ EVs, especially when juxtaposed with their residual counterparts (Fig. 5, F and G).

### The analysis of cargoes of APLP1^+^ EVs from human plasma

**Fig. 6.**
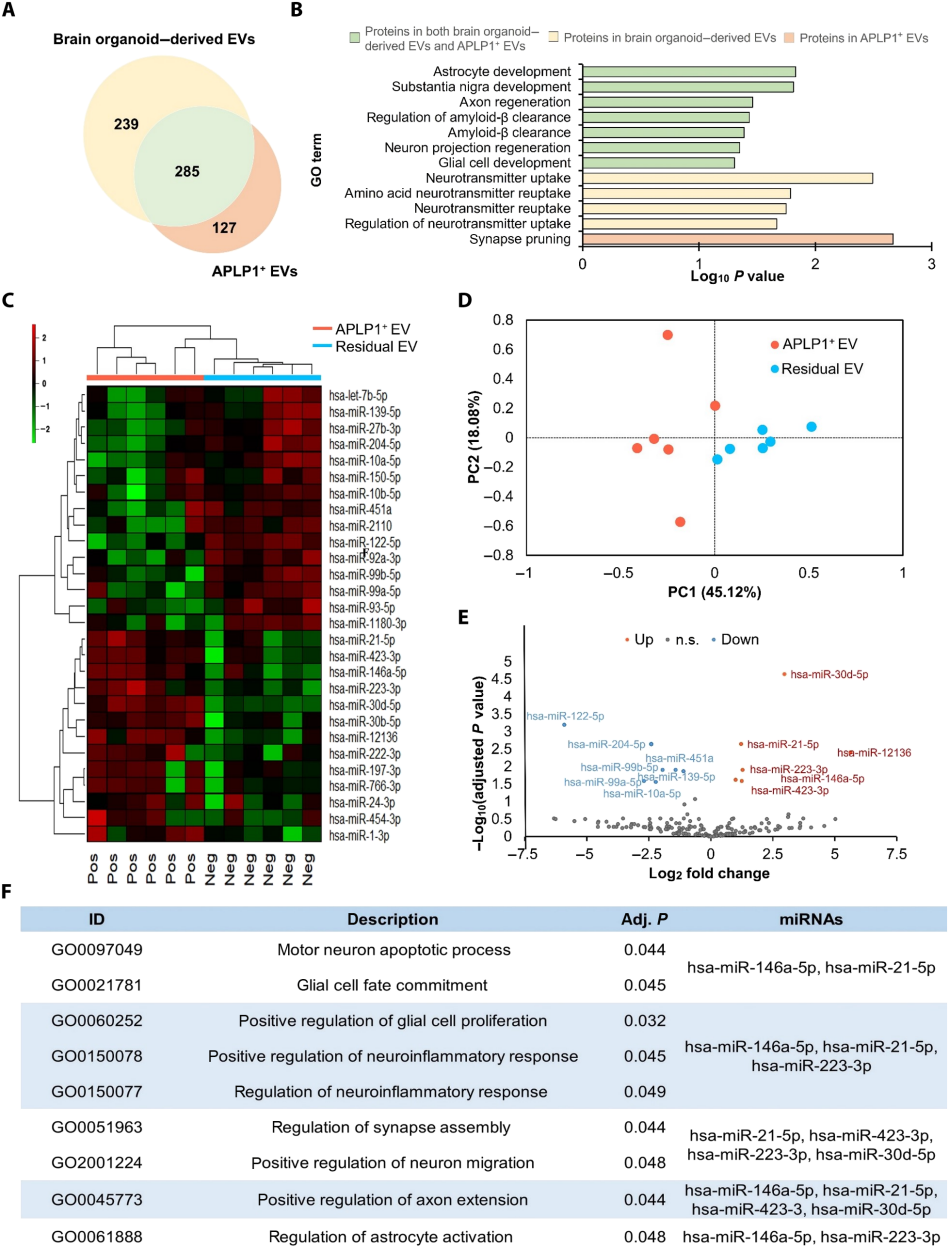
Comprehensive analysis of protein and miRNA profiles in APLP1^+^ EVs and their neurological implications. (**A**) Venn diagram showing the overlap of proteins identified in both brain organoid–derived EVs and APLP1^+^ EVs. (**B**) Bar chart delineating the GO term analysis of proteins detected in both the brain organoid–derived EVs and APLP1^+^ EVs, with a specific emphasis on brain-associated GO terms. The *y* axis enumerates distinct GO terms, while the *x* axis indicates the −log_10_ adjusted *P* value. This visualization offers insights into brain-functional annotations derived from the GO enrichment analysis. (**C**) Heatmap illustrating the differential expression patterns of miRNAs between APLP1^+^ EVs and residual EVs. Within the heatmap, rows have been centered, and unit variance scaling is applied to the normalized expression data. Shades of red denote elevated expression levels, whereas green shades indicate reduced expression levels. The Pos indicate APLP^+^ EVs and the Neg indicate Residual EVs. (**D**) Principal components analysis (PCA) visualizing the variance in miRNA profiles between APLP1^+^ EVs and residual EVs. (**E**) Volcano plot that elucidates the differential miRNA expression between APLP1^+^ EVs and residual EVs. The graph’s orientation allows for easy identification of miRNAs that are either more abundant in APLP1^+^ EVs (points to the right) or residual EVs (points to the left). The *y* axis offers a perspective on the −log_10_ adjusted *P* value, providing a quick gauge of significance. (**F**) A GO term analysis on the targets of up-regulated miRNAs in APLP1^+^ EVs, leveraging the miEAA tool. Brain-associated terms are highlighted, with nine terms significantly enriched in APLP1^+^ EVs, in stark contrast to the absence of such terms in residual EVs. The EVs used in the analysis were obtained from plasma of six volunteers.

We further conducted proteomics to identify the protein cargo present in APLP1^+^ EVs. Moreover, to ascertain the similarity between the APLP1^+^ EVs and BDEVs, we used the EVs isolated from the culture medium of brain organoids created using our Neural Progenitor Cells (NPCs) (*42*) as a comparative control (Fig. 6). Consequently, we identified 239 proteins exclusive to the brain organoid–derived EVs, which served as the positive control for BDEVs, and 127 proteins that were exclusively found in the APLP1^+^ EVs. Furthermore, a total of 285 proteins were detected in both groups (Fig. 6A). To explore the functionalities of the identified proteins, we carried out a GO term analysis (table S3), presenting the brain-related GO terms in Fig. 6B. Proteins that were commonly found in both brain organoid–derived EVs and APLP1^+^ EVs were enriched in various brain-related terms such as “astrocyte development” (adjusted *P* value < 0.01), “substantia nigra development” (adjusted *P* value < 0.01), “axon regeneration” (adjusted *P* value < 0.03), “regulation of amyloid-β clearance” (adjusted *P* value < 0.03), “amyloid-β clearance” (adjusted *P* value < 0.04), “neuron projection regeneration” (adjusted *P* value < 0.04), and “glial cell development” (adjusted *P* value < 0.04). Proteins exclusively detected in the brain organoid–derived EVs were primarily enriched in neurotransmitter-related brain functions such as “neurotransmitter uptake” (adjusted *P* value < 0.003), “amino acid neurotransmitter reuptake” (adjusted *P* value < 0.01), “neurotransmitter reuptake” (adjusted *P* value < 0.01), and “regulation of neurotransmitter uptake” (adjusted *P* value < 0.02). Moreover, proteins found only in APLP1^+^ EVs showed an enrichment in “synapse pruning” (adjusted *P* value < 0.002). These findings can be speculated to represent a transitional phase toward understanding the role of EVs at the mature stage, given that the study used the early development stage of the brain organoid. Through the proteomic analysis, we verified that approximately 69% of the proteins found in the APLP1^+^ EVs were also present in the brain organoid–derived EVs, exhibiting brain-related functionalities further demonstrating that APLP1^+^ EVs represent the brain, as evidenced by the substantial overlap in protein cargo with brain organoid–derived EVs, showing a diversity of brain-related functionalities and enrichment in critical neurological processes.

In our comprehensive exploration of APLP1^+^ EVs, we extended our analysis beyond mRNAs and proteins to investigate microRNAs (miRNAs) and ascertain whether their levels provide insights into brain conditions. Small RNA-seq revealed distinct miRNA profiles between APLP1^+^ EVs and residual EVs. It is important to note that while residual EVs represent all EVs not enriched in APLP1^+^ and may very likely contain APLP1^+^ EVs, they still showed a clear difference in their miRNA expression patterns (Fig. 6C). This observation aligns with our previous proteomics and Western blotting results. Further validation using principal components analysis (PCA) distinctly separated the APLP1^+^ EVs from the residual EVs (Fig. 6D). Delving deeper into the tissue specificity of miRNAs within APLP1^+^ EVs, we identified 180 differentially expressed miRNAs in comparison to residual EVs. Of these, 13 miRNAs showed significant differential expression (*ps* < 0.05), with 6 miRNAs being up-regulated (hsa-miR-30d-5p, hsa-miR-21-5p, hsa-miR-12136, hsa-miR-223-3p, hsa-miR-146a-5p, and hsa-miR-423-3p) and 7 being down-regulated (hsa-miR-122-5p, hsa-miR-204-5p, hsa-miR-451a, hsa-miR-99b-5p, hsa-miR-139-5p, hsa-miR-99a-5p, and hsa-miR-10a-5p) (Fig. 6E). To ascertain whether the six up-regulated miRNAs in APLP1^+^ EVs are related to the brain, we analyzed the target genes of these miRNAs and conducted a GO term analysis based on the miRNA enrichment analysis and annotation (miEAA) tools. As depicted in Fig. 6F, five of the miRNAs, excluding hsa-miR-12136, were predicted to be associated with brain functions. Specifically, hsa-miR-21-5p was associated with eight distinct brain-related GO terms, while hsa-miR-146a-5p had associations with seven, and hsa-miR-223-3p with six. Both hsa-miR-423-3p and hsa-miR-30d-5p had associations with three brain-related GO terms each. Moreover, hsa-miR-21-5p, hsa-miR-146a-5p, and hsa-miR-223-3p have been reported to play roles in brain functions, consistent with our GO term analysis predictions. hsa-miR-21-5p is present in neuron-derived exosomes and is implicated in the polarization of M1 microglia (*43*, *44*), while hsa-miR-146a-5p is reported to modulate neurogenesis via microglial-derived exosomes in states of depression (*45*). hsa-miR-223-3p has been documented to alleviate trigeminal neuropathic pain in male mice by targeting MAPK Interacting Serine/Threonine Kinase 2 (MKNK2) and the mitogen-activated protein kinase/extracellular signal–regulated kinase signaling pathway (*46*). Combining these insights, we propose that APLP1^+^ EVs are indicative of BDEVs.

### Validation of diagnostic potential of APLP1^+^ EVs for brain diseases

**Fig. 7.**
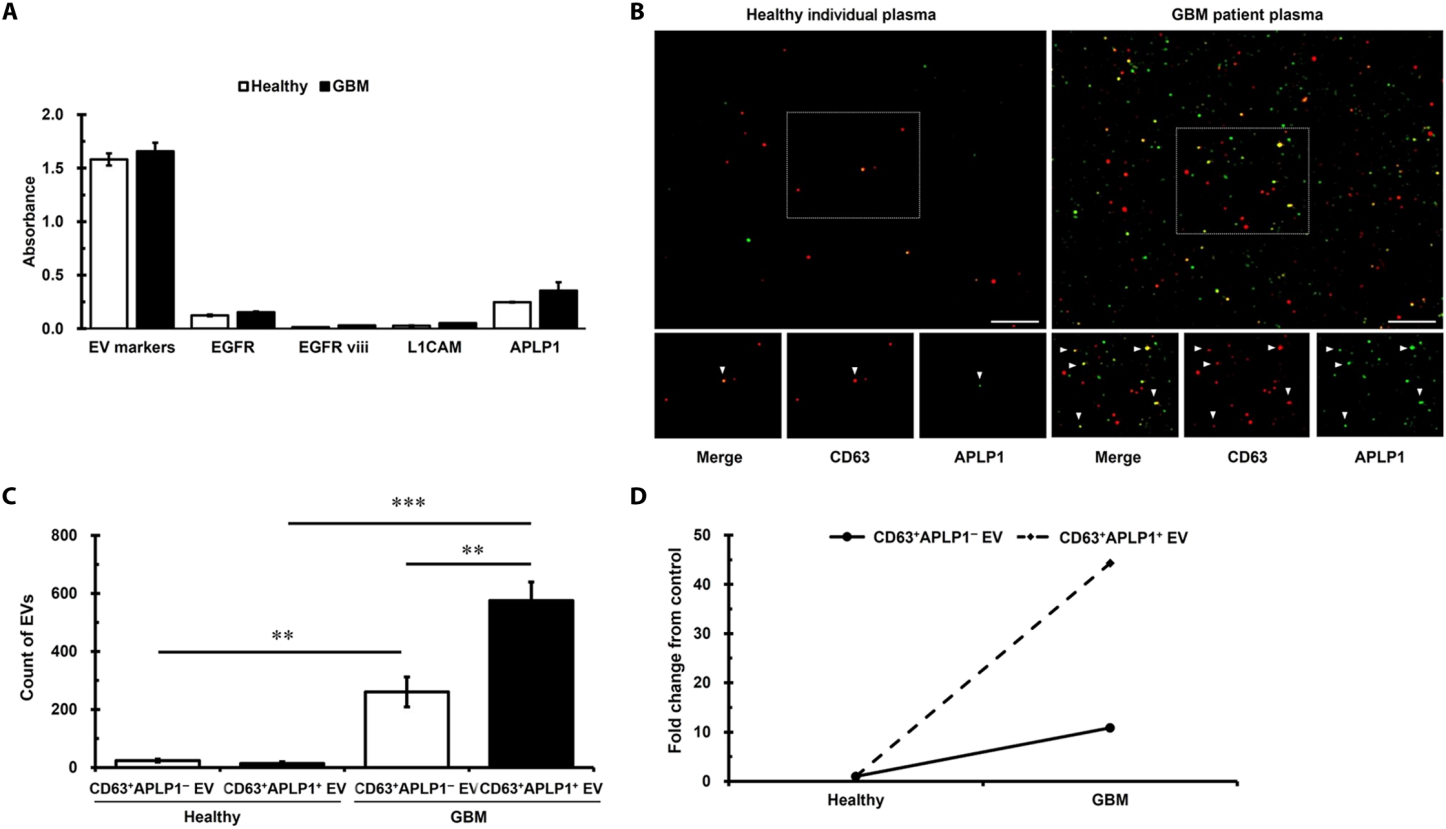
Diagnostic potential of APLP1^+^ EVs in neurological disorders. (**A**) Enzyme-linked immunosorbent assay (ELISA)–derived absorbance units representing the levels of EV markers (CD9, CD81, and CD63) and key antigens (EGFR, EGFRviii, L1CAM, and APLP1) within plasma EVs from the healthy group and the GBM patient group. Data represent means ± SEM of three independent experiments. (**B**) Representative immunostaining images contrasting plasma EVs from healthy individuals and patients with GBM (*n* = 3). The white arrowheads indicates CD63^+^APLP1^+^ EVs. Scale bars, 5 μm. (**C**) Quantification of CD63^+^APLP1^−^ EVs and CD63^+^APLP1^+^ EVs in each group (*n* = 3). (**D**) Fold change ratio of CD63^+^APLP1^−^ EVs and CD63^+^APLP1^+^ EVs between the healthy and GBM groups (*n* = 3). Data represent means ± SEM of three independent experiments. Statistical analysis was conducted using the ANOVA test (post hoc: Tukey). **P < 0.01 and ***P < 0.001, statistical differences.

To further assess the diagnostic potential of APLP1^+^ EVs for brain diseases, we evaluated their expression in the blood of patients with glioblastoma multiforme (GBM) exhibiting EGFR and EGFRviii expression (Fig. 7A). It is well-known that gliomas, including GBM, have origins in glial cells like astrocytes and oligodendrocytes, as well as in neural stem cells found within the brain (*47*, *48*). Their gene expression profiles have been shown to bear resemblance to those of glial cells (*49*–*51*). Notably, glioma-derived EVs are characterized by elevated expression levels of GBM-specific markers, EGFR and EGFRviii (*52*, *53*), suggesting that these EGFR and EGFRviii^+^ EVs are not only derived from gliomas but are also likely to originate from the brain. In the context of emerging evidence underscoring the diagnostic potential of EVs, we embarked on an analysis of biomarker expression within plasma EVs isolated from patients with GBM. Specifically, we quantified the presence of pivotal molecular markers: EGFR, EGFRviii, L1CAM, and APLP1. Of these analytes, APLP1 demonstrated the most elevated expression, substantially surpassing the relative abundances of L1CAM, EGFRviii, and EGFR (Fig. 7A). Significant increases in both the CD63^+^APLP1^−^ EV and CD63^+^APLP1^+^ EV populations were observed in the GBM group (Fig. 7, B and C). In addition, the proportion of CD63^+^APLP1^+^ EVs among the total EV population was calculated to be 36.28% in the healthy group, whereas in the GBM group, it was 68.82% (Fig. 7C).

Quantitatively, there was a 10.83-fold enrichment in the CD63^+^APLP1^−^ EV subset and a staggering 44.3-fold amplification in the CD63^+^APLP1^+^ EV subset, when benchmarked compared to healthy individuals (Fig. 7D). These findings align with reports suggesting that hypoxic conditions, induced in cancer environments including GBM, lead to an increase in the release of EVs (*54*, *55*). Moreover, our results demonstrate a greater increase in the number of CD63^+^ APLP1^+^ EVs compared to CD63^+^ APLP1^−^ EVs in the GBM group. This observation implies that, in response to changes in the brain environment, there is an increased proportion of BDEVs among the overall increase in EVs. Collectively, these data not only corroborate an amplified prevalence of EVs in the GBM milieu but also underscore a distinctive surge in BDEVs. Given these marked perturbations in BDEV abundance in tandem with neurological aberrations, it becomes unequivocally evident that BDEV quantification could serve as an invaluable diagnostic modality in the realm of neuro-oncological disorders.

## DISCUSSION

Numerous investigations have substantiated the utility of BDEVs isolated from blood as diagnostic tools for neurological disorders (*11*–*13*). The ability of BDEVs to traverse the blood-brain barrier renders them detectable in the bloodstream (*56*). Capitalizing on the inherent attributes of EVs, leveraging BDEVs for brain disease diagnosis offers a noninvasive and cost-effective alternative to traditional imaging or CSF modalities. A salient feature of BDEVs is their amenability to recurrent measurements, facilitating timely diagnosis and proactive disease intervention (*11*–*13*). The molecular constituents of BDEVs act as barometers, reflecting the physiological and pathological nuances of brain cells and tissues. This is especially pivotal for the early diagnosis of degenerative brain conditions, which typically present diagnostic challenges (*56*–*58*). Evidently, the facile and safe isolation of BDEVs from blood, coupled with their potential to illuminate the brain’s pathological milieu, paves the way for enhanced early diagnostic strategies, potentially curtailing the progression of neurological ailments.

L1CAM, recognized as a neuron-specific marker, has been extensively used for the isolation of NDEVs (*22*, *23*). However, findings by Angiolini *et al*. (*24*) reveal that L1CAM exhibits expression in nonneuronal cells in tandem with neuronal cells. Our finding corroborates this observation, detecting L1CAM expression in a range of organs including the heart, spleen, kidneys, and liver, alongside the brain (Fig. 3). Furthermore, complicating its role as an exclusive BDEV marker, the L1CAM ectodomain is subject to degradation by a suite of enzymes, including ADAM metallopeptidase domain 10 (ADAM 10), ADAM metallopeptidase domain 17 (ADAM 17), and matrix metalloprotease 16. Consequently, a predominant fraction (approximately 90%) of L1CAM exists as a free protein, detached from EVs, undermining its utility as a standalone biomarker (*27*, *59*–*61*). In agreement with these findings, our study discerned that the majority of L1CAM in the blood was predominantly in its free form rather than being EV associated (Fig. 4D). In addition, the expression of L1CAM was markedly subdued in EVs derived from brain tissue in comparison to its expression in the brain tissue itself.

Both APLP1 and L1CAM play pivotal roles within the neural framework, guiding diverse neural activities. However, their proteolytic processes and ensuing cellular outcomes distinctly diverge. L1CAM is subject to an intricate cleavage matrix, influenced by α-, β-, and γ-secretases. Conversely, APLP1 undergoes a more defined cleavage mechanism, predominantly driven by γ-secretase, a fact substantiated by recent scientific literature (*39*). This particular cleavage births the intracellular domain (ICD) of APLP1. This fragment, known as APLP1-ICD, is believed to relocate to the nucleus, potentially influencing gene expression. Although the functional implications of APLP1’s ICD are acknowledged, the depth of its intracellular role remains an active area of investigation. The proteolytic cleavage of membrane-spanning proteins, like APLP1, is a sophisticated and multitiered cellular event. It not only shapes the functionalities of these proteins but also produces bioactive segments that can steer diverse cellular routes. The APLP1 life cycle critically hinges on its cleavage by γ-secretase, generating an APLP1-ICD fragment with multifaceted intracellular roles, including a speculated role in gene modulation. Such cleavage pathways are not arbitrary degradation events; they are meticulously regulated, resulting in peptides or fragments with distinct biological roles. When debating the efficacy of APLP1 as a biomarker, especially in the context of EVs, it is paramount to discern between the full-length proteins and their cleaved versions. Their genesis and prevalence can vary markedly in the bloodstream. Our preliminary data underscore that while cleaved APLP1 fragments do circulate in blood, the APLP1 content within EVs, especially APLP1^+^ EVs, is overwhelmingly cerebral (Fig. 4D). This distinction is not only pivotal but also reinforces the diagnostic potential of APLP1^+^ EVs in neurodegenerative conditions. Moreover, the encapsulation of APLP1 within EVs might offer a shield against extraneous cleavage, ensuring its preservation within the vesicular milieu until its intended destination. To encapsulate, while APLP1’s cleavage via γ-secretase is a well-established biological phenomenon, the context and final destination of the resultant fragments are decisive in evaluating its biomarker utility. Our findings compellingly argue for the diagnostic prowess of APLP1^+^ EVs in the domain of neurodegenerative disease diagnostics, primarily due to their neural origins.

It is also imperative to note that L1CAM has garnered attention in oncology, being implicated in the genesis and metastatic progression of diverse cancers (*62*–*66*). Through rigorous in silico analysis (Fig. 2), we ascertained the superior specificity of APLP1 over L1CAM as a cerebral-centric surface marker for BDEV isolation. Notably, APLP1 demonstrated pronounced and selective expression within the brain (Fig. 3). Distinct from L1CAM, APLP1 exhibited an augmented expression in EVs derived from brain tissue as compared to the brain tissue itself (Fig. 4D). Consequently, our data compellingly endorse APLP1 as an emergent marker for BDEVs.

Our systematic exploration into the cellular origin of APLP1^+^ EVs has been thorough. Given the diverse cellular composition of the neural environment, we broadened our validation approach (Figs. 3F, 4I, and 5B). While the hippocampal region was our primary focus, we also delved into the cortical domains to capture a holistic view. We initiated our study using well-established markers like NeuN. Recognizing the intricate nature of neuronal markers, we incorporated an array of neuron-specific indicators such as Tuj1, enolase 2 (ENO2), MAP2, and NeuN. Recent data suggest a robust expression of APLP1 in oligodendrocytes in both mouse and human brains (fig. S2). This prompted us to further investigate the APLP1’s presence in different cerebral regions. Our preliminary data indicate a potential association of APLP1 with neurons, oligodendrocytes, and certain astrocytes, hinting at a predominant brain origin for APLP1^+^ cells (Figs. 3F and 5B). While our findings provide valuable insights into the potential brain-specific nature of APLP1^+^ EVs, we maintain a prudent stance. We believe that a more comprehensive understanding requires further in-depth studies and diverse methodological approaches.

Building on these foundational insights, we delved deeper into the functional characteristics and significance of APLP1^+^ EVs. Through a comprehensive strategy involving BDEV quantification, protein profiling, and small RNA-seq analysis, we discerned patterns indicative of APLP1^+^ EVs in the blood mirroring BDEV traits (Figs. 5 and 6). Further validation came from the observation of enhanced neuronal marker expression in APLP1^+^ EVs derived from plasma (Fig. 5). These collective results bolster the proposition that APLP1 might serve as a pivotal marker for distinguishing BDEVs amidst the vast assortment of EVs in the blood.

Using host cell–specific EVs as diagnostic tools presents a compelling paradigm shift in disease detection and management. One salient advantage is traceability. While free proteins or cell-free nucleic acids in circulation offer diagnostic insights, their origins remain elusive. This lack of cellular origin specificity can cloud diagnostic clarity, potentially leading to erroneous interpretations (*65*). A case in point is the carbohydrate antigen 19-9, a notable marker for pancreatic ductal adenocarcinoma. Its overexpression in a spectrum of cells beyond the pancreas often muddies diagnostic waters, resulting in misinterpretations associated with hepatocellular, gastrointestinal, thyroid, and even salivary gland malignancies (*67*). Yet, the real allure of host cell–specific EVs lies in their cargo. The molecular constituents within these vesicles paint an intricate picture of the host cell’s physiological state, offering invaluable diagnostic and prognostic cues (*68*). Our exploratory endeavor into small RNA profiling of blood-derived BDEVs, isolated using APLP1, resonates with this sentiment. The abundance of brain-centric miRNAs within APLP1^+^ EVs and their reflection of cerebral conditions (Fig. 6, B and F) are not just mere observations but a testament to the potential diagnostic prowess of this approach. A critical point of introspection in our study revolves around the potential overlap between APLP1^+^ EVs and residual EVs. Given that residual EVs are defined as the fraction remaining post-APLP1^+^ EV enrichment from plasma EVs, there exists a plausible avenue for inadvertent coexistence of trace APLP1^+^ EVs within this residual subset, especially if they eluded detection during the immunoprecipitation process. Such overlap, albeit minimal, holds implications for the integrity of our cargo analysis. Essentially, when dissecting the molecular profile of residual EVs, we must be cognizant of the possibility that some constituents might be reflective of APLP1^+^ EVs. This could potentially elucidate the simultaneous detection of certain factors in both APLP1^+^ EVs and residual EVs, as observed in Fig. 6. While our findings are robust, this subtle nuance underscores the need for even more refined isolation techniques in future investigations. While our research robustly positions APLP1 as a brain-specific BDEV marker, given the significant enrichment of brain-associated proteins and miRNAs in APLP1^+^ EVs, the broader landscape of brain disease diagnosis via BDEVs remains intricate and demands further contemplation. A pressing concern in leveraging BDEVs for diagnosis is the potential contamination from proteins prolific in blood—albumins, complement proteins, lipoproteins, and globulins come to mind, not to mention the potential influence of platelet-derived EVs. As we delve deeper into the molecular makeup of BDEVs, it becomes apparent that the signature of disease-specific markers could be overshadowed by these abundant proteins and miRNAs intrinsic to platelet-derived EVs (*69*, *70*). Traditional EV isolation techniques, while reliable, present their own sets of challenges. Ultracentrifugation, revered for its limited protein contamination, is an arduous and inefficient endeavor. Size exclusion chromatography, hailed for its simplicity and speed, grapples with distinguishing nanoparticles akin in size to EVs and exhibits a high propensity for protein contamination (*71*). Immunoaffinity purification, contingent upon specific markers, is not exempt from challenges either, particularly with the specter of nonspecific antibody binding and interference from plasma proteins (*71*, *72*). While using treatments like proteinase K, trypsin, and other proteases during EV isolation may curb contamination, it is a double-edged sword, potentially compromising the integrity of EV surface antigens and subsequent analyses.

Nevertheless, in juxtaposition to these methodologies, BDEV utilization promises a relatively uncontaminated profile. However, the path is not devoid of challenges. Consider the conundrum of pathogenic proteins—tau, amyloid-β, and α-synuclein are just a few that manifest ubiquitously across a spectrum of degenerative ailments (*73*). The diagnostic terrain mandates a nuanced approach, possibly a symphony of multiple biomarkers for precise disease identification (*74*, *75*). While APLP1 stands as a formidable contender in the realm of BDEV markers, its true potential might be realized in concert with a suite of other biomarkers, orchestrating a harmonized, multibiomarker system to pierce through the complexities of brain disease diagnosis. The realm of neurodiagnostics is evolving rapidly, with the pursuit of precision at its core. Multibiomarker systems tailored to specific brain diseases are emerging as the vanguard of this evolution. Consider AD as an exemplar. While APLP1 stands as a promising biomarker, its true diagnostic potential might be amplified when assessed in tandem with AD hallmarks like the amyloid-β precursor, another pertinent EV membrane marker. Such a synergistic approach promises a more nuanced diagnostic lens, potentially enhancing the specificity and sensitivity of AD detection. Similarly, for conditions like PD, the diagnostic narrative is enriched when APLP1 is coupled with dopamine transporter (DAT), a marker quintessential to dopaminergic neurons. Such a composite approach is poised to elevate the diagnostic fidelity for PD, offering clinicians a more granular view of the disease’s molecular intricacies. However, the true litmus test for APLP1^+^ EVs lies in their clinical validation. Ongoing research endeavors are meticulously dissecting the sensitivity and specificity of APLP1^+^ EVs, juxtaposing the profiles of pathogenic proteins within these vesicles between patients harboring brain diseases and healthy counterparts. The preliminary insights from these endeavors are poised to not only ratify the diagnostic potential of APLP1^+^ EVs but also to illuminate their broader applicability across a spectrum of neurological conditions. In light of our research, APLP1 has emerged not just as a mere biomarker but as a cerebral-specific sentinel, adept at discerning and enriching BDEVs. This discovery transcends mere academic interest; it has profound clinical ramifications. Harnessing APLP1 could revolutionize the diagnostic landscape, offering a window into the early stages of brain diseases. This approach promises not only sensitivity but also a noninvasive and cost-effective modality, a potent combination that could reshape neurodiagnostic paradigms. The horizon beckons exploration, and APLP1^+^ EVs might offer a meaningful stride toward enriching our diagnostic toolkit. But the potential of APLP1^+^ EVs extends further. They could underpin a pioneering platform for early brain disease diagnosis, one that intimately mirrors the intricate neuropathological trajectories. As we reflect upon our findings, the vision is clear: to elevate this research from bench to bedside, creating a scaffold for early intervention in myriad neurological conditions.

## MATERIALS AND METHODS

### Isolation and culture of human neuronal progenitor cells

Human neuronal cells were derived from central nervous system tissues of spontaneously aborted fetuses (female) during gestational weeks 10, 12, and 14, following informed maternal consent. Before utilization, fetal tissues underwent rigorous screenings as per the criteria set in Annex II of the EMEA Commission Directive 2006/17/EC. This included tests for pathogens such as human immunodeficiency virus 1/2, hepatitis B and C, *Treponema pallidum*, cytomegalovirus, and *Toxoplasma gondii*. Furthermore, the endotoxin levels in these tissues were meticulously assessed. Only cells from fetuses devoid of the aforementioned pathogens were selected for cultivation. The procurement and application of fetal tissues were sanctioned by the Institutional Review Board (IRB; 2009-06-074 and 2015-08-130) of CHA University/General Hospital, Gyeonggi-do, Korea. The isolation and subsequent culture of primary neuronal progenitor cells adhered to the protocols delineated by Moon *et al*. (*42*) . The culture medium of these human neuronal cells was harvested every 2 to 3 days for EV isolation.

### Generation of midbrain organoid using human primary neuronal progenitor cells

The primary neuronal progenitor cells, previously generated and characterized in a prior study by Moon *et al.* (*42*) in 2017, were cultured. Subsequently, these cells were resuspended in an aggregation medium [culture medium including 0.4% (v/v) polyvinyl alcohol; Sigma-Aldrich, St. Louis, MO, USA, catalog no. 363170]. This suspension was then seeded at a density of 10,000 cells per well in a 96-well V-bottom plate (Greiner Bio-One, Kremsmünster, Austria, catalog nos. 651161 and 656161). Within 48 hours of incubation, the cells underwent three-dimensional (3D) formation. After the neuronal cells successfully formed a 3D structure, they progressed to the ventral midbrain patterning stage as part of our ongoing research methodology. This was achieved by replacing the existing culture medium with a specialized dopaminergic neuron induction medium. This induction medium, designed to promote dopaminergic differentiation, was formulated on the basis of neurobasal medium (Thermo Fisher Scientific, Waltham, MA, USA, catalog no. 21103-049). The medium was enriched with a variety of additives. These included GlutaMAX [1% (v/v), Thermo Fisher Scientific, catalog no. 35050-061], the B-27 supplement [1% (v/v), Thermo Fisher Scientific, catalog no. 17504044], forskolin (10 mM, Sigma-Aldrich, catalog no. F6886), a picolinic acid derivative (50 mM, Sigma-Aldrich, catalog no. P42800), and dibutyryl cyclic adenosine monophosphate (100 mM, Sigma-Aldrich, catalog no. D0627).

### Animals

All animal procedures received approval from the CHA University Institutional Animal Care and Use Committee (no. IACUC190134). Mice were maintained at ambient room temperature (RT), following a 12/12-hour light/dark cycle. The C57BL/6 strain was used to assess RNA and protein expression across various organs, encompassing the brain, heart, kidneys, spleen, and liver. In addition, EVs were extracted from this strain. For the collection of brain and blood samples, Thy-1 GFP M line mice [M Tg (Thy-1EGFP)MJrs/J, on a C57BL/6J background, strain no. 007788] were procured from the Jackson Laboratory (Bar Harbor, ME, USA).

### Integrative bioinformatic approach for biomarker discovery and APLP1 expression analysis

We used public databases, including the HPA (proteinatlas.org), GO, ExoCarta, Vesiclepedia, and Brain RNA-seq (brainrnaseq.org), for data acquisition and analysis. Our biomarker discovery approach began with data retrieval from HPA, which was then subjected to GO analysis, cross-referenced with ExoCarta and Vesiclepedia, and subsequently validated via Brain RNA-seq (Fig. 2A). To investigate the expression of APLP1, gene expression profiles specific to different brain cell types were sourced from Brain RNA-seq. In addition, single-cell data for the human brain were procured from the Single-cell atlas (http://adsn.ddnetbio.com). Comprehensive gene expression profiles and tissue staining data for various human organs were gathered from the HPA.

### Clinical samples

To assess the diagnostic potential of APLP1 in brain diseases, we enlisted patients with GBM and healthy volunteers. All participants provided written informed consent, and the research protocol was sanctioned by the IRB (2017P001581) of Massachusetts General Hospital Harvard University, Massachusetts, USA. Plasma samples were procured from both newly diagnosed or patients with recurrent GBM (*n* = 3) and healthy counterparts (*n* = 3). Cancer diagnoses were validated through neuropathological evaluations and clinical imaging. Collection of clinical samples for isolation of APLP1^+^ EV from human plasma was approved by the IRB of the CHA Bundang Medical Center (Korea) (2021-03-056-010 and 2018-05-015).

### Preparation of plasma

Mouse blood was drawn from the lateral tail veins and centrifuged at 3000*g* for 15 min at RT, and pooled plasma samples from the same strain were instantly frozen at −80°C. For humans, blood was collected in K2 EDTA-coated tubes (BD Biosciences, Franklin Lakes, NJ, USA, catalog no. BD 367856), centrifuged at 3000*g* for 30 min at RT. The resultant plasma was stored at −80°C, undergoing a single freeze-thaw cycle at RT.

### Histological assays

For horseradish peroxidase (HRP)–DAB staining, brain sections underwent treatments with 0.3% Triton X-100 (Sigma-Aldrich, catalog no. T9284) and 1% hydrogen peroxide (Merck Millipore, Burlington, MA, USA, catalog no. H1009). After blocking with 10% normal goat serum (Vector Laboratories, Burlingame, CA, USA, catalog no. S-1000-20), sections were incubated with rabbit anti-APLP1 (1:500 dilution; Abcam, Cambridge, UK, catalog no. ab192481; RRID: AB_2827651) overnight at 4°C. This was followed by an hour’s exposure to antirabbit HRP (1:1000 dilution; Jackson ImmunoResearch Laboratories, West Grove, PA, USA, catalog no. 111-035-003; RRID: AB_2313567) for 1 hour at RT. The sections were then reacted with a DAB Kit (Vector Laboratories) for 1 to 2 min. For fluorescent IHC, post–Triton X-100 (Sigma-Aldrich, catalog no. T9284) and 10% goat serum treatments (Vector Laboratories, catalog no. SK-4100), sections were concurrently incubated with primary antibodies: rabbit anti-APLP1 (1:500 dilution; Abcam, catalog no. ab192481; RRID: AB_2827651) and mouse anti-L1CAM (1:100 dilution; Santa Cruz Biotechnology, Dallas, TX, USA, catalog no. sc-514360) at 4°C overnight. Subsequently, they were exposed to Alexa Fluor 488– or Alexa Fluor 594–conjugated secondary antibodies (1:500 dilution; Thermo Fisher Scientific, catalog nos. A11001 and A11012; RRID: AB_2534069 and RRID: AB_2534079) for 1 hour at RT. After a brief 4′,6-diamidino-2-phenylindole staining (Thermo Fisher Scientific, catalog no. D1306) for 5 min at RT in the dark, sections were mounted using ProLong Gold Antifade Mountant (Thermo Fisher Scientific, catalog no. P36931).

### PCR and qPCR

The tissue total RNA was prepared from the brain, heart, kidneys, spleen, and liver of C57BL/6 mice using TRIzol reagent (Ambion, Austin, TX, USA, catalog no. 15596-018). The RNA of EVs was extracted using a miRVana Paris Kit (Thermo Fisher Scientific, catalog no. AM1556), and all RNAs were extracted according to the manufacturer’s recommended protocol. cDNA was synthesized from the total RNA using reverse transcriptase (SuperScript II Reverse Transcriptase; Thermo Fisher Scientific, catalog no. 18064014). Primers (table S4) for *APLP1*, *L1CAM*, *ENO2*, *Tuj1*, *MAP2*, and *GAPDH* genes were designed to perform PCR and qPCR. For qPCR, 10 μM primer mixture, SYBR-Green with low ROX (Enzynomics, Daejeon, Korea, catalog no. RT500M), nuclease-free water (Ambion, catalog no. AM9938), and a cDNA template were used in a final volume of 20 μl. Using a Step One Real-Time PCR system (Applied Biosystems, Foster City, CA, USA, catalog no. 4375816), qPCR was performed with an initial denaturation/activation step at 95°C, followed by 40 cycles of 15 s at 95°C, 30 s at 60°C, and 20 s at 72°C. Quantification of gene expression was based on the Ct value for each sample.

### Western blot

Protein samples were extracted using radioimmunoprecipitation assay buffer (iNtRON Biotechnology, Seongnam-si, Gyeonggi-do, Korea, catalog no. IBS-BR002) with a protease cocktail tablet (Roche, Basel, Switzerland, catalog no. 11697498001) and phosphatase inhibitors II and III (Sigma-Aldrich, catalog nos. P5726 and P0044). Protein concentrations were measured using a bicinchoninic acid (BCA) assay (Thermo Fisher Scientific, catalog no. 23225). The proteins were denatured via incubation of samples for 5 min at 95°C, after which the equal amounts of protein (20 to 30 μg) were loaded onto 10% SDS–polyacrylamide gel electrophoresis for Western blot assays. Subsequently, 10% skim milk blocking solution was diluted in Tris Buffered Saline with Tween 20 (TBS-T) and used to treat the samples for 1 hour. The antibodies used in these experiments were as follows: mouse anti-L1CAM (clone c-2; 1:1000 dilution; Santa Cruz Biotechnology, catalog no. sc-514360), rabbit anti-APLP1 (1:1000 dilution; Abcam, catalog no. ab192481; RRID: AB_2827651), mouse anti–glyceraldehyde phosphate dehydrogenase (1:10,000 dilution; Santa Cruz Biotechnology, catalog no. sc-32233; RRID: AB_627679), mouse anti-CD81 (1:1000 dilution; Abcam, catalog no. ab79559; RRID: AB_1603682), mouse anti-CD9 (1:1000 dilution; Abcam, catalog no. ab92726; RRID: AB_10561589), mouse anti-CD63 (1:1000 dilution; Santa Cruz Biotechnology, catalog no. sc-5275; RRID: AB_627877), mouse anti-TSG101 (1:1000 dilution; Santa Cruz Biotechnology, catalog no. sc-7964; RRID: AB_671392), mouse anti-calnexin (1:1000 dilution; Abcam, catalog no. ab22595; RRID: AB_2069006), mouse anti-vinculin (1:1000 dilution; Abcam, catalog no. ab130007; RRID: AB_11156698), mouse anti-MAP2 (1:1000 dilution; Santa Cruz Biotechnology, catalog no. sc-390543), and mouse anti-Tuj1 (1:10,000 dilution; BioLegend, San Diego, CA, USA, catalog no. 801201; RRID: AB_2313773). These primary antibodies were diluted in TBS-T and incubated overnight at 4°C. antirabbit or antimouse HRP-conjugated secondary antibodies (1:5000 dilution; Jackson ImmunoResearch Laboratories, catalog no. 111-035-003; RRID: AB_2313567) were then incubated with the samples for 1 hour at RT, and immunoreactivity was detected using an enhanced chemiluminescent HRP substrate (Merck Millipore, catalog no. WBKLS0500).

### RNAscope in situ hybridization with IHC

RNAscope in situ hybridization (ISH) kits were purchased from Advanced Cell Diagnostics (ACD; Hayward, CA, USA). The combination of ISH with IHC was performed according to the manufacturer’s instructions. Briefly, the frozen tissues were sectioned at a thickness of 15 μm. These sections were then mounted on Superfrost Plus slides (Epredia, Kalamazoo, MI, USA, catalog no. J1800AMNZ) and baked at 60°C for 30 min. Following this, the sections were immersed in cold 4% paraformaldehyde (Abelbio, Seoul, Korea, catalog no. AB102-134) for a duration of 15 min. The tissue sections were dehydrated through a series of 5-min incubations in progressively higher concentrations of molecular-grade ethanol: 50%, 75%, and two rounds at 100%. Subsequently, the sections were treated with RNAscope H_2_O_2_ from ACD (catalog no. 322335) for 15 min at RT. This was followed by an incubation in RNAscope Target Retrieval reagent (ACD, catalog no. 323165) for 5 min at 98°C. To demarcate each section, boundaries were drawn using a hydrophobic pen (ImmEdge PAP pen; Vector Laboratories, catalog no. 310018). After the hydrophobic boundaries were set and dried, the tissue sections were incubated in primary antibody solutions, diluted with Co-Detection Diluent (ACD, catalog no. 323160), overnight at 4°C. The primary antibodies applied were as follows: 1:100 mouse anti-NeuN (EMD Millipore, catalog no. MAB377; RRID: AB_2298772), 1:100 mouse anti-GFAP (Dako, Santa Clara, CA, USA, catalog no. Z0334; RRID: AB_10013382), 1:50 rabbit anti-olig2 (Merck Millipore, catalog no. ab9610; RRID: AB_570666), and 1:100 rabbit anti-Iba-1 (Fujifilm Wako Pure Chemical, Osaka, Japan, catalog no. 019-19741; RRID: AB_839504). Following the overnight incubation, each section was covered with protease plus reagent (ACD, catalog no. 322331). To ensure protein epitope preservation for IHC labeling, sections underwent a 30-min protease digestion. The sections were then hybridized with RNAscope buffered Z probes for APLP1 (ACD, catalog no. 827951), with either a positive control or a negative control (ACD, catalog nos. 320881 and 32087), for 2 hours at 40°C. To amplify the probe signal, sections were sequentially incubated in AMP1 (ACD, catalog no. 323101) for 30 min at 40°C, followed by AMP2 (ACD, catalog no. 323102) for another 30 min at 40°C and then AMP3 (ACD, catalog no. 323103) for 15 min at 40°C. After this, the sections were treated with the far-red dye Opal570 (Akoya Bio, Delaware, catalog no. FP1488001KT) for 30 min at 40°C. They were then exposed to a channel-specific HRP blocker (ACD, catalog no. 323107) to seal the amplifier structure. Subsequently, the slides were incubated with secondary antibody solutions for 30 min. The secondary antibodies used were 1:100 goat antimouse Alexa Fluor 488 (Thermo Fisher Scientific, catalog no. A11001; RRID: AB_2534069) and 1:100 goat antirabbit Alexa Fluor 488 (Thermo Fisher Scientific, catalog no. A11008; RRID: AB_143165), both diluted in Co-Detection Diluent (ACD, catalog no. 323160).

### Isolation of EVs

Brain tissue–EVs were isolated via ultracentrifugation as previously described (*76*). Plasma EVs were derived from both animal plasma (C57BL/6 and Thy-1 GFP M line mice) and human plasma, sourced either from Innovative Research (Plymouth, MN, USA) or clinical volunteers. For EV isolation, 0.5 ml of plasma was processed using a qEV original column (Izon Science Ltd., Burnside, Christchurch, New Zealand, catalog no. ICO-70). Initial elutions (fractions 1 to 6) were discarded, while the EV-enriched fractions 7 to 10 were collected, pooled, and filtered using a 0.22-μm Ultrafree Centrifugal Filter device (0.22-μm pore size; Merck Millipore, catalog no. UFC30GV0S). The pooled fractions were subsequently concentrated using a 100-kDa Amicon Ultra device (Merck Millipore, catalog no. UFC801096). Neuronal cell–derived EVs were isolated from culture media. Following a centrifugation at 2000*g* for 10 min at RT to remove cell debris, the clarified medium was loaded onto a qEV 2-ml column (Izon Science Ltd., catalog no. IC2-70). The pooled EV-rich fractions (7 to 10) were then concentrated using an Amicon Ultra-15 centrifugal filter with a 100-kDa threshold (Merck Millipore, catalog no. UFC901096).

### Immunolabeling and preparation of plasma EVs for particle analysis

A 20-μl aliquot of the plasma EV suspension was mixed with 80 μl of phosphate-buffered saline (PBS) in a 1.5-ml amber reaction tube to achieve thorough dilution. Subsequently, 100 μl of the diluted plasma EVs was exposed to rabbit anti-APLP1 at a 1:100 dilution (Abcam, catalog no. ab192481; RRID: AB_2827651) and incubated for 1 hour at RT within the same tube. Following this, the EVs underwent a 30-min incubation at RT with Alexa Fluor 594–conjugated secondary antibodies, diluted 1:200 (Thermo Fisher Scientific, catalog no. A11012; RRID: AB_2534079). To eliminate unbound antibodies, the mixture was processed through Zeba Spin Desalting Columns with a 7KDa molecular weight cutoff (Thermo Fisher Scientific, catalog no. 89882). The labeled EVs were then collected in a fresh 1.5-ml amber reaction tube and resuspended in 900 μl of PBS. This final suspension was readied for subsequent particle measurement.

### Nanoparticle tracking analysis

EVs were diluted in PBS (1-ml final volume) and examined under a ZetaView Nanoparticle Tracking Video Microscope (Particle Metrix, Inning, Germany). Ideal measurement concentrations were found by pretesting the ideal particle per frame value (140 to 200 particles per frame). The software manufacturer’s default settings for EVs or nanoparticles were selected. For each measurement, 3 cycles were performed by scanning 11 cell positions and capturing 60 frames per position (video setting: high). The following settings were used: focus, autofocus; camera sensitivity for all samples, 80.0; shutter, 100; scattering intensity, 1.2; and cell temperature, 23°C. After capture, the videos were analyzed using the built-in ZetaView Software 8.02.31 with the following parameters: maximum particle size, 1000; minimum particle size, 5.

### Fluorescence staining of plasma EVs

Plasma EVs (0.1 μg) were biotinylated with EZ-Link Sulfo-NHS-LC LC-Biotin (Thermo Fisher Scientific, catalog no. A35358). The remaining free biotins were removed using Zeba Spin Desalting Columns (7K MWCO; Thermo Fisher Scientific, catalog no. 89882), and 20 μl of biotinylated EVs were then incubated on a streptavidin-coated glass slide (Arrayit Corporation, Sunnyvale, CA, USA, catalog no. SMSF) for 30 min. The EVs were fixed using BD Fix Perm (BD Biosciences, catalog no. BD554722) and blocked with 0.2% bovine serum albumin (BSA)–PBS. Immunofluorescence staining was performed using the primary antibodies for 2 hours at RT, after which incubation with Alexa Fluor 488– or Alexa Fluor 594–conjugated secondary antibodies (1:400 dilution; Thermo Fisher Scientific, catalog nos. A11001 and 11012; RRID: AB_2534069 and RRID: AB_2534079) was conducted for 1 hour at RT. The primary antibodies used in these experiments were as follows: rabbit anti-APLP1 (1:100 dilution; Abcam, catalog no. ab94957; RRID: AB_10890629), mouse anti-CD63 (1:100 dilution; Ancell Corporation, Bayport, MN, USA, catalog no. 215-820), mouse anti-CNPase (1:100 dilution; Merck Millipore, catalog no. MAB326; RRID: AB_2082608), mouse anti-L1CAM (1:20 dilution; Santa Cruz Biotechnology, catalog no. sc-514360), mouse anti-CD11b (1:25 dilution; AbD Serotec, NC, USA, catalog no. MCA74G; RRID: AB_321293), mouse anti-GLAST (1:100 dilution; Merck Millipore, catalog no. MABN794; RRID: AB_2811303).

### Enrichment of BDEVs from plasma EVs

For the enrichment of BDEVs, rabbit anti-APLP1 (Abcam, catalog no. 94957; RRID: AB_10890629) was biotinylated using a Pierce Antibody Biotinylation Kit (Thermo Fisher Scientific, catalog no. 90407) according to the manufacturer’s recommended protocol. Plasma EVs were then incubated with 4 μg of biotinylated APLP1 for 60 min at 4°C in a rotating mixer. The immune complex was captured using streptavidin-agarose UltraLink Resin (Thermo Fisher Scientific, catalog no. 53116) for 60 min at RT with continuous mixing. The streptavidin-bound complex was centrifuged at 200*g* for 10 min to separate the pellet and the supernatant. Then, the supernatant was analyzed for residual EVs. The pellet was resuspended in 200 μl of 0.1 M glycine-HCl (pH 2.5) via mixing for 10 s, after which centrifugation was conducted at 4500*g* and 4°C for 10 min to detach APLP1^+^ EVs from the bead-antibody complex. Supernatants were transferred to clean tubes containing 15 μl of 1 M tris-HCl (pH 8.0) and mixed, after which the tubes were stored at −80°C.

### Mass spectrometry and proteome data analysis

The mass spectrometry analysis was performed at the Beijing Genomics Institution (Shenzhen, China). All mass spectrometry experiments used a Q-Exactive HF X (Thermo Fisher Scientific) for data-dependent acquisition. The peptides separated by liquid phase chromatography were ionized by a nano electrospray ionization (nano ESI) source. The main parameters were set as follows: ion source voltage, 1.9 kV; MS1 scanning range, 350 to 1500 mass/charge ratio (*m/z*); resolution, 60,000; MS2 starting *m/z* 100; resolution, 15,000; ion screening conditions for MS2 fragmentation charge, 2+ to 6+; and the top 30 parent ions with the peak intensity exceeding 10,000. The ion fragmentation mode was higher-energy collisional dissociation (HCD), and the fragment ions were detected in Orbitrap. The dynamic exclusion time was set to 30 s. The automatic gain control (AGC) was set to MS1 3E6 and MS2 1E5. The MS raw files were processed using the MaxQuant software. To align with the UniProt human proteome database (82,427 sequences), including the MaxQuant contaminants, we set the following search parameters: variable modifications included oxidation (M), acetylation (protein N terminus), deamidation (NQ), and deamidation 18O (N), while carbamidomethyl (C) was specified as a fixed modification. In addition, we enabled the “Match-between-runs” option and the “Label-free quantification” option, with a label-free quantification (LFQ) min ratio count of 1. Trypsin/P was selected as the cleavage enzyme, allowing for up to two missed cleavages. All other options were kept at MaxQuant default settings. To investigate the biological function of proteins, GO term analysis was performed using the Database for Annotation, Visualization and Integrated Discovery (DAVID) (*77*).

### Small RNA-seq of EVs

Small RNA-seq was performed in sorted EVs using biotinylated APLP1 antibody from human plasma EVs. The sequencing was performed at the Beijing Genomics Institution, applying the DNBseq platform. The sequencing data were aligned to the human reference genome (GRCh38) using a subread aligner (*78*), and miRNA read counts were obtained with the featureCounts program (*79*). Subsequently, miRNA counts were normalized, and low-expressed miRNAs were filtered out using the edgeR R package. miRNA target analysis was conducted using miRNet2.0, and functional enrichment analysis of target genes was performed using miEAA (*80*).

### ELISA of human plasma EVs

Human plasma EVs were isolated using a qEV column (Izon Science Ltd., catalog no. ICO-70) according to the manufacturer’s instructions. The protein concentration of EVs was measured using a microBCA Protein Assay Kit (Thermo Fisher Scientific, catalog no. 23235). Collected EVs were diluted to 5 μg/ml in enzyme-linked immunosorbent assay (ELISA) coating buffer (Sigma-Aldrich, catalog no. C3041) and captured on NUNC MaxiSorp 96-well plates (Thermo Fisher Scientific, catalog no. 44-2404-21) overnight at 4°C. After washing with PBS-T (0.1% Tween 20 in PBS), the plates were blocked with 1% BSA-PBS for 2 hours at RT. After discarding the blocking solution, primary antibodies in blocking solution were added to the wells, and incubation was performed overnight at 4°C. Unbound antibodies were washed out with PBS-T three times, and the wells were incubated with HRP-conjugated secondary antibodies in blocking solution for 2 hours at RT. After washing three times with PBS-T, 100 μl of one-step Ultra TMB-Blotting Solution (Thermo Fisher Scientific, catalog no. 34028) was added to each well and incubated for 1 hour at RT. After sufficient color development, the absorbance of each well at 620 nm was measured using a plate reader (PerkinElmer, Waltham, MA, USA). The antibodies used for the ELISA were as follows: antihuman CD63 (1:1500 dilution; Ancell, catalog no. 215-820), antihuman CD9 (1:1500 dilution; BD Biosciences, catalog no. 555370; RRID: AB_395772), antihuman CD81 (1:1500 dilution; BD Biosciences, catalog no. 555675; RRID: AB_396028), antihuman L1CAM (1:500 dilution; Cell Signaling Technology, Danvers, MA, USA, catalog no. 89861S; RRID: AB_2800145), antihuman APLP1 (1:500 dilution; Abcam, catalog no. ab94957; RRID: AB_10890629), antimouse immunoglobulin G (IgG) secondary HRP (1:500 dilution; Cell Signaling Technology, catalog no. 7076S; RRID: AB_330924), and antirabbit IgG secondary HRP (1:500 dilution; Cell Signaling Technology, catalog no. 7074S; RRID: AB_2099233).

### Statistical analysis

Statistical analysis was performed using R basic functions and lme4, an R package for either *t* test or analysis of variance (ANOVA) followed by a Tukey post hoc test. Data are presented as the means ± SE, and a *P* value of < 0.05 was considered significant.
