# Supplementary material for: Blood-derived APLP1+ extracellular vesicles are potential biomarkers for the early diagnosis of brain diseases
Source: Sci Adv. 2025 Jan 1;11(1):eado6894. doi: 10.1126/sciadv.ado6894 (PMC11691634; doi:10.1126/sciadv.ado6894)
Supplement: Supplementary file 1 — Figs. S1 to S5 Tables S1 to S5 [file sciadv.ado6894_sm.pdf]

Science Advances
AAAS

Supplementary Materials for

Blood-derived APLP1^{+} extracellular vesicles are potential biomarkers for the early diagnosis of brain diseases

Yuri Choi *et al.*

Corresponding author: Jisook Moon, jmoon@cha.ac.kr

Sci. Adv. **11**, eado6894 (2025)
DOI: 10.1126/sciadv.ado6894

This PDF file includes:

Figs. S1 to S5
Tables S1 to S5

Fig. S1.

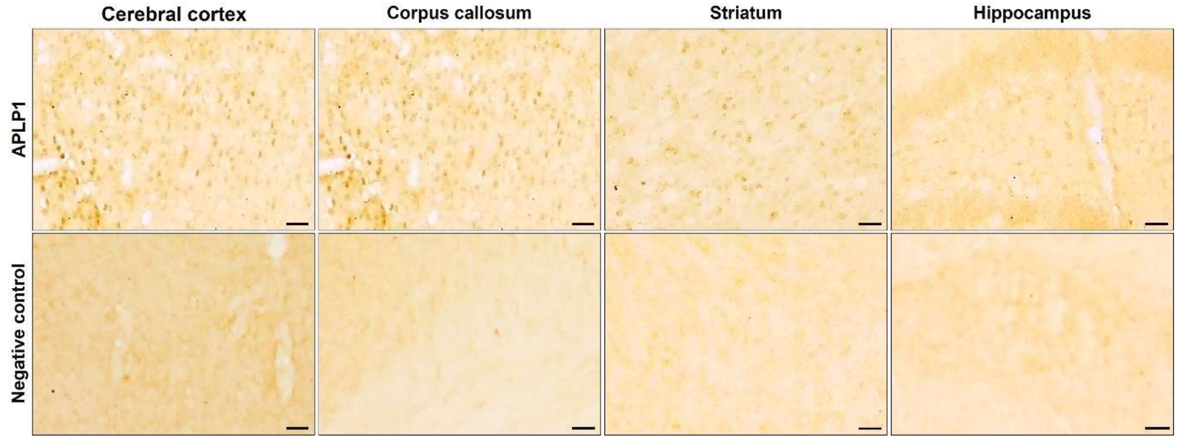

Fig. S1. APLP1 Expression in Various Mouse Brain Regions. APLP1 expression in different regions of mouse brain tissue, as captured through immunohistochemistry staining. The positive signals, manifesting as brown dots, are the result of DAB staining observed throughout the brain tissue. Conversely, the negative control sections were selectively subjected to staining with secondary antibodies only. The staining procedure was independently conducted on seven specimens of C57BL/6 mice. The scale bar denotes 50 μm.

Fig. S2.

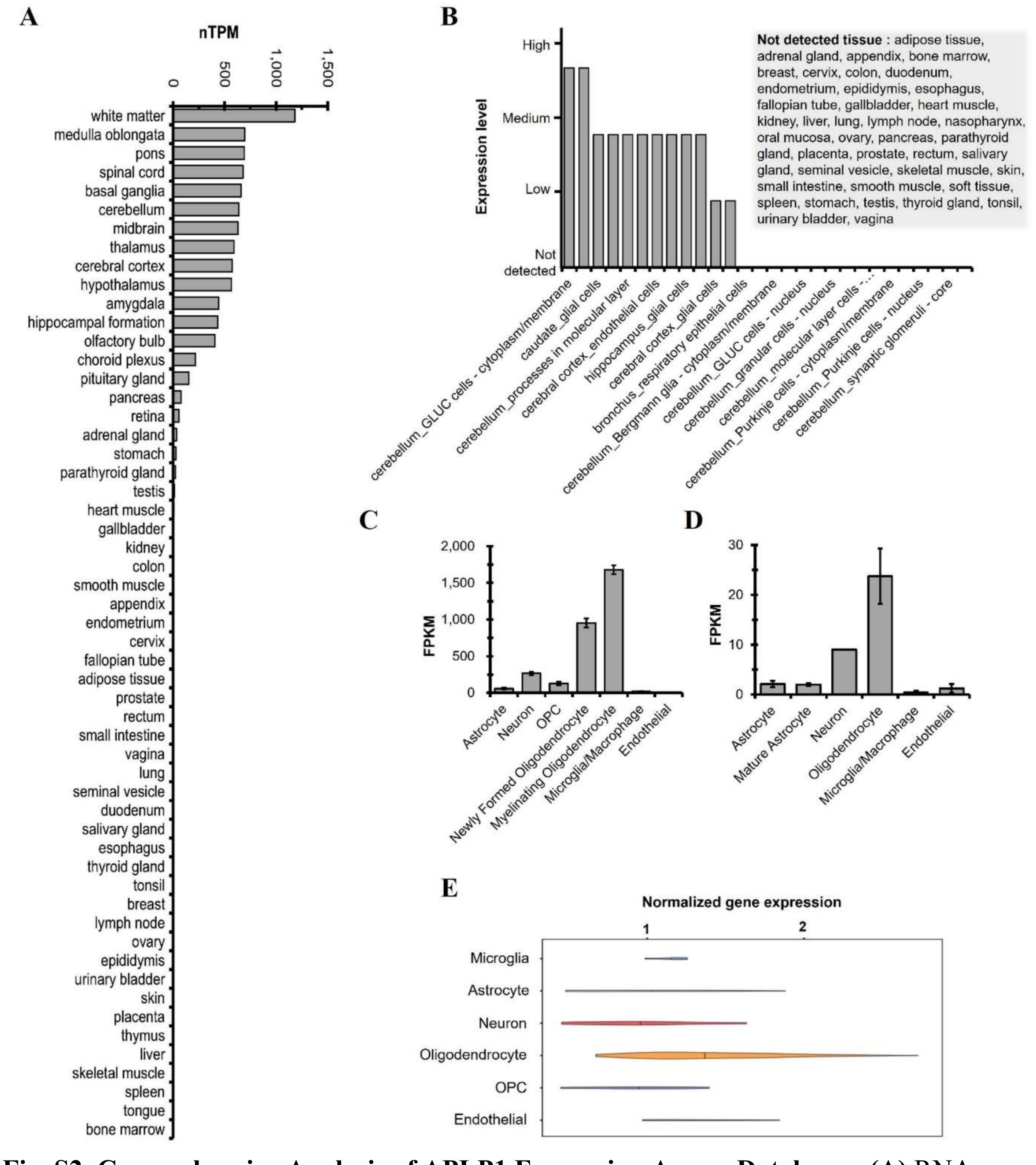

Fig. S2. Comprehensive Analysis of APLP1 Expression Across Databases. (A) RNA expression levels of APLP1 in various human tissues, sourced from the Human Protein Atlas (HPA). The y-axis presents the type of tissues, while the x-axis represents normalized transcripts per million (nTPM). **(B)** APLP1 protein expression levels in different human tissues based on immunohistochemical staining from the HPA dataset. Tissues that were not stained are listed separately in the empty space of the graph. The staining intensity for each tissue is differentiated into four levels: high, medium, low, and not detected. **(C and D)** RNA expression levels of APLP1 by cell type in the mouse brain and in the human brain, sourced from the Cell Types

Database (https://www.brainrnaseq.org). The y-axis shows fragments per kilobase of transcript per million (FPKM). **(E)** Cell type-specific APLP1 expression levels in the human brain, based on data from the Single-cell atlas (http://adsn.ddnetbio.com). The x-axis represents log-normalized counts.

Fig. S3.

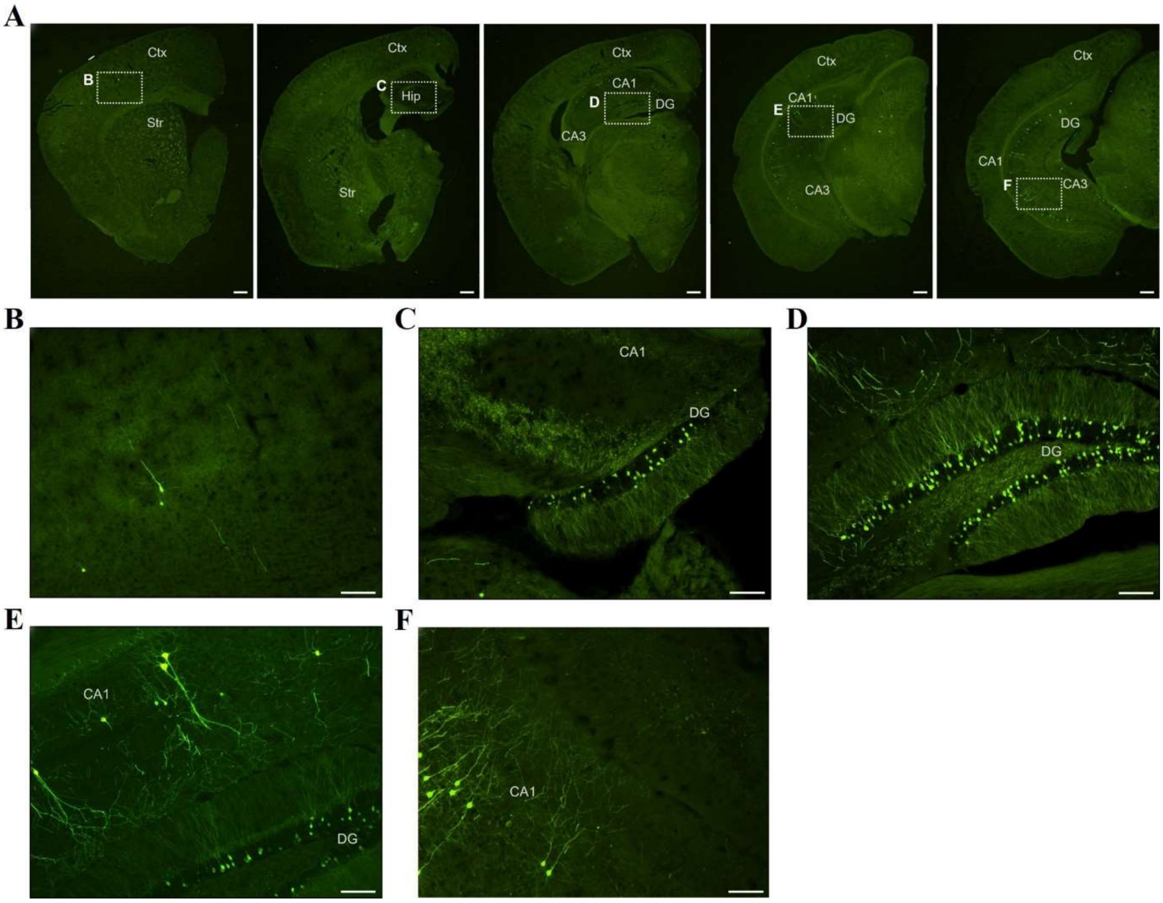

Fig. S3. GFP Expression Across Various Brain Regions in Thy-1 GFP Mice. (A) Whole brain images obtained from three distinct Thy-1 GFP mice show the varied expression of GFP across different brain regions. **(B)** In the cerebral cortex, a limited number of neurons are labeled. **(C-F)** In the hippocampus, a substantial number of neurons, including pyramidal cells, mossy fibers, and granule cells, are labeled, notably in the hippocampal subfield CA1 and the dentate gyrus. The scale bar represents 200 μm **(A)** and 50 μm **(B-F)**. The experiment was performed with five Thy-1 GFP mice. Abbreviations: CTX — cerebral cortex; Str—Striatum; Hip—hippocampus; CA1 and CA3—hippocampal subfield; DG—dentate gyrus and hippocampal subfield.

Fig. S4.

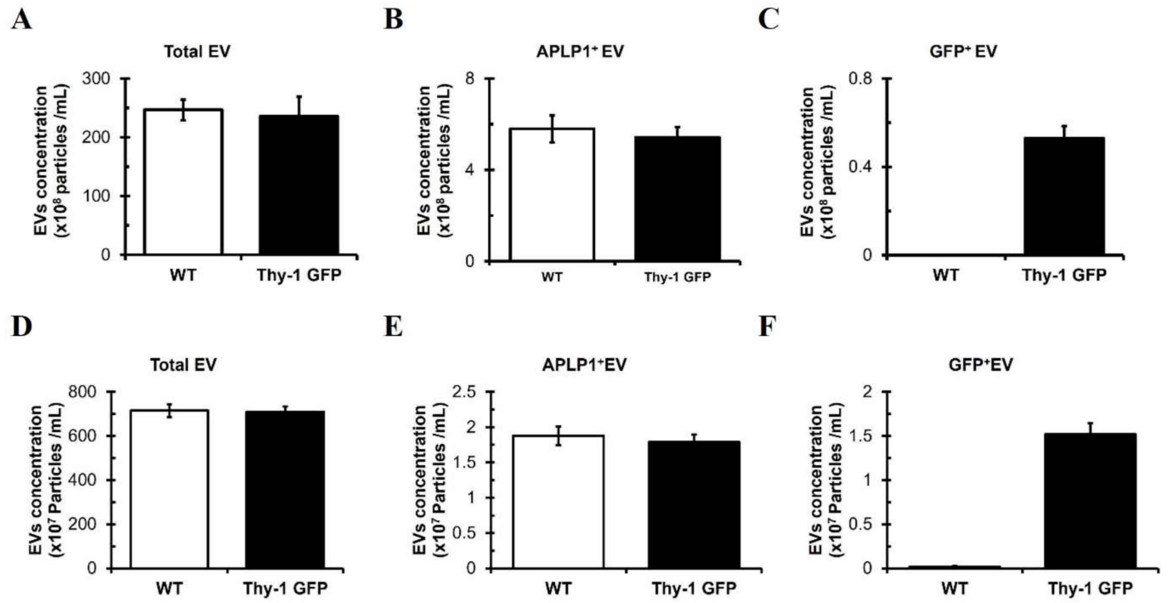

Fig. S4. Concentration Analysis of Various EV Types. This figure illustrates the concentration levels of different types of extracellular vesicles (EVs) - specifically, total EV, $APLP1^{+}$ EV, and GFP^{+} EV - found in the plasma samples collected from both Wild Type (WT) and Thy-1 GFP M line mice. The EVs were quantified through nanoparticle tracking analysis, following either a staining procedure **(A-C)** or a pull-down method **(D-F)**. The error bars represent the ± SEM from at least three technical replicates of measurements in pooled plasma derived-EVs.

Fig. S5.

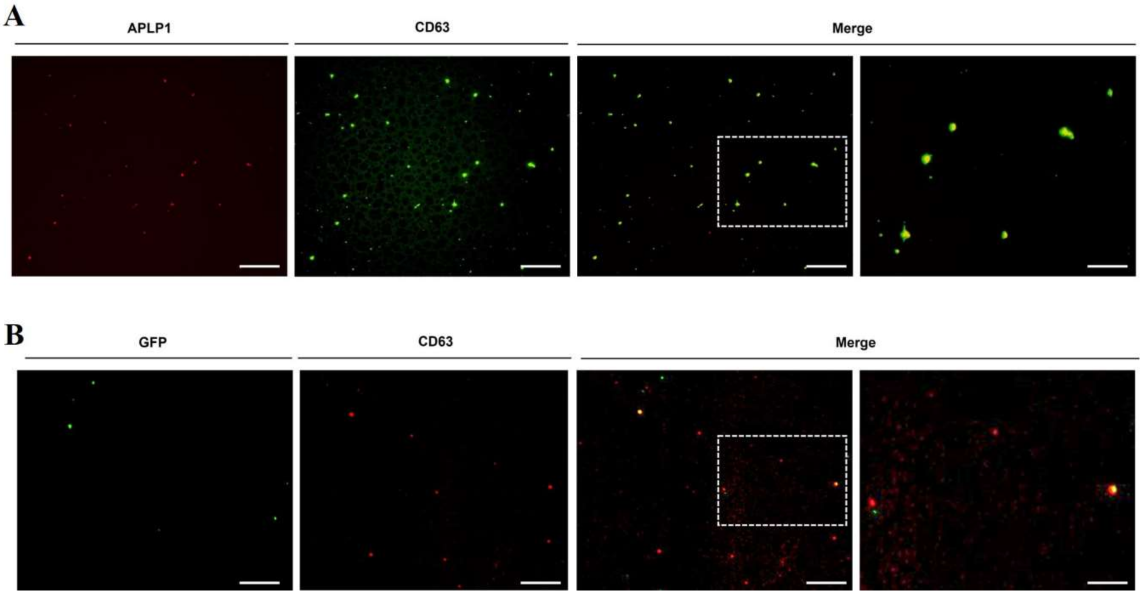

Fig. S5. Fluorescence Staining Images of Mouse Plasma EVs. (A) Representative images of mouse plasma-EVs stained with APLP1 (red fluorescence) and CD63 (green fluorescence) antibodies. **(B)** Fluorescence image of GFP (green fluorescence) and CD63 (red fluorescence) in plasma EVs from Thy-1 GFP M line mice. The experiment was independently performed from EVs of five mice. Scale bar represents 50 μm.

Table S1. Brain-Specific Proteins for BDEV Marker Identification.

The table lists brain-specific proteins used for identifying potential BDEV markers as illustrated in Fig. 2B. Data was extracted from the Human Protein Atlas database (https://www.proteinatlas.org/), latest accessed in April 2021. Since the raw data extracted from the Human Protein Atlas database is listed, some blanks present.

Gene	Ensembl	Gene description	Uniprot
GFAP	ENSG00000131095	Glial fibrillary acidic protein	P14136
TLX3	ENSG00000164438	T cell leukemia homeobox 3	O43711
NEUROD2	ENSG00000171532	Neuronal differentiation 2	Q15784
NEUROD6	ENSG00000164600	Neuronal differentiation 6	Q96NK8
NCAN	ENSG00000130287	Neurocan	O14594
MOG	ENSG00000204655	Myelin oligodendrocyte glycoprotein	Q16653
HPCA	ENSG00000121905	Hippocalcin	P84074
AVP	ENSG00000101200	Arginine vasopressin	P01185
BARHL1	ENSG00000125492	BarH like homeobox 1	Q9BZE3
FGF3	ENSG00000186895	Fibroblast growth factor 3	P11487
MBP	ENSG00000197971	Myelin basic protein	P02686
MEPE	ENSG00000152595	Matrix extracellular phosphoglycoprotein	Q9NQ76
OPALIN	ENSG00000197430	Oligodendrocytic myelin paranodal and inner loop protein	Q96PE5
OMG	ENSG00000126861	Oligodendrocyte myelin glycoprotein	P23515
HAPLN2	ENSG00000132702	Hyaluronan and proteoglycan link protein 2	Q9GZV7
GRIN1	ENSG00000176884	Glutamate ionotropic receptor NMDA type subunit 1	Q05586
CAMKV	ENSG00000164076	CaM kinase like vesicle associated	Q8NCB2
MOBP	ENSG00000168314	Myelin-associated oligodendrocyte basic protein	Q13875
HCRT	ENSG00000161610	Hypocretin neuropeptide precursor	O43612
SPHAR	ENSG00000213029	S-phase response (cyclin related)	Q15513
PMP2	ENSG00000147588	Peripheral myelin protein 2	P02689
C1orf61	ENSG00000125462	Chromosome 1 open reading frame 61	Q13536
CACNG7	ENSG00000105605	Calcium voltage-gated channel auxiliary subunit gamma 7	P62955
GRM4	ENSG00000124493	Glutamate metabotropic receptor 4	Q14833
KCNJ9	ENSG00000162728	Potassium voltage-gated channel subfamily J member 9	Q92806
AMER2	ENSG00000165566	APC membrane recruitment protein 2	Q8N7J2
PLP1	ENSG00000123560	Proteolipid protein 1	P60201
TBR1	ENSG00000136535	T-box, brain 1	Q16650
TMEM235	ENSG00000204278	Transmembrane protein 235	A6NFC5
GABRD	ENSG00000187730	Gamma-aminobutyric acid type A receptor delta subunit	O14764
GAP43	ENSG00000172020	Growth associated protein 43	P17677
TTC9B	ENSG00000174521	Tetratricopeptide repeat domain 9B	Q8N6N2
GRM3	ENSG00000198822	Glutamate metabotropic receptor 3	Q14832
SNCB	ENSG00000074317	Synuclein beta	Q16143
GABRA6	ENSG00000145863	Gamma-aminobutyric acid type A receptor alpha6 subunit	Q16445
POU3F2	ENSG00000184486	POU class 3 homeobox 2	P20265
GABRA1	ENSG00000022355	Gamma-aminobutyric acid type A receptor alpha1 subunit	P14867
HRH3	ENSG00000101180	Histamine receptor H3	Q9Y5N1
SNAP25	ENSG00000132639	Synaptosome associated protein 25	P60880
CREG2	ENSG00000175874	Cellular repressor of E1A stimulated genes 2	Q8IUH2
GABRA5	ENSG00000186297	Gamma-aminobutyric acid type A receptor alpha5 subunit	P31644
HTR2C	ENSG00000147246	5-hydroxytryptamine receptor 2C	P28335
SULT4A1	ENSG00000130540	Sulfotransferase family 4A member 1	Q9BR01
SYN2	ENSG00000157152	Synapsin II	
ATP6V1G2	ENSG00000213760	ATPase H+ transporting V1 subunit G2	O95670

GPR6	ENSG00000146360	G protein-coupled receptor 6	P46095
GRIN2B	ENSG00000273079	Glutamate ionotropic receptor NMDA type subunit 2B	Q13224
LRTM2	ENSG00000166159	Leucine rich repeats and transmembrane domains 2	Q8N967
TNR	ENSG00000116147	Tenascin R	Q92752
CACNG3	ENSG00000006116	Calcium voltage-gated channel auxiliary subunit gamma 3	O60359
FBXL16	ENSG00000127585	F-box and leucine rich repeat protein 16	Q8N461
GPR37L1	ENSG00000170075	G protein-coupled receptor 37 like 1	O60883
KIF5A	ENSG00000155980	Kinesin family member 5A	Q12840
RTP5	ENSG00000188011	Receptor transporter protein 5 (putative)	Q14D33
ANKRD63	ENSG00000230778	Ankyrin repeat domain 63	C9JTQ0
KCNF1	ENSG00000162975	Potassium voltage-gated channel modifier subfamily F member 1	Q9H3M0
PDYN	ENSG00000101327	Prodynorphin	P01213
STMN4	ENSG00000015592	Stathmin 4	Q9H169
SYN1	ENSG00000008056	Synapsin I	P17600
ZDHHC22	ENSG00000177108	Zinc finger DHHC-type containing 22	Q8N966
ZIC1	ENSG00000152977	Zic family member 1	Q15915
GPR101	ENSG00000165370	G protein-coupled receptor 101	Q96P66
PTPN5	ENSG00000110786	Protein tyrosine phosphatase, non-receptor type 5	P54829
SLC17A7	ENSG00000104888	Solute carrier family 17 member 7	Q9P2U7
ZIC2	ENSG00000043355	Zic family member 2	O95409
BCAN	ENSG00000132692	Brevican	Q96GW7
CNTNAP4	ENSG00000152910	Contactin associated protein like 4	Q9C0A0
NRGN	ENSG00000154146	Neurogranin	Q92686
PCDHGC5	ENSG00000240764	Protocadherin gamma subfamily C, 5	Q9Y5F6
POU3F4	ENSG00000196767	POU class 3 homeobox 4	P49335
STX1B	ENSG00000099365	Syntaxin 1B	P61266
TMEM88B	ENSG00000205116	Transmembrane protein 88B	A6NKF7
AC079341.3	ENSG00000284849		
C11orf87	ENSG00000185742	Chromosome 11 open reading frame 87	Q6NUJ2
PSD2	ENSG00000146005	Pleckstrin and Sec7 domain containing 2	Q9BQI7
SLC17A6	ENSG00000091664	Solute carrier family 17 member 6	Q9P2U8
SLC6A3	ENSG00000142319	Solute carrier family 6 member 3	Q01959
TPH2	ENSG00000139287	Tryptophan hydroxylase 2	Q8IWU9
ZIC4	ENSG00000174963	Zic family member 4	Q8N9L1
CBLN3	ENSG00000139899	Cerebellin 3 precursor	Q6UW01
CPLX1	ENSG00000168993	Complexin 1	O14810
ELAVL3	ENSG00000196361	ELAV like RNA binding protein 3	Q14576
RIMS1	ENSG00000079841	Regulating synaptic membrane exocytosis 1	Q86UR5
SHANK1	ENSG00000161681	SH3 and multiple ankyrin repeat domains 1	Q9Y566
UNCX	ENSG00000164853	UNC homeobox	A6NJT0
VSTM2B	ENSG00000187135	V-set and transmembrane domain containing 2B	A6NLU5
ZIC3	ENSG00000156925	Zic family member 3	O60481
APC2	ENSG00000115266	APC2, WNT signaling pathway regulator	O95996
BTBD17	ENSG00000204347	BTB domain containing 17	A6NE02
C1QL3	ENSG00000165985	Complement C1q like 3	Q5VWW1
C4orf50	ENSG00000181215	Chromosome 4 open reading frame 50	Q6ZRC1
CASKIN1	ENSG00000167971	CASK interacting protein 1	Q8WXD9
DPF1	ENSG00000011332	Double PHD fingers 1	Q92782
DRD1	ENSG00000184845	Dopamine receptor D1	P21728
GABBR2	ENSG00000136928	Gamma-aminobutyric acid type B receptor subunit 2	O75899
GPR52	ENSG00000203737	G protein-coupled receptor 52	Q9Y2T5
KCNC1	ENSG00000129159	Potassium voltage-gated channel subfamily C member 1	P48547
KCNN1	ENSG00000105642	Potassium calcium-activated channel subfamily N member 1	Q92952

MGAT5B	ENSG00000167889	Mannosyl (alpha-1,6-)-glycoprotein beta-1,6-N-acetyl-glucosaminyltransferase, isozyme B	Q3V5L5
MT3	ENSG00000087250	Metallothionein 3	P25713
NCDN	ENSG00000020129	Neurochondrin	Q9UBB6
NETO1	ENSG00000166342	Neuropilin and tolloid like 1	Q8TDF5
NTSR2	ENSG00000169006	Neurotensin receptor 2	O95665
PAQR6	ENSG00000160781	Progestin and adipoQ receptor family member 6	Q6TCH4
RESP18	ENSG00000182698	Regulated endocrine specific protein 18	Q5W5W9
SEZ6	ENSG00000063015	Seizure related 6 homolog	Q53EL9
TMEM151A	ENSG00000179292	Transmembrane protein 151A	Q8N4L1
AL590132.1	ENSG00000284299		
BAALC	ENSG00000164929	BAALC, MAP3K1 and KLF4 binding	Q8WXS3
BHLHA9	ENSG00000205899	Basic helix-loop-helix family member a9	Q7RTU4
CACNG2	ENSG00000166862	Calcium voltage-gated channel auxiliary subunit gamma 2	Q9Y698
CPNE9	ENSG00000144550	Copine family member 9	Q8IYJ1
DNM1	ENSG00000106976	Dynamin 1	Q05193
ERMN	ENSG00000136541	Ermin	Q8TAM6
GPM6A	ENSG00000150625	Glycoprotein M6A	P51674
GPR62	ENSG00000180929	G protein-coupled receptor 62	Q9BZJ7
MDGA1	ENSG00000112139	MAM domain containing glycosylphosphatidylinositol anchor 1	Q8NFP4
PMCH	ENSG00000183395	Pro-melanin concentrating hormone	P20382
PRKCG	ENSG00000126583	Protein kinase C gamma	P05129
RIT2	ENSG00000152214	Ras like without CAAX 2	Q99578
SLC1A3	ENSG00000079215	Solute carrier family 1 member 3	P43003
SYT1	ENSG00000067715	Synaptotagmin 1	P21579
TMEM132D	ENSG00000151952	Transmembrane protein 132D	Q14C87
TMEM59L	ENSG00000105696	Transmembrane protein 59 like	Q9UK28
ZP2	ENSG00000103310	Zona pellucida glycoprotein 2	Q05996
ADGRB2	ENSG00000121753	Adhesion G protein-coupled receptor B2	O60241
B3GAT1	ENSG00000109956	Beta-1,3-glucuronyltransferase 1	Q9P2W7
C8orf46	ENSG00000169085	Chromosome 8 open reading frame 46	Q8TAG6
CABP1	ENSG00000157782	Calcium binding protein 1	Q9NZU7
CACNA1A	ENSG00000141837	Calcium voltage-gated channel subunit alpha1 A	O00555
CEND1	ENSG00000184524	Cell cycle exit and neuronal differentiation 1	Q8N111
CSPG5	ENSG00000114646	Chondroitin sulfate proteoglycan 5	O95196
GLRA1	ENSG00000145888	Glycine receptor alpha 1	P23415
GNG3	ENSG00000162188	G protein subunit gamma 3	P63215
GPR26	ENSG00000154478	G protein-coupled receptor 26	Q8NDV2
IL1RAPL1	ENSG00000169306	Interleukin 1 receptor accessory protein like 1	Q9NZN1
JPH3	ENSG00000154118	Junctophilin 3	Q8WXH2
KIF3C	ENSG00000084731	Kinesin family member 3C	O14782
OTP	ENSG00000171540	Orthopedia homeobox	Q5XKR4
OXT	ENSG00000101405	Oxytocin/neurophysin I prepropeptide	P01178
SLITRK1	ENSG00000178235	SLIT and NTRK like family member 1	Q96PX8
SRRM4	ENSG00000139767	Serine/arginine repetitive matrix 4	A7MD48
SYT11	ENSG00000132718	Synaptotagmin 11	Q9BT88
VAX1	ENSG00000148704	Ventral anterior homeobox 1	Q5SQQ9
ABCA2	ENSG00000107331	ATP binding cassette subfamily A member 2	Q9BZC7
AMBN	ENSG00000178522	Ameloblastin	Q9NP70
AMER3	ENSG00000178171	APC membrane recruitment protein 3	Q8N944
B3GAT2	ENSG00000112309	Beta-1,3-glucuronyltransferase 2	Q9NPZ5
CDH18	ENSG00000145526	Cadherin 18	Q13634
CNTN2	ENSG00000184144	Contactin 2	Q02246

CORT	ENSG00000241563	Cortistatin	O00230
DISP2	ENSG00000140323	Dispatched RND transporter family member 2	A7MBM2
ENC1	ENSG00000171617	Ectodermal-neural cortex 1	O14682
FAM181B	ENSG00000182103	Family with sequence similarity 181 member B	A6NEQ2
FSTL5	ENSG00000168843	Follistatin like 5	Q8N475
GABRG1	ENSG00000163285	Gamma-aminobutyric acid type A receptor gamma1 subunit	Q8N1C3
GRM5	ENSG00000168959	Glutamate metabotropic receptor 5	P41594
HTR6	ENSG00000158748	5-hydroxytryptamine receptor 6	P50406
ICAM5	ENSG00000105376	Intercellular adhesion molecule 5	Q9UMF0
KCNQ3	ENSG00000184156	Potassium voltage-gated channel subfamily Q member 3	O43525
KCNV1	ENSG00000164794	Potassium voltage-gated channel modifier subfamily V member 1	Q6PIU1
MRGPRE	ENSG00000184350	MAS related GPR family member E	Q86SM8
MYT1	ENSG00000196132	Myelin transcription factor 1	Q01538
NKAIN2	ENSG00000188580	Sodium/potassium transporting ATPase interacting 2	Q5VXU1
NRXN1	ENSG00000179915	Neurexin 1	P58400, Q9ULB1
NRXN2	ENSG00000110076	Neurexin 2	P58401, Q9P2S2
OLFM3	ENSG00000118733	Olfactomedin 3	Q96PB7
PCDH8	ENSG00000136099	Protocadherin 8	O95206
PNMA2	ENSG00000240694	PNMA family member 2	Q9UL42
RASL10A	ENSG00000100276	RAS like family 10 member A	Q92737
SCRT1	ENSG00000261678	Scratch family transcriptional repressor 1	Q9BWW7
03-Sep	ENSG00000100167	Septin 3	Q9UH03
SHISA7	ENSG00000187902	Shisa family member 7	A6NL88
SLC32A1	ENSG00000101438	Solute carrier family 32 member 1	Q9H598
SLC5A7	ENSG00000115665	Solute carrier family 5 member 7	Q9GZV3
SV2C	ENSG00000122012	Synaptic vesicle glycoprotein 2C	Q496J9
SYT3	ENSG00000213023	Synaptotagmin 3	Q9BQG1
TMEM151B	ENSG00000178233	Transmembrane protein 151B	Q8IW70
TPPP	ENSG00000171368	Tubulin polymerization promoting protein	O94811
TRIM9	ENSG00000100505	Tripartite motif containing 9	Q9C026
VSNL1	ENSG00000163032	Visinin like 1	P62760
ASIC1	ENSG00000110881	Acid sensing ion channel subunit 1	P78348
CHRNB2	ENSG00000160716	Cholinergic receptor nicotinic beta 2 subunit	P17787
CNIH2	ENSG00000174871	Cornichon family AMPA receptor auxiliary protein 2	Q6PI25
DIRAS2	ENSG00000165023	DIRAS family GTPase 2	Q96HU8
DNAJC6	ENSG00000116675	DnaJ heat shock protein family (Hsp40) member C6	O75061
GABRB2	ENSG00000145864	Gamma-aminobutyric acid type A receptor beta2 subunit	P47870
GBX2	ENSG00000168505	Gastrulation brain homeobox 2	P52951
GSX1	ENSG00000169840	GS homeobox 1	Q9H4S2
HPCAL4	ENSG00000116983	Hippocalcin like 4	Q9UM19
KCNH1	ENSG00000143473	Potassium voltage-gated channel subfamily H member 1	O95259
KLHL1	ENSG00000150361	Kelch like family member 1	Q9NR64
LHFPL3	ENSG00000187416	LHFPL tetraspan subfamily member 3	Q86UP9
MAP2	ENSG00000078018	Microtubule associated protein 2	P11137
NPTX1	ENSG00000171246	Neuronal pentraxin 1	Q15818
OTOL1	ENSG00000182447	Otolin 1	A6NHN0
PCDHGC4	ENSG00000242419	Protocadherin gamma subfamily C, 4	Q9Y5F7
PRMT8	ENSG00000111218	Protein arginine methyltransferase 8	Q9NR22
RTN1	ENSG00000139970	Reticulon 1	Q16799
SCN2A	ENSG00000136531	Sodium voltage-gated channel alpha subunit 2	Q99250
SLC1A2	ENSG00000110436	Solute carrier family 1 member 2	P43004
SLC35F1	ENSG00000196376	Solute carrier family 35 member F1	Q5T1Q4
SNAP91	ENSG00000065609	Synaptosome associated protein 91	O60641

ST18	ENSG00000147488	ST18, C2H2C-type zinc finger	O60284
SYNPR	ENSG00000163630	Synaptoporin	Q8TBG9
SYP	ENSG00000102003	Synaptophysin	P08247
AC091980.2	ENSG00000275038		
ADGRB1	ENSG00000181790	Adhesion G protein-coupled receptor B1	O14514
AK5	ENSG00000154027	Adenylate kinase 5	Q9Y6K8
ATCAY	ENSG00000167654	ATCAY, caytaxin	Q86WG3
CACNA1E	ENSG00000198216	Calcium voltage-gated channel subunit alpha1 E	Q15878
CBLN1	ENSG00000102924	Cerebellin 1 precursor	P23435
COL20A1	ENSG00000101203	Collagen type XX alpha 1 chain	Q9P218
CYP46A1	ENSG00000036530	Cytochrome P450 family 46 subfamily A member 1	Q9Y6A2
DLL3	ENSG00000090932	Delta like canonical Notch ligand 3	Q9NYJ7
ELFN2	ENSG00000166897	Extracellular leucine rich repeat and fibronectin type III domain containing 2	Q5R3F8
FEZF2	ENSG00000153266	FEZ family zinc finger 2	Q8TBJ5
GALNT9	ENSG00000182870	Polypeptide N-acetylgalactosaminyltransferase 9	Q9HCQ5
GDAP1L1	ENSG00000124194	Ganglioside induced differentiation associated protein 1 like 1	Q96MZ0
GJC2	ENSG00000198835	Gap junction protein gamma 2	Q5T442
GPM6B	ENSG00000046653	Glycoprotein M6B	Q13491
GRIA2	ENSG00000120251	Glutamate ionotropic receptor AMPA type subunit 2	P42262
GRIA4	ENSG00000152578	Glutamate ionotropic receptor AMPA type subunit 4	P48058
GRM2	ENSG00000164082	Glutamate metabotropic receptor 2	Q14416
HTR2A	ENSG00000102468	5-hydroxytryptamine receptor 2A	P28223
KCTD4	ENSG00000180332	Potassium channel tetramerization domain containing 4	Q8WVF5
LINGO1	ENSG00000169783	Leucine rich repeat and Ig domain containing 1	Q96FE5
LRFN2	ENSG00000156564	Leucine rich repeat and fibronectin type III domain containing 2	Q9ULH4
LRRTM2	ENSG00000146006	Leucine rich repeat transmembrane neuronal 2	O43300
LY6H	ENSG00000176956	Lymphocyte antigen 6 family member H	O94772
MAP1A	ENSG00000166963	Microtubule associated protein 1A	P78559
NAPB	ENSG00000125814	NSF attachment protein beta	Q9H115
NEFL	ENSG00000277586	Neurofilament light	P07196
NEUROD1	ENSG00000162992	Neuronal differentiation 1	Q13562
NPTXR	ENSG00000221890	Neuronal pentraxin receptor	O95502
OPCML	ENSG00000183715	Opioid binding protein/cell adhesion molecule like	Q14982
PDZD4	ENSG00000067840	PDZ domain containing 4	Q76G19
PHACTR3	ENSG00000087495	Phosphatase and actin regulator 3	Q96KR7
PRRT1	ENSG00000204314	Proline rich transmembrane protein 1	Q99946
RAB3A	ENSG00000105649	RAB3A, member RAS oncogene family	P20336
RIMS3	ENSG00000117016	Regulating synaptic membrane exocytosis 3	Q9UJD0
RPRML	ENSG00000179673	Reprimo like	Q8N4K4
SAMD14	ENSG00000167100	Sterile alpha motif domain containing 14	Q8IZD0
08-Sep	ENSG00000164402	Septin 8	Q92599
SKOR1	ENSG00000188779	SKI family transcriptional corepressor 1	P84550
SOX8	ENSG00000005513	SRY-box 8	P57073
SRRM3	ENSG00000177679	Serine/arginine repetitive matrix 3	
TMEM132B	ENSG00000139364	Transmembrane protein 132B	Q14DG7
TRIM67	ENSG00000119283	Tripartite motif containing 67	Q6ZTA4
YJEFN3	ENSG00000250067	YjeF N-terminal domain containing 3	A6XGL0
ACTL6B	ENSG00000077080	Actin like 6B	O94805
AKAP5	ENSG00000179841	A-kinase anchoring protein 5	P24588
AL109810.2	ENSG00000235710		
ARNT2	ENSG00000172379	Aryl hydrocarbon receptor nuclear translocator 2	Q9HBZ2
BHLHE22	ENSG00000180828	Basic helix-loop-helix family member e22	Q8NFJ8
BRINP1	ENSG00000078725	BMP/retinoic acid inducible neural specific 1	O60477

C1QL2	ENSG00000144119	Complement C1q like 2	Q7Z5L3
C1QTNF4	ENSG00000172247	C1q and TNF related 4	Q9BXJ3
CADM2	ENSG00000175161	Cell adhesion molecule 2	Q8N3J6
CCDC177	ENSG00000267909	Coiled-coil domain containing 177	Q9NQR7
CDR1	ENSG00000184258	Cerebellar degeneration related protein 1	P51861
CELF4	ENSG00000101489	CUGBP Elav-like family member 4	Q9BZC1
CTNND2	ENSG00000169862	Catenin delta 2	Q9UQB3
DGKB	ENSG00000136267	Diacylglycerol kinase beta	Q9Y6T7
FAM131B	ENSG00000159784	Family with sequence similarity 131 member B	Q86XD5
FGF17	ENSG00000158815	Fibroblast growth factor 17	O60258
FGFBP3	ENSG00000174721	Fibroblast growth factor binding protein 3	Q8TAT2
FOXG1	ENSG00000176165	Forkhead box G1	P55316
GABRA3	ENSG00000011677	Gamma-aminobutyric acid type A receptor alpha3 subunit	P34903
GABRG2	ENSG00000113327	Gamma-aminobutyric acid type A receptor gamma2 subunit	P18507
GAD1	ENSG00000128683	Glutamate decarboxylase 1	Q99259
GNAO1	ENSG00000087258	G protein subunit alpha o1	P09471
GRIA1	ENSG00000155511	Glutamate ionotropic receptor AMPA type subunit 1	P42261
GRID2IP	ENSG00000215045	Grid2 interacting protein	A4D2P6
KCNA1	ENSG00000111262	Potassium voltage-gated channel subfamily A member 1	Q09470
KCNC2	ENSG00000166006	Potassium voltage-gated channel subfamily C member 2	Q96PR1
KCND2	ENSG00000184408	Potassium voltage-gated channel subfamily D member 2	Q9NZV8
KCNK4	ENSG00000182450	Potassium two pore domain channel subfamily K member 4	Q9NYG8
LRRC4C	ENSG00000148948	Leucine rich repeat containing 4C	Q9HCJ2
MAPK8IP2	ENSG00000008735	Mitogen-activated protein kinase 8 interacting protein 2	Q13387
MICAL2	ENSG00000133816	Microtubule associated monooxygenase, calponin and LIM domain containing 2	O94851, Q6ZW33
MSANTD3-TMEFF1	ENSG00000251349	MSANTD3-TMEFF1 readthrough	
NDST4	ENSG00000138653	N-deacetylase and N-sulfotransferase 4	Q9H3R1
NPBWR2	ENSG00000125522	Neuropeptides B and W receptor 2	P48146
NPFFR1	ENSG00000148734	Neuropeptide FF receptor 1	Q9GZQ6
NR2E1	ENSG00000112333	Nuclear receptor subfamily 2 group E member 1	Q9Y466
NSMF	ENSG00000165802	NMDA receptor synaptonuclear signaling and neuronal migration factor	Q6X4W1
OLFM1	ENSG00000130558	Olfactomedin 1	Q99784
PAK5	ENSG00000101349	P21 (RAC1) activated kinase 5	Q9P286
PLK5	ENSG00000185988	Polo like kinase 5	Q496M5
PLPPR4	ENSG00000117600	Phospholipid phosphatase related 4	Q7Z2D5
PPFIA2	ENSG00000139220	PTPRF interacting protein alpha 2	O75334
PRKAR1B	ENSG00000188191	Protein kinase cAMP-dependent type I regulatory subunit beta	P31321
PRR18	ENSG00000176381	Proline rich 18	Q8N4B5
PRR35	ENSG00000161992	Proline rich 35	P0CG20
PTPRZ1	ENSG00000106278	Protein tyrosine phosphatase, receptor type Z1	P23471
RLN2	ENSG00000107014	Relaxin 2	P04090
RUNDC3A	ENSG00000108309	RUN domain containing 3A	Q59EK9
SCN3B	ENSG00000166257	Sodium voltage-gated channel beta subunit 3	Q9NY72
SERPINI1	ENSG00000163536	Serpin family I member 1	Q99574
SH3GL2	ENSG00000107295	SH3 domain containing GRB2 like 2, endophilin A1	Q99962
SLC12A5	ENSG00000124140	Solute carrier family 12 member 5	Q9H2X9
SLIT1	ENSG00000187122	Slit guidance ligand 1	O75093
SMIM17	ENSG00000268182	Small integral membrane protein 17	P0DL12
STXBP1	ENSG00000136854	Syntaxin binding protein 1	P61764
SV2B	ENSG00000185518	Synaptic vesicle glycoprotein 2B	Q7L1I2
SYT2	ENSG00000143858	Synaptotagmin 2	Q8N9I0
TAGLN3	ENSG00000144834	Transgelin 3	Q9UI15

TMEM144	ENSG00000164124	Transmembrane protein 144	Q7Z5S9
TTYH1	ENSG00000167614	Tweety family member 1	Q9H313
TUBB2A	ENSG00000137267	Tubulin beta 2A class IIa	Q13885
UCHL1	ENSG00000154277	Ubiquitin C-terminal hydrolase L1	P09936
YWHAH	ENSG00000128245	Tyrosine 3-monooxygenase/tryptophan 5-monooxygenase activation protein eta	Q04917
ZNF536	ENSG00000198597	Zinc finger protein 536	O15090
AC005726.1	ENSG00000258472		
AC245033.1	ENSG00000260836		
AMPH	ENSG00000078053	Amphiphysin	P49418
ANKRD34A	ENSG00000272031	Ankyrin repeat domain 34A	Q69YU3
APLP1	ENSG00000105290	Amyloid beta precursor like protein 1	P51693
ATP1B2	ENSG00000129244	ATPase Na+/K+ transporting subunit beta 2	P14415
BRSK1	ENSG00000160469	BR serine/threonine kinase 1	Q8TDC3
BSN	ENSG00000164061	Bassoon presynaptic cytomatrix protein	Q9UPA5
BTBD8	ENSG00000189195	BTB domain containing 8	Q5XKL5
CADPS2	ENSG00000081803	Calcium dependent secretion activator 2	Q86UW7
CAMKK2	ENSG00000110931	Calcium/calmodulin dependent protein kinase kinase 2	Q96RR4
CDH10	ENSG00000040731	Cadherin 10	Q9Y6N8
CDH22	ENSG00000149654	Cadherin 22	Q9UJ99
CDH7	ENSG00000081138	Cadherin 7	Q9ULB5
CDK5R1	ENSG00000176749	Cyclin dependent kinase 5 regulatory subunit 1	Q15078
CELF5	ENSG00000161082	CUGBP Elav-like family member 5	Q8N6W0
CERS1	ENSG00000223802	Ceramide synthase 1	P27544
CHN1	ENSG00000128656	Chimerin 1	P15882
CLVS2	ENSG00000146352	Clavesin 2	Q5SYC1
CNKSR2	ENSG00000149970	Connector enhancer of kinase suppressor of Ras 2	Q8WXI2
CNTF	ENSG00000242689	Ciliary neurotrophic factor	P26441
CNTNAP5	ENSG00000155052	Contactin associated protein like 5	Q8WYK1
CRTAM	ENSG00000109943	Cytotoxic and regulatory T cell molecule	O95727
CSMD3	ENSG00000164796	CUB and Sushi multiple domains 3	Q7Z407
CTXN1	ENSG00000178531	Cortexin 1	P60606
DLG4	ENSG00000132535	Discs large MAGUK scaffold protein 4	P78352
DLGAP1	ENSG00000170579	DLG associated protein 1	O14490
DPYSL5	ENSG00000157851	Dihydropyrimidinase like 5	Q9BPU6
DRP2	ENSG00000102385	Dystrophin related protein 2	Q13474
EN2	ENSG00000164778	Engrailed homeobox 2	P19622
FAIM2	ENSG00000135472	Fas apoptotic inhibitory molecule 2	Q9BWQ8
FAM163B	ENSG00000196990	Family with sequence similarity 163 member B	P0C2L3
FNTB	ENSG00000257365	Farnesyltransferase, CAAX box, beta	P49356
FOCAD	ENSG00000188352	Focadhesin	Q5VW36
GAD2	ENSG00000136750	Glutamate decarboxylase 2	Q05329
GHRH	ENSG00000118702	Growth hormone releasing hormone	P01286
GNG7	ENSG00000176533	G protein subunit gamma 7	O60262
GPR88	ENSG00000181656	G protein-coupled receptor 88	Q9GZN0
GRIN2A	ENSG00000183454	Glutamate ionotropic receptor NMDA type subunit 2A	Q12879
GRM1	ENSG00000152822	Glutamate metabotropic receptor 1	Q13255
HCRTR2	ENSG00000137252	Hypocretin receptor 2	O43614
HEPN1	ENSG00000221932	Hepatocellular carcinoma, down-regulated 1	Q6WQI6
HS3ST4	ENSG00000182601	Heparan sulfate-glucosamine 3-sulfotransferase 4	Q9Y661
HTR5A	ENSG00000157219	5-hydroxytryptamine receptor 5A	P47898
KCNA2	ENSG00000177301	Potassium voltage-gated channel subfamily A member 2	P16389
KCNQ2	ENSG00000075043	Potassium voltage-gated channel subfamily Q member 2	O43526
KCNS2	ENSG00000156486	Potassium voltage-gated channel modifier subfamily S member 2	Q9ULS6

KCTD16	ENSG00000183775	Potassium channel tetramerization domain containing 16	Q68DU8
KIAA0319	ENSG00000137261	KIAA0319	Q5VV43
KIF1A	ENSG00000130294	Kinesin family member 1A	Q12756
LLGL1	ENSG00000131899	LLGL1, scribble cell polarity complex component	Q15334
MAG	ENSG00000105695	Myelin associated glycoprotein	P20916
MAST1	ENSG00000105613	Microtubule associated serine/threonine kinase 1	Q9Y2H9
MC3R	ENSG00000124089	Melanocortin 3 receptor	P41968
MTCL1	ENSG00000168502	Microtubule crosslinking factor 1	Q9Y4B5
MYT1L	ENSG00000186487	Myelin transcription factor 1 like	Q9UL68
NAP1L2	ENSG00000186462	Nucleosome assembly protein 1 like 2	Q9ULW6
NECAB1	ENSG00000123119	N-terminal EF-hand calcium binding protein 1	Q8N987
NEFM	ENSG00000104722	Neurofilament medium	P07197
NKAIN4	ENSG00000101198	Sodium/potassium transporting ATPase interacting 4	Q8IVV8
NRSN1	ENSG00000152954	Neurensin 1	Q8IZ57
NSG2	ENSG00000170091	Neuronal vesicle trafficking associated 2	Q9Y328
OPRM1	ENSG00000112038	Opioid receptor mu 1	P35372
OR14I1	ENSG00000189181	Olfactory receptor family 14 subfamily I member 1	A6ND48
PACSIN1	ENSG00000124507	Protein kinase C and casein kinase substrate in neurons 1	Q9BY11
PCDHA5	ENSG00000204965	Protocadherin alpha 5	Q9Y5H7
PCDHGA10	ENSG00000253846	Protocadherin gamma subfamily A, 10	Q9Y5H3
PGM2L1	ENSG00000165434	Phosphoglucomutase 2 like 1	Q6PCE3
PIANP	ENSG00000139200	PILR alpha associated neural protein	Q8IYJ0
PLEKHB1	ENSG00000021300	Pleckstrin homology domain containing B1	Q9UF11
PNMA8C	ENSG00000277531	PNMA family member 8C	A0A1B0GUJ8
PPP2R2B	ENSG00000156475	Protein phosphatase 2 regulatory subunit Bbeta	Q00005
PTPRR	ENSG00000153233	Protein tyrosine phosphatase, receptor type R	Q15256
RNF112	ENSG00000128482	Ring finger protein 112	Q9ULX5
SCG3	ENSG00000104112	Secretogranin III	Q8WXD2
SCN1A	ENSG00000144285	Sodium voltage-gated channel alpha subunit 1	P35498
SEZ6L	ENSG00000100095	Seizure related 6 homolog like	Q9BYH1
SLAIN1	ENSG00000139737	SLAIN motif family member 1	Q8ND83
SLC24A2	ENSG00000155886	Solute carrier family 24 member 2	Q9UI40
SLC6A7	ENSG00000011083	Solute carrier family 6 member 7	Q99884
SLC8A2	ENSG00000118160	Solute carrier family 8 member A2	Q9UPR5
SNX32	ENSG00000172803	Sorting nexin 32	Q86XE0
SOX2	ENSG00000181449	SRY-box 2	P48431
ST8SIA3	ENSG00000177511	ST8 alpha-N-acetyl-neuraminide alpha-2,8-sialyltransferase 3	O43173
STRC	ENSG00000242866	Stereocilin	Q7RTU9
SYNGR3	ENSG00000127561	Synaptogyrin 3	O43761
TFAP2D	ENSG00000008197	Transcription factor AP-2 delta	Q7Z6R9
TIAM1	ENSG00000156299	T cell lymphoma invasion and metastasis 1	Q13009
TMEFF1	ENSG00000241697	Transmembrane protein with EGF like and two follistatin like domains 1	Q8IYR6
TMEM145	ENSG00000167619	Transmembrane protein 145	Q8NBT3
TMEM178A	ENSG00000152154	Transmembrane protein 178A	Q8NBL3
TMEM266	ENSG00000169758	Transmembrane protein 266	Q2M3C6
TMOD2	ENSG00000128872	Tropomodulin 2	Q9NZR1
TSPAN7	ENSG00000156298	Tetraspanin 7	P41732
TTYH2	ENSG00000141540	Tweety family member 2	Q9BSA4
TUNAR	ENSG00000250366	TCL1 upstream neural differentiation-associated RNA	
UGT8	ENSG00000174607	UDP glycosyltransferase 8	Q16880
ZFYVE28	ENSG00000159733	Zinc finger FYVE-type containing 28	Q9HCC9
ZIC5	ENSG00000139800	Zic family member 5	Q96T25
ACBD7	ENSG00000176244	Acyl-CoA binding domain containing 7	Q8N6N7

ANKRD34B	ENSG00000189127	Ankyrin repeat domain 34B	A5PLL1
APBA2	ENSG00000034053	Amyloid beta precursor protein binding family A member 2	Q99767
ARHGAP22	ENSG00000128805	Rho GTPase activating protein 22	Q7Z5H3
ARHGEF33	ENSG00000214694	Rho guanine nucleotide exchange factor 33	A8MVX0
BARHL2	ENSG00000143032	BarH like homeobox 2	Q9NY43
C17orf51	ENSG00000212719	Chromosome 17 open reading frame 51	A8MQB3
C2orf80	ENSG00000188674	Chromosome 2 open reading frame 80	Q0P641
CBLN2	ENSG00000141668	Cerebellin 2 precursor	Q8IUK8
CDH9	ENSG00000113100	Cadherin 9	Q9ULB4
CHRM5	ENSG00000184984	Cholinergic receptor muscarinic 5	P08912
CNP	ENSG00000173786	2',3'-cyclic nucleotide 3' phosphodiesterase	P09543
DCLK1	ENSG00000133083	Doublecortin like kinase 1	O15075
DCLK2	ENSG00000170390	Doublecortin like kinase 2	Q8N568
DLEU7	ENSG00000186047	Deleted in lymphocytic leukemia, 7	Q6UYE1
DLGAP3	ENSG00000116544	DLG associated protein 3	O95886
EGR4	ENSG00000135625	Early growth response 4	Q05215
ENHO	ENSG00000168913	Energy homeostasis associated	Q6UWT2
ENO2	ENSG00000111674	Enolase 2	P09104
EPHB1	ENSG00000154928	EPH receptor B1	P54762
ERC2	ENSG00000187672	ELKS/RAB6-interacting/CAST family member 2	O15083
FEZ1	ENSG00000149557	Fasciculation and elongation protein zeta 1	Q99689
FGF5	ENSG00000138675	Fibroblast growth factor 5	P12034
FOXB1	ENSG00000171956	Forkhead box B1	Q99853
GABRA2	ENSG00000151834	Gamma-aminobutyric acid type A receptor alpha2 subunit	P47869
GABRA4	ENSG00000109158	Gamma-aminobutyric acid type A receptor alpha4 subunit	P48169
GDI1	ENSG00000203879	GDP dissociation inhibitor 1	P31150
GPR37	ENSG00000170775	G protein-coupled receptor 37	O15354
GPR45	ENSG00000135973	G protein-coupled receptor 45	Q9Y5Y3
GPRIN1	ENSG00000169258	G protein regulated inducer of neurite outgrowth 1	Q7Z2K8
GRIN3A	ENSG00000198785	Glutamate ionotropic receptor NMDA type subunit 3A	Q8TCU5
HMSD	ENSG00000221887	Histocompatibility minor serpin domain containing	A8MTL9, P0C7T4
INA	ENSG00000148798	Internexin neuronal intermediate filament protein alpha	Q16352
IQCJ	ENSG00000214216	IQ motif containing J	Q1A5X6
KCNG2	ENSG00000178342	Potassium voltage-gated channel modifier subfamily G member 2	Q9UJ96
KCNK12	ENSG00000184261	Potassium two pore domain channel subfamily K member 12	Q9HB15
KIF5C	ENSG00000168280	Kinesin family member 5C	O60282
KLC1	ENSG00000126214	Kinesin light chain 1	Q07866
KLHL3	ENSG00000146021	Kelch like family member 3	Q9UH77
LGI1	ENSG00000108231	Leucine rich glioma inactivated 1	O95970
LINC00672	ENSG00000263874	Long intergenic non-protein coding RNA 672	
LIX1	ENSG00000145721	Limb and CNS expressed 1	Q8N485
LMTK3	ENSG00000142235	Lemur tyrosine kinase 3	Q96Q04
LRRC7	ENSG00000033122	Leucine rich repeat containing 7	Q96NW7
MAP1B	ENSG00000131711	Microtubule associated protein 1B	P46821
MMP24	ENSG00000125966	Matrix metallopeptidase 24	Q9Y5R2
MTURN	ENSG00000180354	Maturin, neural progenitor differentiation regulator homolog	Q8N3F0
NRIP2	ENSG00000053702	Nuclear receptor interacting protein 2	Q9BQI9
OLIG1	ENSG00000184221	Oligodendrocyte transcription factor 1	Q8TAK6
OLIG2	ENSG00000205927	Oligodendrocyte transcription factor 2	Q13516
OR9A2	ENSG00000179468	Olfactory receptor family 9 subfamily A member 2	Q8NGT5
PCDH19	ENSG00000165194	Protocadherin 19	Q8TAB3
PDE1B	ENSG00000123360	Phosphodiesterase 1B	Q01064
POU4F1	ENSG00000152192	POU class 4 homeobox 1	Q01851

RFPL1	ENSG00000128250	Ret finger protein like 1	O75677
RGS20	ENSG00000147509	Regulator of G protein signaling 20	O76081
RGS4	ENSG00000117152	Regulator of G protein signaling 4	P49798
RUNX1T1	ENSG00000079102	RUNX1 translocation partner 1	Q06455
S100B	ENSG00000160307	S100 calcium binding protein B	P04271
SHF	ENSG00000138606	Src homology 2 domain containing F	Q7M4L6
SHISA8	ENSG00000234965	Shisa family member 8	B8ZZ34
SLC26A5	ENSG00000170615	Solute carrier family 26 member 5	P58743
SLC44A1	ENSG00000070214	Solute carrier family 44 member 1	Q8WWI5
SLC6A17	ENSG00000197106	Solute carrier family 6 member 17	Q9H1V8
SLC6A5	ENSG00000165970	Solute carrier family 6 member 5	Q9Y345
SPTBN4	ENSG00000160460	Spectrin beta, non-erythrocytic 4	Q9H254
SSTR4	ENSG00000132671	Somatostatin receptor 4	P31391
STMN2	ENSG00000104435	Stathmin 2	Q93045
STMN3	ENSG00000197457	Stathmin 3	Q9NZ72
SVOP	ENSG00000166111	SV2 related protein	Q8N4V2
SYNJ2	ENSG00000078269	Synaptojanin 2	O15056
SYT5	ENSG00000129990	Synaptotagmin 5	O00445
TMEM155	ENSG00000164112	Transmembrane protein 155	Q4W5P6
TUBB2B	ENSG00000137285	Tubulin beta 2B class IIb	Q9BVA1
TUBB3	ENSG00000258947	Tubulin beta 3 class III	Q13509

Table S2. Potential Brain-Specific BDEV Markers.

The table enumerates potential brain-specific markers for BDEV as presented in Fig. 2C. The data was collated from the ExoCarta and EVpedia databases.

Gene description	Gene	Ensembl	Overlap with ExoCarta	EVpedia Reference No.
Adenylate cyclase 1	ADCY1	ENSG00000164742	O	5
Amyloid beta precursor like protein 1	APLP1	ENSG00000105290	O	8
BTB domain containing 17	BTBD17	ENSG00000204347	O	2
Calcium voltage-gated channel subunit alpha1 E	CACNA1E	ENSG00000198216	O	9
Calcium voltage-gated channel auxiliary subunit gamma 2	CACNG2	ENSG00000166862	O	0
CaM kinase like vesicle associated	CAMKV	ENSG00000164076	O	10
Cyclin dependent kinase 5 regulatory subunit 2	CDK5R2	ENSG00000171450	O	1
Connector enhancer of kinase suppressor of Ras 2	CNKSR2	ENSG00000149970	O	5
2',3'-cyclic nucleotide 3' phosphodiesterase	CNP	ENSG00000173786	O	242
Contactin associated protein like 4	CNTNAP4	ENSG00000152910	O	6
Copine 6	CPNE6	ENSG00000100884	O	11
Chondroitin sulfate proteoglycan 5	CSPG5	ENSG00000114646	O	6
Doublecortin like kinase 1	DCLK1	ENSG00000133083	O	11
Dendrin	DDN	ENSG00000181418	O	2
DIRAS family GTPase 2	DIRAS2	ENSG00000165023	O	8
Gamma-aminobutyric acid type A receptor beta2 subunit	GABRB2	ENSG00000145864	O	3
Glutamate decarboxylase 1	GAD1	ENSG00000128683	O	1
G protein subunit alpha o1	GNAO1	ENSG00000087258	O	73
Glutamate ionotropic receptor AMPA type subunit 2	GRIA2	ENSG00000120251	O	4
Glutamate metabotropic receptor 3	GRM3	ENSG00000198822	O	1
Hippocalcin	HPCA	ENSG00000121905	O	13
Interleukin 1 receptor accessory protein like 1	IL1RAPL1	ENSG00000169306	O	7
Junctophilin 3	JPH3	ENSG00000154118	O	3
Leucine rich repeat and Ig domain containing 1	LINGO1	ENSG00000169783	O	2
Microtubule associated protein 1B	MAP1B	ENSG00000131711	O	66
Myelin basic protein	MBP	ENSG00000197971	O	28
Multiple EGF like domains 10	MEGF10	ENSG00000145794	O	11
Purinergic receptor P2Y12	P2RY12	ENSG00000169313	O	1
Proteolipid protein 1	PLP1	ENSG00000123560	O	19
Protein kinase C gamma	PRKCG	ENSG00000126583	O	1
SH3 domain containing GRB2 like 2, endophilin A1	SH3GL2	ENSG00000107295	O	20
Solute carrier family 12 member 5	SLC12A5	ENSG00000124140	O	3
Solute carrier family 1 member 3	SLC1A3	ENSG00000079215	O	41
Solute carrier family 24 member 2	SLC24A2	ENSG00000155886	O	1
Syntaxin binding protein 1	STXBP1	ENSG00000136854	O	84

Table S3. GO term enrichment analysis in proteins profiles of EVs.

This table enumerates the GO term enrichment analysis results as presented in Fig. 6A and B. The proteomics data was collected from APLP1+ EVs and brain organoid-derived EVs. The cutoff for statistical significance was set at a *Adj. p-value* of 0.05 or lower.

Only APLP1+ EV_GO terms

ID	Description	Gene Ratio	Bg Ratio	*p*-value	*p*.adjust	*q*-value	Gene ID	Count
GO:0098883	synapse pruning	3/96	22/29151	5.0931E-05	0.002146377	0.001877681	C1QA/C1QB/C1QC	3
GO:0006956	complement activation	13/96	101/29151	1.48379E-17	2.6263E-14	2.29753E-14	CD5L/FCN3/C1QA/C1QB/C1QC/SERPING1/CFH/C1S/MBL2/C4BPB/MASP1/FCN2/COLEC11	13
GO:0006959	humoral immune response	16/96	389/29151	1.8654E-13	1.65088E-10	1.44421E-10	CD5L/FCN3/F2/C1QA/C1QB/C1QC/PPBP/PF4/SERPING1/CFH/C1S/MBL2/C4BPB/MASP1/FCN2/COLEC11	16
GO:0007596	blood coagulation	16/96	423/29151	6.6915E-13	3.62058E-10	3.16734E-10	MYL12A/F2/PLG/KNG1/SLC4A1/PF4/VTN/VWF/SERPING1/PROS1/GP1BA/C4BPB/PIK3CB/CAV1/FERMT3/TLN1	16
GO:0050817	coagulation	16/96	429/29151	8.28617E-13	3.62058E-10	3.16734E-10	MYL12A/F2/PLG/KNG1/SLC4A1/PF4/VTN/VWF/SERPING1/PROS1/GP1BA/C4BPB/PIK3CB/CAV1/FERMT3/TLN1	16
GO:0007599	hemostasis	16/96	435/29151	1.02276E-12	3.62058E-10	3.16734E-10	MYL12A/F2/PLG/KNG1/SLC4A1/PF4/VTN/VWF/SERPING1/PROS1/GP1BA/C4BPB/PIK3CB/CAV1/FERMT3/TLN1	16
GO:0001867	complement activation, lectin pathway	6/96	17/29151	1.30815E-11	3.85905E-09	3.37595E-09	FCN3/SERPING1/MBL2/MASP1/FCN2/COLEC11	6
GO:0006958	complement activation, classical pathway	7/96	64/29151	1.79245E-09	4.53234E-07	3.96495E-07	C1QA/C1QB/C1QC/SERPING1/C1S/MBL2/C4BPB	7
GO:1903027	regulation of opsonization	5/96	18/29151	2.88702E-09	6.38753E-07	5.5879E-07	FCN3/MBL2/C4BPB/FCN2/COLEC11	5
GO:0042730	fibrinolysis	6/96	49/29151	1.35799E-08	2.67071E-06	2.33637E-06	F2/PLG/VTN/SERPING1/PROS1/GP1BA	6
GO:0030193	regulation of blood coagulation	8/96	134/29151	1.52421E-08	2.69785E-06	2.36011E-06	F2/PLG/KNG1/VTN/SERPING1/PROS1/GP1BA/CAV1	8
GO:1900046	regulation of hemostasis	8/96	136/29151	1.71226E-08	2.75518E-06	2.41027E-06	F2/PLG/KNG1/VTN/SERPING1/PROS1/GP1BA/CAV1	8
GO:0002455	humoral immune response mediated by circulating immunoglobulin	7/96	91/29151	2.1728E-08	2.76765E-06	2.42118E-06	C1QA/C1QB/C1QC/SERPING1/C1S/MBL2/C4BPB	7
GO:0030195	negative regulation of blood coagulation	7/96	91/29151	2.1728E-08	2.76765E-06	2.42118E-06	F2/PLG/KNG1/VTN/SERPING1/PROS1/GP1BA	7
GO:0050818	regulation of coagulation	8/96	141/29151	2.27231E-08	2.76765E-06	2.42118E-06	F2/PLG/KNG1/VTN/SERPING1/PROS1/GP1BA/CAV1	8
GO:1900047	negative regulation of hemostasis	7/96	92/29151	2.34546E-08	2.76765E-06	2.42118E-06	F2/PLG/KNG1/VTN/SERPING1/PROS1/GP1BA	7
GO:0050819	negative regulation of coagulation	7/96	95/29151	2.93481E-08	3.24664E-06	2.8402E-06	F2/PLG/KNG1/VTN/SERPING1/PROS1/GP1BA	7
GO:0008228	opsonization	5/96	28/29151	3.22649E-08	3.35934E-06	2.9388E-06	FCN3/MBL2/C4BPB/FCN2/COLEC11	5

GO:0002752	cell surface pattern recognition receptor signaling pathway	4/96	11/29151	3.58023E-08	3.52056E-06	3.07983E-06	FCN3/MBL2/FCN2/COLEC11	4
GO:0010951	negative regulation of endopeptidase activity	11/96	429/29151	1.77292E-07	1.65162E-05	1.44486E-05	KNG1/AMBP/AHSG/VTN/SERPING1/PROS1/ITIH2/ITIH1/SERPINB4/CSNK2A1/ITIH3	11
GO:1903028	positive regulation of opsonization	4/96	16/29151	1.94976E-07	1.72554E-05	1.50953E-05	FCN3/MBL2/FCN2/COLEC11	4
GO:0061045	negative regulation of wound healing	7/96	128/29151	2.29865E-07	1.93743E-05	1.69489E-05	F2/PLG/KNG1/VTN/SERPING1/PROS1/GP1BA	7
GO:0010466	negative regulation of peptidase activity	11/96	451/29151	2.91513E-07	2.34536E-05	2.05175E-05	KNG1/AMBP/AHSG/VTN/SERPING1/PROS1/ITIH2/ITIH1/SERPINB4/CSNK2A1/ITIH3	11
GO:0002181	cytoplasmic translation	9/96	275/29151	3.28903E-07	2.53112E-05	2.21426E-05	RPL7/RPS3/RPS27/RPL21/RPS16/RPS18/RPS4X/RPL23A/RPS26	9
GO:1903035	negative regulation of response to wounding	7/96	160/29151	1.04094E-06	7.57442E-05	6.62621E-05	F2/PLG/KNG1/VTN/SERPING1/PROS1/GP1BA	7
GO:0061041	regulation of wound healing	8/96	233/29151	1.06983E-06	7.57442E-05	6.62621E-05	F2/PLG/KNG1/VTN/SERPING1/PROS1/GP1BA/CAV1	8
GO:0006910	phagocytosis, recognition	5/96	57/29151	1.27475E-06	8.67807E-05	7.5917E-05	FCN3/MBL2/C4BPB/FCN2/COLEC11	5
GO:0030168	platelet activation	8/96	246/29151	1.6055E-06	0.000105249	9.20737E-05	MYL12A/F2/PF4/VWF/GP1BA/PIK3CB/FERMT3/TLN1	8
GO:0002218	activation of innate immune response	6/96	110/29151	1.77004E-06	0.000111892	9.78843E-05	FCN3/TOMM70/MBL2/MATR3/FCN2/COLEC11	6
GO:0030449	regulation of complement activation	4/96	33/29151	4.1996E-06	0.000256321	0.000224233	CD5L/SERPING1/CFH/C4BPB	4
GO:0034447	very-low-density lipoprotein particle clearance	3/96	11/29151	5.60236E-06	0.000330539	0.00028916	APOC1/APOC2/LRPAP1	3
GO:1903034	regulation of response to wounding	8/96	297/29151	6.44738E-06	0.000368125	0.000322041	F2/PLG/KNG1/VTN/SERPING1/PROS1/GP1BA/CAV1	8
GO:0045088	regulation of innate immune response	9/96	440/29151	1.52386E-05	0.000842885	0.000737367	FCN3/TOMM70/SERPING1/CFH/MBL2/MATR3/SERPINB4/FCN2/COLEC11	9
GO:0033344	cholesterol efflux	5/96	101/29151	2.15115E-05	0.0011538	0.001009361	APOC1/APOC2/PON1/CAV1/ADIPOQ	5
GO:0010985	negative regulation of lipoprotein particle clearance	3/96	18/29151	2.72461E-05	0.0014184	0.001240836	APOC1/APOC2/LRPAP1	3
GO:0050764	regulation of phagocytosis	6/96	185/29151	3.47803E-05	0.001748948	0.001530005	FCN3/AHSG/MBL2/FCN2/ADIPOQ/COLEC11	6
GO:0002220	innate immune response activating cell surface receptor signaling pathway	4/96	56/29151	3.55718E-05	0.001748948	0.001530005	FCN3/MBL2/FCN2/COLEC11	4
GO:0045806	negative regulation of endocytosis	5/96	113/29151	3.69525E-05	0.001767729	0.001546434	APOC1/APOC2/LRPAP1/CAV1/ADIPOQ	5
GO:0032371	regulation of sterol transport	5/96	117/29151	4.36544E-05	0.001935805	0.00169347	APOC1/APOC2/PON1/CAV1/ADIPOQ	5
GO:0032374	regulation of cholesterol transport	5/96	117/29151	4.36544E-05	0.001935805	0.00169347	APOC1/APOC2/PON1/CAV1/ADIPOQ	5

GO:0002758	innate immune response-activating signal transduction	4/96	59/29151	4.3747E-05	0.001935805	0.00169347	FCN3/MBL2/FCN2/COLEC11	4
GO:0051918	negative regulation of fibrinolysis	3/96	22/29151	5.0931E-05	0.002146377	0.001877681	F2/PLG/VTN	3
GO:0016064	immunoglobulin mediated immune response	7/96	299/29151	6.08649E-05	0.002505367	0.002191731	C1QA/C1QB/C1QC/SERPING1/C1S/MBL2/C4BPB	7
GO:0048261	negative regulation of receptor-mediated endocytosis	4/96	65/29151	6.41022E-05	0.002578656	0.002255845	APOC1/APOC2/LRPAP1/ADIPOQ	4
GO:0019724	B cell mediated immunity	7/96	305/29151	6.89345E-05	0.002711423	0.002371991	C1QA/C1QB/C1QC/SERPING1/C1S/MBL2/C4BPB	7
GO:0050766	positive regulation of phagocytosis	5/96	130/29151	7.211E-05	0.002774667	0.002427318	FCN3/AHSG/MBL2/FCN2/COLEC11	5
GO:0048259	regulation of receptor-mediated endocytosis	6/96	224/29151	0.000100323	0.00372436	0.003258123	APOC1/APOC2/VTN/LRPAP1/PIK3CB/ADIPOQ	6
GO:0002920	regulation of humoral immune response	4/96	73/29151	0.000101	0.00372436	0.003258123	CD5L/SERPING1/CFH/C4BPB	4
GO:0034381	plasma lipoprotein particle clearance	4/96	75/29151	0.000112221	0.00405368	0.003546216	APOC1/APOC2/LRPAP1/ADIPOQ	4
GO:0070527	platelet aggregation	5/96	145/29151	0.000120723	0.004273586	0.003738594	MYL12A/GP1BA/PIK3CB/FERMT3/TLN1	5
GO:0051917	regulation of fibrinolysis	3/96	33/29151	0.000175763	0.006100004	0.00533637	F2/PLG/VTN	3
GO:0045089	positive regulation of innate immune response	6/96	255/29151	0.000202912	0.006906817	0.006042182	FCN3/TOMM70/MBL2/MATR3/FCN2/COLEC11	6
GO:0010984	regulation of lipoprotein particle clearance	3/96	36/29151	0.000228372	0.007626755	0.006671993	APOC1/APOC2/LRPAP1	3
GO:0045649	regulation of macrophage differentiation	3/96	37/29151	0.00024793	0.008126599	0.007109263	C1QC/PF4/ADIPOQ	3
GO:0051873	killing by host of symbiont cells	3/96	38/29151	0.00026854	0.008642108	0.007560238	F2/PF4/MBL2	3
GO:0030301	cholesterol transport	5/96	186/29151	0.000383016	0.012106029	0.010590525	APOC1/APOC2/PON1/CAV1/ADIPOQ	5
GO:0034109	homotypic cell-cell adhesion	5/96	190/29151	0.000422119	0.013107898	0.011466975	MYL12A/GP1BA/PIK3CB/FERMT3/TLN1	5
GO:0043654	recognition of apoptotic cell	2/96	10/29151	0.000474727	0.014487369	0.012673756	FCN3/FCN2	2
GO:0051702	biological process involved in interaction with symbiont	5/96	197/29151	0.000497711	0.014931323	0.013062133	F2/PLG/PF4/MBL2/STOM	5
GO:0030194	positive regulation of blood coagulation	3/96	49/29151	0.000571311	0.016012343	0.014007824	F2/PLG/VTN	3
GO:0150146	cell junction disassembly	3/96	49/29151	0.000571311	0.016012343	0.014007824	C1QA/C1QB/C1QC	3

GO:1900048	positive regulation of hemostasis	3/96	49/29151	0.000571311	0.016012343	0.014007824	F2/PLG/VTN	3
GO:0034382	chylomicron remnant clearance	2/96	11/29151	0.000578977	0.016012343	0.014007824	APOC1/APOC2	2
GO:0071830	triglyceride-rich lipoprotein particle clearance	2/96	11/29151	0.000578977	0.016012343	0.014007824	APOC1/APOC2	2
GO:1905952	regulation of lipid localization	6/96	313/29151	0.000602464	0.016405571	0.014351826	APOC1/APOC2/PON1/APOC4/CAV1/ADIPOQ	6
GO:0009205	purine ribonucleoside triphosphate metabolic process	7/96	439/29151	0.000631075	0.016924284	0.014805603	SLC4A1/MYH8/NT5E/ATP5F1C/VCP/RAB23/MYH4	7
GO:0002833	positive regulation of response to biotic stimulus	6/96	320/29151	0.000676155	0.017862589	0.015626446	FCN3/TOMM70/MBL2/MATR3/FCN2/COLEC11	6
GO:0030100	regulation of endocytosis	7/96	447/29151	0.000701672	0.018193945	0.01591632	APOC1/APOC2/VTN/LRPAP1/PIK3CB/CAV1/ADIPOQ	7
GO:0009144	purine nucleoside triphosphate metabolic process	7/96	448/29151	0.00071092	0.018193945	0.01591632	SLC4A1/MYH8/NT5E/ATP5F1C/VCP/RAB23/MYH4	7
GO:0036296	response to increased oxygen levels	3/96	53/29151	0.000719535	0.018193945	0.01591632	ATP6V1G1/COL1A1/CAV1	3
GO:0006909	phagocytosis	7/96	452/29151	0.000748884	0.018649449	0.016314802	FCN3/AHSG/MBL2/C4BPB/FCN2/ADIPOQ/COLEC11	7
GO:0009199	ribonucleoside triphosphate metabolic process	7/96	453/29151	0.000758622	0.018649449	0.016314802	SLC4A1/MYH8/NT5E/ATP5F1C/VCP/RAB23/MYH4	7
GO:0015918	sterol transport	5/96	217/29151	0.000770206	0.018674858	0.01633703	APOC1/APOC2/PON1/CAV1/ADIPOQ	5
GO:0050820	positive regulation of coagulation	3/96	55/29151	0.000801988	0.019182683	0.016781283	F2/PLG/VTN	3
GO:0010875	positive regulation of cholesterol efflux	3/96	57/29151	0.000890215	0.021009072	0.018379033	PON1/CAV1/ADIPOQ	3
GO:0060696	regulation of phospholipid catabolic process	2/96	14/29151	0.000951794	0.022166788	0.01939182	APOC1/APOC2	2
GO:0097006	regulation of plasma lipoprotein particle levels	4/96	134/29151	0.001022502	0.023504263	0.020561862	APOC1/APOC2/LRPAP1/ADIPOQ	4
GO:0009141	nucleoside triphosphate metabolic process	7/96	480/29151	0.001061839	0.024095582	0.021079156	SLC4A1/MYH8/NT5E/ATP5F1C/VCP/RAB23/MYH4	7
GO:0045650	negative regulation of macrophage differentiation	2/96	15/29151	0.00109587	0.024553038	0.021479345	C1QC/ADIPOQ	2
GO:0030212	hyaluronan metabolic process	3/96	62/29151	0.001137049	0.025157203	0.022007878	ITIH2/ITIH1/ITIH3	3
GO:0006898	receptor-mediated endocytosis	7/96	497/29151	0.00129701	0.028342072	0.024794045	APOC1/APOC2/VTN/LRPAP1/PIK3CB/CAV1/ADIPOQ	7
GO:1903659	regulation of complement-	2/96	17/29151	0.001413336	0.030507379	0.026688287	CD5L/CFH	2

	dependent cytotoxicity							
GO:0050805	negative regulation of synaptic transmission	4/96	147/29151	0.001439288	0.030693246	0.026850886	RAP1A/ADIPOQ/NLGN4X/SORCS2	4
GO:0032368	regulation of lipid transport	5/96	254/29151	0.001549025	0.032279051	0.028238171	APOC1/APOC2/PON1/CAV1/ADIPOQ	5
GO:0030853	negative regulation of granulocyte differentiation	2/96	18/29151	0.001586597	0.032279051	0.028238171	C1QC/ADIPOQ	2
GO:0032372	negative regulation of sterol transport	2/96	18/29151	0.001586597	0.032279051	0.028238171	APOC1/APOC2	2
GO:0032375	negative regulation of cholesterol transport	2/96	18/29151	0.001586597	0.032279051	0.028238171	APOC1/APOC2	2
GO:0045916	negative regulation of complement activation	2/96	19/29151	0.001769459	0.035590247	0.031134852	SERPING1/C4BPB	2
GO:0010874	regulation of cholesterol efflux	3/96	73/29151	0.001821689	0.036114258	0.031593264	PON1/CAV1/ADIPOQ	3
GO:0046034	ATP metabolic process	6/96	389/29151	0.001836318	0.036114258	0.031593264	SLC4A1/MYH8/NT5E/ATP5F1C/VCP/MYH4	6
GO:0019217	regulation of fatty acid metabolic process	4/96	167/29151	0.002292002	0.043789162	0.038307379	APOC1/APOC2/CAV1/ADIPOQ	4
GO:0019730	antimicrobial humoral response	4/96	168/29151	0.002342056	0.043789162	0.038307379	F2/PPBP/PF4/COLEC11	4
GO:0032373	positive regulation of sterol transport	3/96	80/29151	0.002366798	0.043789162	0.038307379	PON1/CAV1/ADIPOQ	3
GO:0032376	positive regulation of cholesterol transport	3/96	80/29151	0.002366798	0.043789162	0.038307379	PON1/CAV1/ADIPOQ	3
GO:0071712	ER-associated misfolded protein catabolic process	2/96	22/29151	0.002375005	0.043789162	0.038307379	VCP/UGGT1	2
GO:0097278	complement-dependent cytotoxicity	2/96	22/29151	0.002375005	0.043789162	0.038307379	CD5L/CFH	2
GO:0033034	positive regulation of myeloid cell apoptotic process	2/96	23/29151	0.002595632	0.046880284	0.041011536	PIK3CB/ADIPOQ	2
GO:0033700	phospholipid efflux	2/96	23/29151	0.002595632	0.046880284	0.041011536	APOC1/APOC2	2
GO:0008360	regulation of cell shape	5/96	289/29151	0.002712816	0.048501858	0.042430112	MYL12A/WDR1/F2/CYFIP1/MYH14	5

Only EV from organoid_GO terms

ID	Description	Gene Ratio	Bg Ratio	*p*-value	*p*.adjust	*q*-value	Gene ID	Count
GO:0051580	regulation of neurotransmitter uptake	4/231	40/29151	0.000280657	0.021451923	0.019324842	FLOT1/ITGB1/GFAP/ATP1A2	4
GO:0098810	neurotransmitter reuptake	5/231	69/29151	0.000222569	0.017785269	0.016021757	ITGB1/SLC1A2/SLC6A11/ATP1A2/PARK7	5

GO:0051933	amino acid neurotransmitter reuptake	3/231	14/29151	0.000167612	0.016370067	0.014746881	ITGB1/SLC6A11/ATP1A2	3
GO:0001504	neurotransmitter uptake	7/231	91/29151	8.24116E-06	0.003219545	0.002900309	FLOT1/ITGB1/GFAP/SLC1A2/SLC6A11/ATP1A2/PARK7	7
GO:0062207	regulation of pattern recognition receptor signaling pathway	7/231	190/29151	0.000834384	0.048093324	0.043324595	DDX3X/FLOT1/HMGB1/C1QBP/BPIFB1/RTN4/UBQLN1	7
GO:0034121	regulation of toll-like receptor signaling pathway	6/231	136/29151	0.000774282	0.045372953	0.040873964	DDX3X/FLOT1/HMGB1/BPIFB1/RTN4/UBQLN1	6
GO:0042255	ribosome assembly	5/231	90/29151	0.000760878	0.045343166	0.040847131	DDX3X/XRCC5/PRKDC/RPL6/C1QBP	5
GO:0006541	glutamine metabolic process	4/231	51/29151	0.000716936	0.043461171	0.039151747	GLUD1/ASL/CPS1/GMPS	4
GO:0032392	DNA geometric change	7/231	184/29151	0.00069105	0.042626874	0.038400175	DDX3X/HMGB1/XRCC6/XRCC5/RECQL/SSBP1/G3BP1	7
GO:0010951	negative regulation of endopeptidase activity	11/231	429/29151	0.000676119	0.042450624	0.038241401	DDX3X/CST3/SMR3B/SLPI/CSTB/CST2/CST5/PEBP1/SERPINH1/WFDC2/PARK7	11
GO:0070199	establishment of protein localization to chromosome	4/231	49/29151	0.000615411	0.039341515	0.035440578	TCP1/CCT6A/CCT3/CCT7	4
GO:1901607	alpha-amino acid biosynthetic process	6/231	128/29151	0.000563067	0.036661944	0.033026701	GLUD1/ASL/CPS1/CBS/BHMT/PARK7	6
GO:0000041	transition metal ion transport	7/231	174/29151	0.000496092	0.032945783	0.02967902	TFRC/FTL/FTH1/TCN1/B2M/CLTC/TTYH1	7
GO:0032206	positive regulation of telomere maintenance	6/231	125/29151	0.000496623	0.032945783	0.02967902	XRCC5/TCP1/CTNNB1/CCT6A/CCT3/CCT7	6
GO:0031099	regeneration	10/231	343/29151	0.000441906	0.030465498	0.027444669	CSPG5/SPP1/GFAP/GAP43/PTN/MDK/ATIC/CD81/CPB2/PTGFRN	10
GO:1900182	positive regulation of protein localization to nucleus	7/231	165/29151	0.000360715	0.025365459	0.022850328	TFRC/HSP90AA1/TCP1/CCT6A/CCT3/PARK7/CCT7	7
GO:0042254	ribosome biogenesis	12/231	455/29151	0.000302078	0.022548592	0.02031277	NOP56/DDX3X/XRCC5/RPS8/RPL7A/RPS6/PRKDC/RPL6/C1QBP/PA2G4/NOP58/UTP18	12
GO:0050821	protein stabilization	11/231	391/29151	0.000312397	0.022548592	0.02031277	FLOT1/HSP90AA1/LAMP2/TCP1/PPIB/CALR/CCT6A/CCT3/PARK7/CCT7/RTN4	11
GO:0034976	response to endoplasmic reticulum stress	12/231	457/29151	0.000314244	0.022548592	0.02031277	DDX3X/FLOT1/ERLIN2/AIFM1/PDIA4/CALR/CANX/MARCKS/PARK7/TMX1/UBQLN1/HYOU1	12
GO:0016054	organic acid catabolic process	12/231	442/29151	0.000232337	0.018153238	0.016353241	GCAT/GLUD1/PCCB/ACAA1/FAH/CBS/ACAA2/ACADVL/BLMH/DECR1/GCDH/ALDH8A1	12
GO:1904872	regulation of telomerase RNA localization to Cajal body	4/231	36/29151	0.000185438	0.01703137	0.015342612	TCP1/CCT6A/CCT3/CCT7	4

GO:0046395	carboxylic acid catabolic process	12/231	433/29151	0.000192535	0.01703137	0.015342612	GCAT/GLUD1/PCCB/ACAA1/FAH/CBS/ACAA2/ACADVL/BLMH/DECR1/GCDH/ALDH8A1	12
GO:0090670	RNA localization to Cajal body	4/231	37/29151	0.00020663	0.01703137	0.015342612	TCP1/CCT6A/CCT3/CCT7	4
GO:0090671	telomerase RNA localization to Cajal body	4/231	37/29151	0.00020663	0.01703137	0.015342612	TCP1/CCT6A/CCT3/CCT7	4
GO:0090672	telomerase RNA localization	4/231	37/29151	0.00020663	0.01703137	0.015342612	TCP1/CCT6A/CCT3/CCT7	4
GO:0090685	RNA localization to nucleus	4/231	37/29151	0.00020663	0.01703137	0.015342612	TCP1/CCT6A/CCT3/CCT7	4
GO:0075522	IRES-dependent viral translational initiation	3/231	15/29151	0.00020829	0.01703137	0.015342612	EIF3D/PTBP1/PCBP2	3
GO:0070200	establishment of protein localization to telomere	4/231	35/29151	0.00016586	0.016370067	0.014746881	TCP1/CCT6A/CCT3/CCT7	4
GO:0006826	iron ion transport	6/231	99/29151	0.000140682	0.014882811	0.013407095	TFRC/FTL/FTH1/B2M/CLTC/TTYH1	6
GO:2000573	positive regulation of DNA biosynthetic process	7/231	142/29151	0.000143918	0.014882811	0.013407095	HSP90AA1/XRCC5/TCP1/CTNNB1/CCT6A/CCT3/CCT7	7
GO:1904874	positive regulation of telomerase RNA localization to Cajal body	4/231	32/29151	0.000116051	0.013126794	0.011825197	TCP1/CCT6A/CCT3/CCT7	4
GO:2000278	regulation of DNA biosynthetic process	9/231	236/29151	0.00011947	0.013126794	0.011825197	HSP90AA1/HNRNPC/XRCC5/TCP1/CTNNB1/CCT6A/CCT3/HNRNPU/CCT7	9
GO:0032204	regulation of telomere maintenance	8/231	180/29151	9.99693E-05	0.011716406	0.010554657	HNRNPC/XRCC5/TCP1/CTNNB1/CCT6A/CCT3/HNRNPU/CCT7	8
GO:1904814	regulation of protein localization to chromosome, telomeric region	4/231	29/29151	7.80912E-05	0.009467879	0.008529085	TCP1/CCT6A/CCT3/CCT7	4
GO:0050667	homocysteine metabolic process	4/231	28/29151	6.77396E-05	0.008506159	0.007662725	CPS1/CBS/BLMH/BHMT	4
GO:1990173	protein localization to nucleoplasm	4/231	28/29151	6.77396E-05	0.008506159	0.007662725	TCP1/CCT6A/CCT3/CCT7	4
GO:0070198	protein localization to chromosome, telomeric region	5/231	52/29151	5.74095E-05	0.007900797	0.007117388	XRCC5/TCP1/CCT6A/CCT3/CCT7	5
GO:0048771	tissue remodeling	11/231	322/29151	5.75391E-05	0.007900797	0.007117388	CST3/TFRC/SPP1/GJA1/PTN/MDK/IGFBP5/CTNNB1/CBS/LEPR/RAB7A	11
GO:1904816	positive regulation of protein localization to chromosome, telomeric region	4/231	27/29151	5.84246E-05	0.007900797	0.007117388	TCP1/CCT6A/CCT3/CCT7	4
GO:1901605	alpha-amino acid metabolic process	12/231	374/29151	4.86976E-05	0.007444387	0.006706233	GCAT/GLUD1/ASL/FAH/CPS1/CBS/GMPS/BLMH/GCDH/BHMT/PARK7/ALDH8A1	12

GO:1904851	positive regulation of establishment of protein localization to telomere	4/231	23/29151	3.02212E-05	0.00681453	0.006138831	TCP1/CCT6A/CCT3/CCT7	4
GO:0070203	regulation of establishment of protein localization to telomere	4/231	24/29151	3.60405E-05	0.00681453	0.006138831	TCP1/CCT6A/CCT3/CCT7	4
GO:1904869	regulation of protein localization to Cajal body	4/231	24/29151	3.60405E-05	0.00681453	0.006138831	TCP1/CCT6A/CCT3/CCT7	4
GO:1904871	positive regulation of protein localization to Cajal body	4/231	24/29151	3.60405E-05	0.00681453	0.006138831	TCP1/CCT6A/CCT3/CCT7	4
GO:0046849	bone remodeling	8/231	159/29151	4.18091E-05	0.00681453	0.006138831	TFRC/SPP1/GJA1/PTN/MDK/CTNNB1/LEPR/RAB7A	8
GO:0019081	viral translation	4/231	25/29151	4.26393E-05	0.00681453	0.006138831	EIF3D/PTBP1/PCBP2/EIF3L	4
GO:0070202	regulation of establishment of protein localization to chromosome	4/231	25/29151	4.26393E-05	0.00681453	0.006138831	TCP1/CCT6A/CCT3/CCT7	4
GO:1903405	protein localization to nuclear body	4/231	25/29151	4.26393E-05	0.00681453	0.006138831	TCP1/CCT6A/CCT3/CCT7	4
GO:1904867	protein localization to Cajal body	4/231	25/29151	4.26393E-05	0.00681453	0.006138831	TCP1/CCT6A/CCT3/CCT7	4
GO:1904358	positive regulation of telomere maintenance via telomere lengthening	6/231	72/29151	2.34281E-05	0.006336391	0.005708101	XRCC5/TCP1/CTNNB1/CCT6A/CCT3/CCT7	6
GO:0032200	telomere organization	11/231	285/29151	1.89558E-05	0.005554061	0.005003344	HSP90AA1/HNRNPC/XRCC6/XRCC5/TCP1/CTNNB1/CCT6A/CCT3/PRKDC/HNRNPU/CCT7	11
GO:0032212	positive regulation of telomere maintenance via telomerase	6/231	68/29151	1.6851E-05	0.005386186	0.004852115	XRCC5/TCP1/CTNNB1/CCT6A/CCT3/CCT7	6
GO:0000723	telomere maintenance	11/231	270/29151	1.14714E-05	0.00403335	0.00363342	HSP90AA1/HNRNPC/XRCC6/XRCC5/TCP1/CTNNB1/CCT6A/CCT3/PRKDC/HNRNPU/CCT7	11
GO:0002181	cytoplasmic translation	12/231	275/29151	2.26196E-06	0.000994132	0.000895558	EIF3D/RPLP2/PABPC1/RPS2/RPS12/RPS8/RPL7A/RPS6/HNRNPU/RPL6/RPL18/EIF3L	12
GO:1904356	regulation of telomere maintenance via telomere lengthening	8/231	102/29151	1.59232E-06	0.000888702	0.000800582	HNRNPC/XRCC5/TCP1/CTNNB1/CCT6A/CCT3/HNRNPU/CCT7	8
GO:0010833	telomere maintenance via telomere lengthening	9/231	139/29151	1.76932E-06	0.000888702	0.000800582	HSP90AA1/HNRNPC/XRCC5/TCP1/CTNNB1/CCT6A/CCT3/HNRNPU/CCT7	9

GO:0061077	chaperone-mediated protein folding	9/231	128/29151	8.87464E-07	0.000624065	0.000562185	PDIA4/TCP1/PPIB/CCT6A/CCT3/HSPA2/HSPE1/TRAP1/CCT7	9
GO:0006457	protein folding	15/231	385/29151	5.11274E-07	0.000596772	0.000537599	HSP90AA1/PDIA4/TCP1/PPIB/CALR/CANX/STIP1/CCT6A/CCT3/HSPA2/HSPE1/B2M/TRAP1/CCT7/HYOU1	15
GO:0007004	telomere maintenance via telomerase	9/231	122/29151	5.91619E-07	0.000596772	0.000537599	HSP90AA1/HNRNPC/XRCC5/TCP1/CTNNB1/CCT6A/CCT3/HNRNPU/CCT7	9
GO:0032210	regulation of telomere maintenance via telomerase	8/231	91/29151	6.64944E-07	0.000596772	0.000537599	HNRNPC/XRCC5/TCP1/CTNNB1/CCT6A/CCT3/HNRNPU/CCT7	8
GO:0006278	RNA-templated DNA biosynthetic process	9/231	124/29151	6.78922E-07	0.000596772	0.000537599	HSP90AA1/HNRNPC/XRCC5/TCP1/CTNNB1/CCT6A/CCT3/HNRNPU/CCT7	9

intersection_GO terms

ID	Description	Gene Ratio	Bg Ratio	*p*-value	*p*.adjust	*q*-value	Gene ID	Count
GO:0014002	astrocyte development	5/255	81/29151	0.00073439	0.014767996	0.012497517	S100A8/S100A9/VIM/LAMB2/LRP1	5
GO:0021762	substantia nigra development	5/255	82/29151	0.000776568	0.0154387	0.013065105	FGF2/CALM1/HSPA5/YWHAE/BASP1	5
GO:0031103	axon regeneration	5/255	104/29151	0.00224115	0.034545298	0.029234194	APOD/FLNA/TNC/LAMB2/TNR	5
GO:1900221	regulation of amyloid-beta clearance	3/255	31/29151	0.00248111	0.036942146	0.031262543	APOE/CLU/LRP1	3
GO:0097242	amyloid-beta clearance	4/255	67/29151	0.002846532	0.040987712	0.034686131	C3/APOE/CLU/LRP1	4
GO:0031102	neuron projection regeneration	5/255	114/29151	0.003334789	0.044558436	0.037707881	APOD/FLNA/TNC/LAMB2/TNR	5
GO:0021782	glial cell development	7/255	228/29151	0.004082713	0.04944974	0.04184718	S100A8/S100A9/VIM/CLU/LAMB2/LRP1/PLEC	7
GO:0006959	humoral immune response	23/255	389/29151	7.40005E-13	2.58928E-09	2.19119E-09	IGLL5/C1R/CFB/A2M/C3/C5/JCHAIN/FGA/FGB/TF/LTF/HPX/C4BPA/HRG/GAPDH/S100A9/C4B/CLU/S100A7/PHB1/LYZ/MUC7/DMBT1	23
GO:0010466	negative regulation of peptidase activity	23/255	451/29151	1.50996E-11	2.64168E-08	2.23554E-08	SERPINC1/SERPINA1/SERPINA3/AGT/A2M/C3/C5/CST4/CST1/CSTA/LTF/HRG/GAPDH/ANXA2/C4B/SERPINB3/PRDX5/LCN1/SFN/LRP1/ITIH4/ECM1/SERPINB12	23
GO:0007596	blood coagulation	22/255	423/29151	2.91937E-11	2.69613E-08	2.28161E-08	SERPINC1/SERPINA1/APOE/FGA/FGB/FGG/APOH/FN1/F11/HRG/HSPB1/ANXA2/ANXA5/F5/GP1BB/FLNA/CD9/PRDX2/MYH9/PPIA/ACTG1/HBB	22
GO:0050817	coagulation	22/255	429/29151	3.82957E-11	2.69613E-08	2.28161E-08	SERPINC1/SERPINA1/APOE/FGA/FGB/FGG/APOH/FN1/F11/HRG/HSPB1/ANXA2/ANXA5/F5/GP1BB/FLNA/CD9/PRDX2/MYH9/PPIA/ACTG1/HBB	22
GO:0001895	retina homeostasis	14/255	144/29151	3.85271E-11	2.69613E-08	2.28161E-08	CST4/JCHAIN/PIGR/TF/LTF/HSPB1/PIP/AZGP1/LCN1/LYZ/ACTG1/PRDX1/PRR4/ZG16B	14

GO:0007599	hemostasis	22/255	435/29151	5.00085E-11	2.91633E-08	2.46796E-08	SERPINC1/SERPINA1/APOE/FGA/FGB/FGG/APOH/FN1/F11/HRG/HSPB1/ANXA2/ANXA5/F5/GP1BB/FLNA/CD9/PRDX2/MYH9/PPIA/ACTG1/HBB	22
GO:0072378	blood coagulation, fibrin clot formation	8/255	30/29151	1.52236E-10	7.60964E-08	6.43971E-08	SERPINC1/FGA/FGB/FGG/APOH/FN1/GP1BB/FLNA	8
GO:0006457	protein folding	20/255	385/29151	2.44265E-10	8.87362E-08	7.50936E-08	HSPB1/P4HB/HSP90AB1/HSPA1A/HSPD1/CLU/HSPA5/HSPA8/HSP90B1/HSPA6/PDIA3/HSPA9/CCT5/CCT8/PPIA/CCT2/PRDX4/PDIA6/FKBP10/DNAJB11	20
GO:0051702	biological process involved in interaction with symbiont	15/255	197/29151	2.49982E-10	8.87362E-08	7.50936E-08	APOL1/APOE/FN1/LTF/HRG/GAPDH/ARG1/HSPD1/HSPA8/CFL1/PTX3/PHB1/FASN/EEF1A1/DCD	15
GO:0010951	negative regulation of endopeptidase activity	21/255	429/29151	2.53605E-10	8.87362E-08	7.50936E-08	SERPINC1/SERPINA1/SERPINA3/AGT/A2M/C3/C5/CST4/CST1/CSTA/LTF/HRG/GAPDH/ANXA2/C4B/SERPINB3/PRDX5/LCN1/SFN/ITIH4/SERPINB12	21
GO:0072376	protein activation cascade	8/255	39/29151	1.49514E-09	4.75589E-07	4.0247E-07	SERPINC1/FGA/FGB/FGG/APOH/FN1/GP1BB/FLNA	8
GO:0050818	regulation of coagulation	12/255	141/29151	4.5526E-09	1.32746E-06	1.12337E-06	SERPINC1/APOE/FGA/FGB/FGG/APOH/F11/HRG/ANXA2/ANXA5/CD9/PRDX2	12
GO:0042744	hydrogen peroxide catabolic process	8/255	45/29151	5.00646E-09	1.34751E-06	1.14034E-06	HP/CAT/PRDX5/PRDX2/HBB/HBA1/PRDX1/PRDX4	8
GO:1903034	regulation of response to wounding	16/255	297/29151	9.59291E-09	2.39754E-06	2.02894E-06	SERPINC1/APOE/FGA/FGB/FGG/APOH/F11/ANXA1/HRG/ANXA2/FGF2/FLNA/CD9/PRDX2/ACTG1/TNR	16
GO:0050819	negative regulation of coagulation	10/255	95/29151	1.15784E-08	2.62602E-06	2.22229E-06	APOE/FGA/FGB/FGG/APOH/F11/HRG/ANXA2/ANXA5/CD9	10
GO:0031639	plasminogen activation	8/255	50/29151	1.20081E-08	2.62602E-06	2.22229E-06	PGK1/FGA/FGB/FGG/APOH/F11/ENO1/ANXA2	8
GO:0034109	homotypic cell-cell adhesion	13/255	190/29151	1.45621E-08	2.99723E-06	2.53642E-06	FGA/FGB/FGG/FN1/HSPB1/JUP/DSP/FLNA/CD9/MYH9/PPIA/ACTG1/HBB	13
GO:0034381	plasma lipoprotein particle clearance	9/255	75/29151	1.97804E-08	3.67964E-06	3.11392E-06	APOM/APOA1/APOE/APOA2/APOC3/APOB/ANXA2/HNRNPK/CNPY2	9
GO:0006956	complement activation	10/255	101/29151	2.10268E-08	3.67964E-06	3.11392E-06	IGLL5/C1R/CFB/A2M/C3/C5/C4BPA/C4B/CLU/PHB1	10
GO:0061041	regulation of wound healing	14/255	233/29151	2.10325E-08	3.67964E-06	3.11392E-06	SERPINC1/APOE/FGA/FGB/FGG/APOH/F11/ANXA1/HRG/ANXA2/FGF2/CD9/PRDX2/ACTG1	14
GO:1990748	cellular detoxification	13/255	200/29151	2.68276E-08	4.46999E-06	3.78276E-06	APOM/ALDH1A1/HP/APOE/CAT/S100A9/TXN/PRDX5/PRDX2/HBB/HBA1/PRDX1/PRDX4	13
GO:0030193	regulation of blood coagulation	11/255	134/29151	2.94793E-08	4.4847E-06	3.79521E-06	SERPINC1/APOE/FGA/FGB/FGG/APOH/F11/HRG/ANXA2/CD9/PRDX2	11
GO:0097006	regulation of plasma lipoprotein particle levels	11/255	134/29151	2.94793E-08	4.4847E-06	3.79521E-06	APOM/ACSL3/AGT/APOA1/APOE/APOA2/APOC3/APOB/ANXA2/HNRNPK/CNPY2	11
GO:0019730	antimicrobial humoral response	12/255	168/29151	3.27615E-08	4.77635E-06	4.04202E-06	JCHAIN/FGA/FGB/TF/LTF/HRG/GAPDH/S100A9/S100A7/LYZ/MUC7/DMBT1	12
GO:1900046	regulation of hemostasis	11/255	136/29151	3.43865E-08	4.81273E-06	4.07281E-06	SERPINC1/APOE/FGA/FGB/FGG/APOH/F11/HRG/ANXA2/CD9/PRDX2	11

GO:0030168	platelet activation	14/255	246/29151	4.15555E-08	5.59241E-06	4.73261E-06	APOE/FGA/FGB/FGG/FN1/HRG/HSPB1/GP1BB/FLNA/CD9/MYH9/PPIA/ACTG1/HBB	14
GO:0098869	cellular oxidant detoxification	12/255	173/29151	4.53565E-08	5.87786E-06	4.97418E-06	APOM/HP/APOE/CAT/S100A9/TXN/PRDX5/PRDX2/HBB/HBA1/PRDX1/PRDX4	12
GO:0097237	cellular response to toxic substance	13/255	215/29151	6.2935E-08	7.86462E-06	6.65549E-06	APOM/ALDH1A1/HP/APOE/CAT/S100A9/TXN/PRDX5/PRDX2/HBB/HBA1/PRDX1/PRDX4	13
GO:0070527	platelet aggregation	11/255	145/29151	6.66764E-08	8.04485E-06	6.80801E-06	FGA/FGB/FGG/FN1/HSPB1/FLNA/CD9/MYH9/PPIA/ACTG1/HBB	11
GO:0046034	ATP metabolic process	17/255	389/29151	7.05893E-08	8.23306E-06	6.96728E-06	LDHA/COX2/PGK1/ALDOA/GAPDH/ATP5F1B/ENO1/PARP1/HSPA1A/HSPA8/MYH6/PKM/PGAM1/ATP6V1B2/ATP5F1A/TPI1/TMSB4X	17
GO:0006953	acute-phase response	9/255	87/29151	7.37403E-08	8.23815E-06	6.97159E-06	HP/SERPINA1/SERPINA3/A2M/FN1/ORM1/ORM2/SAA4/ITIH4	9
GO:0009205	purine ribonucleoside triphosphate metabolic process	18/255	439/29151	7.53417E-08	8.23815E-06	6.97159E-06	LDHA/COX2/PGK1/ALDOA/GAPDH/ATP5F1B/ENO1/PARP1/HSPA1A/HSPA8/IMPDH2/MYH6/PKM/PGAM1/ATP6V1B2/ATP5F1A/TPI1/TMSB4X	18
GO:0071353	cellular response to interleukin-4	8/255	63/29151	7.85374E-08	8.32734E-06	7.04707E-06	ARG1/RPLP0/HSP90AB1/HSPA5/IMPDH2/RPL3/FASN/TUBA1B	8
GO:0034446	substrate adhesion-dependent cell spreading	13/255	224/29151	1.01582E-07	1.01941E-05	8.62685E-06	ACTN4/APOA1/FGA/FGB/FGG/FN1/ITGAV/P4HB/LAMB1/LAMC1/FLNA/LAMB2/RAB1A	13
GO:0009144	purine nucleoside triphosphate metabolic process	18/255	448/29151	1.0197E-07	1.01941E-05	8.62685E-06	LDHA/COX2/PGK1/ALDOA/GAPDH/ATP5F1B/ENO1/PARP1/HSPA1A/HSPA8/IMPDH2/MYH6/PKM/PGAM1/ATP6V1B2/ATP5F1A/TPI1/TMSB4X	18
GO:0030195	negative regulation of blood coagulation	9/255	91/29151	1.09308E-07	1.06241E-05	8.99072E-06	APOE/FGA/FGB/FGG/APOH/F11/HRG/ANXA2/CD9	9
GO:1900047	negative regulation of hemostasis	9/255	92/29151	1.20244E-07	1.10721E-05	9.36984E-06	APOE/FGA/FGB/FGG/APOH/F11/HRG/ANXA2/CD9	9
GO:0009199	ribonucleoside triphosphate metabolic process	18/255	453/29151	1.20246E-07	1.10721E-05	9.36984E-06	LDHA/COX2/PGK1/ALDOA/GAPDH/ATP5F1B/ENO1/PARP1/HSPA1A/HSPA8/IMPDH2/MYH6/PKM/PGAM1/ATP6V1B2/ATP5F1A/TPI1/TMSB4X	18
GO:0070670	response to interleukin-4	8/255	67/29151	1.28339E-07	1.15143E-05	9.74407E-06	ARG1/RPLP0/HSP90AB1/HSPA5/IMPDH2/RPL3/FASN/TUBA1B	8
GO:0031638	zymogen activation	10/255	123/29151	1.38772E-07	1.2139E-05	1.02728E-05	PGK1/C1R/HP/FGA/FGB/FGG/APOH/F11/ENO1/ANXA2	10
GO:1903035	negative regulation of response to wounding	11/255	160/29151	1.82152E-07	1.55451E-05	1.31552E-05	APOE/FGA/FGB/FGG/APOH/F11/HRG/ANXA2/FGF2/CD9/TNR	11
GO:0098754	detoxification	13/255	237/29151	1.95137E-07	1.60617E-05	1.35923E-05	APOM/ALDH1A1/HP/APOE/CAT/S100A9/TXN/PRDX5/PRDX2/HBB/HBA1/PRDX1/PRDX4	13
GO:0061045	negative regulation of wound healing	10/255	128/29151	2.01976E-07	1.60617E-05	1.35923E-05	APOE/FGA/FGB/FGG/APOH/F11/HRG/ANXA2/FGF2/CD9	10
GO:0061077	chaperone-mediated protein folding	10/255	128/29151	2.01976E-07	1.60617E-05	1.35923E-05	HSPB1/HSPA1A/CLU/HSPA5/HSPA8/HSPA6/HSPA9/CCT5/CCT8/CCT2	10
GO:0042730	fibrinolysis	7/255	49/29151	2.2666E-07	1.72811E-05	1.46243E-05	FGA/FGB/FGG/APOH/F11/HRG/ANXA2	7

GO:0042743	hydrogen peroxide metabolic process	9/255	99/29151	2.27189E-07	1.72811E-05	1.46243E-05	COX2/HP/CAT/PRDX5/PRDX2/HBB/HBA1/PRDX1/PRDX4	9
GO:0043691	reverse cholesterol transport	6/255	31/29151	2.58925E-07	1.90698E-05	1.6138E-05	APOM/APOA1/APOE/APOA2/APOC3/CLU	6
GO:0034377	plasma lipoprotein particle assembly	7/255	50/29151	2.61604E-07	1.90698E-05	1.6138E-05	APOM/ACSL3/APOA1/APOE/APOA2/APOC3/APOB	7
GO:0009141	nucleoside triphosphate metabolic process	18/255	480/29151	2.81918E-07	2.01313E-05	1.70362E-05	LDHA/COX2/PGK1/ALDOA/GAPDH/ATP5F1B/ENO1/PARP1/HSPA1A/HSPA8/IMPDH2/MYH6/PKM/PGAM1/ATP6V1B2/ATP5F1A/TPI1/TMSB4X	18
GO:0002526	acute inflammatory response	12/255	207/29151	3.20913E-07	2.24575E-05	1.90048E-05	HP/SERPINA1/SERPINA3/A2M/C3/APOA2/FN1/ORM1/S100A8/ORM2/SAA4/ITIH4	12
GO:0042026	protein refolding	7/255	53/29151	3.94796E-07	2.70861E-05	2.29218E-05	HSPB1/HSPA1A/HSPD1/HSPA5/HSPA8/HSPA6/HSPA9	7
GO:0044788	modulation by host of viral process	8/255	82/29151	6.26425E-07	4.21512E-05	3.56707E-05	APOE/LTF/HSPA8/CFL1/PTX3/PHB1/FASN/EEF1A1	8
GO:0034368	protein-lipid complex remodeling	7/255	58/29151	7.41974E-07	4.80772E-05	4.06856E-05	APOM/AGT/APOA1/APOE/APOA2/APOC3/APOB	7
GO:0034369	plasma lipoprotein particle remodeling	7/255	58/29151	7.41974E-07	4.80772E-05	4.06856E-05	APOM/AGT/APOA1/APOE/APOA2/APOC3/APOB	7
GO:0034384	high-density lipoprotein particle clearance	5/255	21/29151	8.93744E-07	5.68583E-05	4.81168E-05	APOM/APOA1/APOE/APOA2/APOC3	5
GO:0034367	protein-containing complex remodeling	7/255	60/29151	9.38971E-07	5.8669E-05	4.9649E-05	APOM/AGT/APOA1/APOE/APOA2/APOC3/APOB	7
GO:0071827	plasma lipoprotein particle organization	8/255	87/29151	9.89102E-07	6.0717E-05	5.13822E-05	APOM/ACSL3/AGT/APOA1/APOE/APOA2/APOC3/APOB	8
GO:0002455	humoral immune response mediated by circulating immunoglobulin	8/255	91/29151	1.39594E-06	8.42138E-05	7.12665E-05	IGLL5/C1R/C3/C5/HPX/C4BPA/C4B/CLU	8
GO:2001046	positive regulation of integrin-mediated signaling pathway	5/255	23/29151	1.4569E-06	8.5497E-05	7.23524E-05	LAMB1/LAMC1/NID1/FLNA/LAMB2	5
GO:0006958	complement activation, classical pathway	7/255	64/29151	1.46608E-06	8.5497E-05	7.23524E-05	IGLL5/C1R/C3/C5/C4BPA/C4B/CLU	7
GO:0065005	protein-lipid complex assembly	7/255	67/29151	2.00713E-06	0.00011513	9.74297E-05	APOM/ACSL3/APOA1/APOE/APOA2/APOC3/APOB	7
GO:0050821	protein stabilization	15/255	391/29151	2.13898E-06	0.000120714	0.000102155	APOA1/APOA2/GAPDH/NPM1/PFN1/HSP90AB1/HSPA1A/HSPD1/CLU/FLNA/PHB1/CCT5/CCT8/CCT2/DSG1	15
GO:0009620	response to fungus	8/255	98/29151	2.45231E-06	0.000136201	0.000115261	LTF/HRG/GAPDH/ARG1/S100A8/S100A9/PTX3/DCD	8
GO:0071825	protein-lipid complex subunit organization	8/255	104/29151	3.837E-06	0.000209776	0.000177524	APOM/ACSL3/AGT/APOA1/APOE/APOA2/APOC3/APOB	8
GO:0050832	defense response to fungus	7/255	74/29151	3.94291E-06	0.00021225	0.000179618	LTF/HRG/GAPDH/ARG1/S100A8/S100A9/DCD	7

GO:0034375	high-density lipoprotein particle remodeling	5/255	28/29151	4.10583E-06	0.000217671	0.000184206	APOM/APOA1/APOE/APOA2/APOC3	5
GO:0034113	heterotypic cell-cell adhesion	8/255	107/29151	4.74736E-06	0.000247926	0.000209809	APOA1/FGA/FGB/FGG/ITGAV/JUP/DSP/ITGA7	8
GO:0006458	'de novo' protein folding	7/255	77/29151	5.15291E-06	0.000265147	0.000224383	HSPA1A/HSPD1/HSPA5/HSPA8/HSPA6/HSPA9/CCT2	7
GO:0007597	blood coagulation, intrinsic pathway	4/255	14/29151	5.34339E-06	0.000270964	0.000229305	SERPINC1/APOH/GP1BB/FLNA	4
GO:0018149	peptide cross-linking	6/255	56/29151	9.5098E-06	0.000460758	0.000389919	CSTA/FN1/ANXA1/DSP/TGM1/TGM3	6
GO:0017014	protein nitrosylation	5/255	33/29151	9.56738E-06	0.000460758	0.000389919	GAPDH/S100A8/S100A9/TXN/DMD	5
GO:0018119	peptidyl-cysteine S-nitrosylation	5/255	33/29151	9.56738E-06	0.000460758	0.000389919	GAPDH/S100A8/S100A9/TXN/DMD	5
GO:0043534	blood vessel endothelial cell migration	11/255	240/29151	9.61284E-06	0.000460758	0.000389919	APOA1/APOE/ANXA1/HRG/HSPB1/ATP5F1B/FGF2/JUP/ATP5F1A/MYH9/TMSB4X	11
GO:0042742	defense response to bacterium	15/255	445/29151	1.01392E-05	0.000479419	0.000405712	IGLL5/HP/JCHAIN/FGA/FGB/TF/LTF/S100A8/S100A9/S100A7/LYZ/RAB1A/DCD/LACRT/DMBT1	15
GO:0034975	protein folding in endoplasmic reticulum	4/255	17/29151	1.24445E-05	0.00057939	0.000490312	P4HB/HSPA5/HSP90B1/PDIA3	4
GO:0030216	keratinocyte differentiation	11/255	247/29151	1.25846E-05	0.00057939	0.000490312	CSTA/ANXA1/DSP/TGM1/S100A7/CASP14/SFN/TGM3/NUMA1/PLEC/CDSN	11
GO:0070486	leukocyte aggregation	4/255	18/29151	1.58903E-05	0.000696505	0.000589422	S100A8/S100A9/MSN/JAM2	4
GO:1905918	regulation of CoA-transferase activity	4/255	18/29151	1.58903E-05	0.000696505	0.000589422	AGT/APOA1/APOE/APOA2	4
GO:1905920	positive regulation of CoA-transferase activity	4/255	18/29151	1.58903E-05	0.000696505	0.000589422	AGT/APOA1/APOE/APOA2	4
GO:1900182	positive regulation of protein localization to nucleus	9/255	165/29151	1.59247E-05	0.000696505	0.000589422	IPO5/NPM1/HSP90AB1/PARP1/JUP/FLNA/CCT5/CCT8/CCT2	9
GO:0060191	regulation of lipase activity	9/255	166/29151	1.67107E-05	0.000721861	0.00061088	AGT/APOA1/APOA2/APOC3/APOH/ANXA1/FGF2/PHB1/LRP1	9
GO:0051873	killing by host of symbiont cells	5/255	38/29151	1.9525E-05	0.000823108	0.00069656	APOL1/HRG/GAPDH/ARG1/DCD	5
GO:2001044	regulation of integrin-mediated signaling pathway	5/255	38/29151	1.9525E-05	0.000823108	0.00069656	LAMB1/LAMC1/NID1/FLNA/LAMB2	5
GO:0009636	response to toxic substance	14/255	424/29151	2.51629E-05	0.001032144	0.000873459	APOM/ALDH1A1/HP/APOE/CAT/ARG1/S100A9/TXN/PRDX5/PRDX2/HBB/HBA1/PRDX1/PRDX4	14
GO:0051651	maintenance of location in cell	14/255	424/29151	2.51629E-05	0.001032144	0.000873459	APOE/S100A8/S100A9/ANXA6/FGF2/CALM1/TXN/HSPA5/DMD/GP1BB/HSP90B1/FLNA/TMSB4X/LACRT	14
GO:0050909	sensory perception of taste	7/255	98/29151	2.53685E-05	0.001032144	0.000873459	CST4/CST1/PIGR/PIP/AZGP1/LCN1/GNB1	7
GO:0007229	integrin-mediated signaling pathway	10/255	221/29151	2.7299E-05	0.001097924	0.000929125	APOA1/FN1/ITGAV/LAMB1/LAMC1/NID1/FLNA/MYH9/LAMB2/ITGA7	10

GO:0110011	regulation of basement membrane organization	4/255	21/29151	3.04441E-05	0.001210499	0.001024393	LAMB1/LAMC1/NID1/LAMB2	4
GO:0033344	cholesterol efflux	7/255	101/29151	3.08463E-05	0.001212709	0.001026263	APOM/APOA1/APOE/APOA2/APOC3/APOB/LRP1	7
GO:0051084	'de novo' post-translational protein folding	6/255	69/29151	3.19341E-05	0.001241528	0.001050651	HSPA1A/HSPA5/HSPA8/HSPA6/HSPA9/CCT2	6
GO:0002181	cytoplasmic translation	11/255	275/29151	3.38833E-05	0.001302832	0.00110253	SYNCRIP/RPLP0/PKM/RPL4/RPL3/EIF4A1/RPS3A/RPL15/RPL37A/RPL8/RPS27A	11
GO:0045454	cell redox homeostasis	6/255	70/29151	3.46743E-05	0.001318753	0.001116004	DLD/TXN/PRDX5/PRDX2/PRDX1/PRDX4	6
GO:0051131	chaperone-mediated protein complex assembly	5/255	43/29151	3.6133E-05	0.001359457	0.00115045	HSP90AB1/HSPA1A/HSPD1/CLU/CCT2	5
GO:0008037	cell recognition	12/255	329/29151	3.68876E-05	0.001373083	0.00116198	IGLL5/C4BPA/ALDOA/C4B/VCAN/CD9/PTX3/MSN/CCT5/CCT8/YWHAZ/CCT2	12
GO:0030301	cholesterol transport	9/255	186/29151	4.09707E-05	0.001493297	0.001263713	APOM/APOA1/APOE/APOA2/APOC3/APOB/ANXA2/CLU/LRP1	9
GO:0043535	regulation of blood vessel endothelial cell migration	9/255	186/29151	4.09707E-05	0.001493297	0.001263713	APOE/ANXA1/HRG/HSPB1/ATP5F1B/FGF2/JUP/ATP5F1A/TMSB4X	9
GO:0051851	modulation by host of symbiont process	8/255	145/29151	4.36151E-05	0.00157329	0.001331407	APOE/LTF/HSPA8/CFL1/PTX3/PHB1/FASN/EEF1A1	8
GO:0033700	phospholipid efflux	4/255	23/29151	4.44274E-05	0.00158624	0.001342366	APOA1/APOE/APOA2/APOC3	4
GO:0001580	detection of chemical stimulus involved in sensory perception of bitter taste	5/255	46/29151	5.03662E-05	0.001780116	0.001506435	CST4/CST1/PIGR/PIP/AZGP1	5
GO:0034370	triglyceride-rich lipoprotein particle remodeling	4/255	24/29151	5.29474E-05	0.001834288	0.001552279	APOA1/APOE/APOA2/APOC3	4
GO:0034372	very-low-density lipoprotein particle remodeling	4/255	24/29151	5.29474E-05	0.001834288	0.001552279	APOA1/APOE/APOA2/APOC3	4
GO:0009913	epidermal cell differentiation	12/255	348/29151	6.32527E-05	0.002169817	0.001836222	CSTA/ANXA1/FGF2/DSP/TGM1/S100A7/CASP14/SFN/TGM3/NUMA1/PLEC/CDSN	12
GO:0000302	response to reactive oxygen species	13/255	406/29151	6.68524E-05	0.002265754	0.00191741	HP/APOE/CAT/ANXA1/ARG1/APOD/TXN/PRDX5/S100A7/PRDX2/HBB/HBA1/PRDX1	13
GO:0042157	lipoprotein metabolic process	10/255	246/29151	6.73445E-05	0.002265754	0.00191741	APOL1/APOM/APOA1/APOE/APOA2/APOC3/APOB/APOD/ITGAV/CTSD	10
GO:0019731	antibacterial humoral response	6/255	79/29151	6.88443E-05	0.002294156	0.001941445	JCHAIN/FGA/FGB/TF/LTF/DMBT1	6
GO:0010988	regulation of low-density lipoprotein particle clearance	4/255	26/29151	7.34758E-05	0.002425396	0.002052508	APOC3/ANXA2/HNRNPK/CNPY2	4
GO:1905599	positive regulation of low-density	3/255	10/29151	7.58591E-05	0.002480665	0.00209928	ANXA2/HNRNPK/CNPY2	3

	lipoprotein receptor activity							
GO:0010811	positive regulation of cell-substrate adhesion	10/255	252/29151	8.22453E-05	0.002664597	0.002254933	APOA1/FGA/FGB/FGG/FN1/HRG/P4HB/NID1/JUP/FLNA	10
GO:0006096	glycolytic process	8/255	160/29151	8.73063E-05	0.002802612	0.00237173	LDHA/PGK1/ALDOA/GAPDH/ENO1/PKM/PGAM1/TPI1	8
GO:0006090	pyruvate metabolic process	9/255	206/29151	9.00583E-05	0.002864672	0.002424249	LDHA/PGK1/ALDOA/GAPDH/ENO1/DLD/PKM/PGAM1/TPI1	9
GO:0006757	ATP generation from ADP	8/255	161/29151	9.1187E-05	0.002874445	0.002432519	LDHA/PGK1/ALDOA/GAPDH/ENO1/PKM/PGAM1/TPI1	8
GO:0034383	low-density lipoprotein particle clearance	5/255	53/29151	0.000100319	0.00313407	0.002652228	APOC3/APOB/ANXA2/HNRNPK/CNPY2	5
GO:0017038	protein import	3/255	11/29151	0.000103632	0.003180773	0.002691751	APOE/CLU/HSPA8	3
GO:0060699	regulation of endoribonuclease activity	3/255	11/29151	0.000103632	0.003180773	0.002691751	NPM1/HSPA1A/ABCE1	3
GO:0050913	sensory perception of bitter taste	5/255	54/29151	0.000109772	0.00330891	0.002800188	CST4/CST1/PIGR/PIP/AZGP1	5
GO:1900180	regulation of protein localization to nucleus	10/255	261/29151	0.000109796	0.00330891	0.002800188	IPO5/APOD/NPM1/HSP90AB1/PARP1/JUP/FLNA/CCT5/CCT8/CCT2	10
GO:0043542	endothelial cell migration	13/255	427/29151	0.000110644	0.00330891	0.002800188	AGT/APOA1/APOE/APOH/ANXA1/HRG/HSPB1/ATP5F1B/FGF2/JUP/ATP5F1A/MYH9/TMSB4X	13
GO:0010810	regulation of cell-substrate adhesion	13/255	428/29151	0.000113236	0.003357743	0.002841513	ACTN4/APOA1/FGA/FGB/FGG/FN1/HRG/APOD/P4HB/NID1/JUP/FLNA/ACTG1	13
GO:0034380	high-density lipoprotein particle assembly	4/255	29/29151	0.000114349	0.003362254	0.002845331	APOM/APOA1/APOE/APOA2	4
GO:1900024	regulation of substrate adhesion-dependent cell spreading	7/255	125/29151	0.00011975	0.003467022	0.002933991	ACTN4/APOA1/FGA/FGB/FGG/P4HB/FLNA	7
GO:0050912	detection of chemical stimulus involved in sensory perception of taste	5/255	55/29151	0.000119894	0.003467022	0.002933991	CST4/CST1/PIGR/PIP/AZGP1	5
GO:0015918	sterol transport	9/255	217/29151	0.000133598	0.003831631	0.003242544	APOM/APOA1/APOE/APOA2/APOC3/APOB/ANXA2/CLU/LRP1	9
GO:0010901	regulation of very-low-density lipoprotein particle remodeling	3/255	12/29151	0.000137283	0.003905307	0.003304893	APOA1/APOA2/APOC3	3
GO:0051085	chaperone cofactor-dependent protein refolding	5/255	58/29151	0.00015459	0.004362182	0.003691526	HSPA1A/HSPA5/HSPA8/HSPA6/HSPA9	5
GO:1900026	positive regulation of substrate adhesion-dependent cell spreading	6/255	92/29151	0.000160677	0.004497679	0.003806192	APOA1/FGA/FGB/FGG/P4HB/FLNA	6

GO:0034374	low-density lipoprotein particle remodeling	4/255	32/29151	0.0001696	0.004709778	0.003985682	AGT/APOE/APOA2/APOB	4
GO:0010594	regulation of endothelial cell migration	11/255	332/29151	0.000180388	0.004969916	0.004205825	AGT/APOE/APOH/ANXA1/HRG/HSPB1/ATP5F1B/FGF2/JUP/ATP5F1A/TMSB4X	11
GO:0001906	cell killing	11/255	334/29151	0.000189986	0.005080813	0.004299673	C3/LTF/GAPDH/ARG1/TUBB/HSP90AB1/AZGP1/LYZ/DCD/PRDX1/MUC7	11
GO:0006735	NADH regeneration	4/255	33/29151	0.000191674	0.005080813	0.004299673	PGK1/ENO1/PKM/TPI1	4
GO:0030449	regulation of complement activation	4/255	33/29151	0.000191674	0.005080813	0.004299673	A2M/C3/C4BPA/PHB1	4
GO:0061621	canonical glycolysis	4/255	33/29151	0.000191674	0.005080813	0.004299673	PGK1/ENO1/PKM/TPI1	4
GO:0061718	glucose catabolic process to pyruvate	4/255	33/29151	0.000191674	0.005080813	0.004299673	PGK1/ENO1/PKM/TPI1	4
GO:0046031	ADP metabolic process	8/255	182/29151	0.000212206	0.005582763	0.004724451	LDHA/PGK1/ALDOA/GAPDH/ENO1/PKM/PGAM1/TPI1	8
GO:0010543	regulation of platelet activation	6/255	98/29151	0.000227076	0.005929386	0.005017783	APOE/FGG/HRG/GP1BB/FLNA/CD9	6
GO:0044794	positive regulation by host of viral process	4/255	35/29151	0.000241919	0.006270184	0.005306186	APOE/HSPA8/CFL1/EEF1A1	4
GO:0043588	skin development	13/255	463/29151	0.000243858	0.006273954	0.005309376	CSTA/ANXA1/COL1A2/JUP/DSP/TGM1/S100A7/CASP14/SFN/TGM3/NUMA1/PLEC/CDSN	13
GO:0006165	nucleoside diphosphate phosphorylation	8/255	187/29151	0.000255009	0.006512963	0.005511639	LDHA/PGK1/ALDOA/GAPDH/ENO1/PKM/PGAM1/TPI1	8
GO:0010984	regulation of lipoprotein particle clearance	4/255	36/29151	0.0002703	0.006755558	0.005716937	APOC3/ANXA2/HNRNPK/CNPY2	4
GO:0051238	sequestering of metal ion	4/255	36/29151	0.0002703	0.006755558	0.005716937	ANXA1/S100A8/S100A9/S100A7	4
GO:0061620	glycolytic process through glucose-6-phosphate	4/255	36/29151	0.0002703	0.006755558	0.005716937	PGK1/ENO1/PKM/TPI1	4
GO:0046939	nucleotide phosphorylation	8/255	190/29151	0.000283916	0.007018068	0.005939088	LDHA/PGK1/ALDOA/GAPDH/ENO1/PKM/PGAM1/TPI1	8
GO:0031640	killing of cells of another organism	5/255	66/29151	0.000284867	0.007018068	0.005939088	LTF/GAPDH/LYZ/DCD/MUC7	5
GO:0051092	positive regulation of NF-kappaB transcription factor activity	10/255	294/29151	0.00028682	0.007018068	0.005939088	AGT/LTF/CAT/S100A8/S100A9/NPM1/HSPA1A/CLU/PSMA6/PPIA	10
GO:0061615	glycolytic process through fructose-6-phosphate	4/255	37/29151	0.000300993	0.007303157	0.006180347	PGK1/ENO1/PKM/TPI1	4
GO:0035966	response to topologically incorrect protein	10/255	296/29151	0.000302646	0.007303157	0.006180347	HSPB1/HSP90AB1/HSPA1A/HSPD1/CLU/HSPA5/HSPA8/HSPA6/HSPA9/CREBRF	10
GO:0006641	triglyceride metabolic process	8/255	194/29151	0.000326572	0.007826541	0.006623264	C3/APOA1/APOE/APOA2/APOC3/APOH/CAT/APOB	8

GO:0009206	purine ribonucleoside triphosphate biosynthetic process	8/255	198/29151	0.000374317	0.00890977	0.007539954	COX2/ALDOA/ATP5F1B/ENO1/PARP1/IMPDH2/ATP5F1A/TMSB4X	8
GO:0006986	response to unfolded protein	9/255	250/29151	0.000381076	0.008955526	0.007578675	HSPB1/HSP90AB1/HSPA1A/HSPD1/HSPA5/HSPA8/HSPA6/HSPA9/CREBRF	9
GO:0001649	osteoblast differentiation	12/255	423/29151	0.000383541	0.008955526	0.007578675	SYNCRIP/LTF/CAT/ATP5F1B/FGF2/VCAN/TNC/PHB1/FASN/TPM4/SMOC1/RRBP1	12
GO:1903053	regulation of extracellular matrix organization	6/255	108/29151	0.000383918	0.008955526	0.007578675	AGT/LAMB1/LAMC1/NID1/LAMB2/LRP1	6
GO:0009145	purine nucleoside triphosphate biosynthetic process	8/255	200/29151	0.000400237	0.009213349	0.007796859	COX2/ALDOA/ATP5F1B/ENO1/PARP1/IMPDH2/ATP5F1A/TMSB4X	8
GO:0046822	regulation of nucleocytoplasmic transport	8/255	200/29151	0.000400237	0.009213349	0.007796859	IPO5/APOD/HSP90AB1/TXN/JUP/FLNA/SFN/YWHAE	8
GO:0061844	antimicrobial humoral immune response mediated by antimicrobial peptide	6/255	109/29151	0.000403348	0.009224283	0.007806113	LTF/HRG/GAPDH/S100A9/S100A7/MUC7	6
GO:0070508	cholesterol import	3/255	17/29151	0.00041081	0.009333917	0.007898891	APOA1/APOA2/APOC3	3
GO:0043903	regulation of biological process involved in symbiotic interaction	6/255	111/29151	0.000444508	0.010034406	0.008491684	LTF/ARG1/ITGAV/P4HB/LGALS1/PTX3	6
GO:0002920	regulation of humoral immune response	5/255	73/29151	0.000455604	0.01021896	0.008647864	A2M/C3/HPX/C4BPA/PHB1	5
GO:0043536	positive regulation of blood vessel endothelial cell migration	6/255	112/29151	0.000466279	0.010310504	0.008725334	ANXA1/HSPB1/ATP5F1B/FGF2/ATP5F1A/TMSB4X	6
GO:1905952	regulation of lipid localization	10/255	313/29151	0.000469078	0.010310504	0.008725334	AGT/C3/APOA1/APOE/APOA2/APOC3/APOB/ITGAV/ANXA2/LRP1	10
GO:0009135	purine nucleoside diphosphate metabolic process	8/255	205/29151	0.000471472	0.010310504	0.008725334	LDHA/PGK1/ALDOA/GAPDH/ENO1/PKM/PGAM1/TPI1	8
GO:0009179	purine ribonucleoside diphosphate metabolic process	8/255	205/29151	0.000471472	0.010310504	0.008725334	LDHA/PGK1/ALDOA/GAPDH/ENO1/PKM/PGAM1/TPI1	8
GO:0016485	protein processing	12/255	434/29151	0.000481783	0.01047055	0.008860775	PGK1/C1R/HP/FGA/FGB/FGG/APOH/F11/ENO1/ANXA2/PARP1/MYH9	12
GO:0060700	regulation of ribonuclease activity	3/255	18/29151	0.000489794	0.010578938	0.008952498	NPM1/HSPA1A/ABCE1	3
GO:0046824	positive regulation of	6/255	115/29151	0.000536608	0.011518961	0.009747999	IPO5/HSP90AB1/JUP/FLNA/SFN/YWHAE	6

	nucleocytoplasmic transport							
GO:0010632	regulation of epithelial cell migration	12/255	441/29151	0.000554735	0.011835482	0.010015858	AGT/APOE/APOH/ANXA1/HRG/HSPB1/ATP5F1B/PFN1/FGF2/JUP/ATP5F1A/TMSB4X	12
GO:0009201	ribonucleoside triphosphate biosynthetic process	8/255	211/29151	0.000570185	0.012091383	0.010232415	COX2/ALDOA/ATP5F1B/ENO1/PARP1/IMPDH2/ATP5F1A/TMSB4X	8
GO:0032371	regulation of sterol transport	6/255	117/29151	0.000587892	0.012282375	0.010394044	APOA1/APOE/APOA2/APOC3/ANXA2/LRP1	6
GO:0032374	regulation of cholesterol transport	6/255	117/29151	0.000587892	0.012282375	0.010394044	APOA1/APOE/APOA2/APOC3/ANXA2/LRP1	6
GO:2001169	regulation of ATP biosynthetic process	4/255	44/29151	0.000589723	0.012282375	0.010394044	COX2/ENO1/PARP1/TMSB4X	4
GO:0045429	positive regulation of nitric oxide biosynthetic process	5/255	78/29151	0.000618083	0.012796878	0.010829445	AGT/HSP90AB1/CLU/PTX3/HBB	5
GO:0032489	regulation of Cdc42 protein signal transduction	3/255	20/29151	0.000675482	0.013821702	0.011696709	APOA1/APOE/APOC3	3
GO:0034379	very-low-density lipoprotein particle assembly	3/255	20/29151	0.000675482	0.013821702	0.011696709	ACSL3/APOC3/APOB	3
GO:2001026	regulation of endothelial cell chemotaxis	4/255	46/29151	0.000699272	0.014225307	0.012038263	HRG/HSPB1/FGF2/TMSB4X	4
GO:0006734	NADH metabolic process	5/255	81/29151	0.00073439	0.014767996	0.012497517	PGK1/ENO1/PKM/MDH2/TPI1	5
GO:1904407	positive regulation of nitric oxide metabolic process	5/255	82/29151	0.000776568	0.0154387	0.013065105	AGT/HSP90AB1/CLU/PTX3/HBB	5
GO:0034114	regulation of heterotypic cell-cell adhesion	4/255	48/29151	0.000822486	0.01625921	0.013759467	APOA1/FGA/FGB/FGG	4
GO:0009185	ribonucleoside diphosphate metabolic process	8/255	224/29151	0.000841735	0.016546244	0.014002372	LDHA/PGK1/ALDOA/GAPDH/ENO1/PKM/PGAM1/TPI1	8
GO:0018198	peptidyl-cysteine modification	5/255	84/29151	0.000866315	0.016934284	0.014330753	GAPDH/S100A8/S100A9/TXN/DMD	5
GO:1903036	positive regulation of response to wounding	6/255	129/29151	0.000980332	0.019056558	0.016126743	APOH/ANXA1/HRG/FLNA/PRDX2/ACTG1	6
GO:0009142	nucleoside triphosphate biosynthetic process	8/255	230/29151	0.000997932	0.019291511	0.016325573	COX2/ALDOA/ATP5F1B/ENO1/PARP1/IMPDH2/ATP5F1A/TMSB4X	8
GO:1904851	positive regulation of establishment of protein localization to telomere	3/255	23/29151	0.001029236	0.01978735	0.01674518	CCT5/CCT8/CCT2	3
GO:0006007	glucose catabolic process	4/255	51/29151	0.001034904	0.019787593	0.016745386	PGK1/ENO1/PKM/TPI1	4
GO:0006754	ATP biosynthetic process	7/255	179/29151	0.001046831	0.019906849	0.016846307	COX2/ALDOA/ATP5F1B/ENO1/PARP1/ATP5F1A/TMSB4X	7

GO:0008360	regulation of cell shape	9/255	289/29151	0.001069381	0.020225758	0.017116185	PLXNB2/FN1/ALDOA/ANXA1/MSN/MYH9/MYH10/ITGA7/SHROOM3	9
GO:2001242	regulation of intrinsic apoptotic signaling pathway	10/255	350/29151	0.001101041	0.020712592	0.017528173	HSPB1/S100A8/S100A9/ENO1/P4HB/PARP1/HSPA1A/CLU/HNRNPK/PPIA	10
GO:0048678	response to axon injury	7/255	182/29151	0.00115267	0.021409962	0.018118326	ARG1/APOD/FGF2/FLNA/TNC/LAMB2/TNR	7
GO:0003334	keratinocyte development	3/255	24/29151	0.001168706	0.021409962	0.018118326	SFN/PLEC/CDSN	3
GO:0070203	regulation of establishment of protein localization to telomere	3/255	24/29151	0.001168706	0.021409962	0.018118326	CCT5/CCT8/CCT2	3
GO:1904869	regulation of protein localization to Cajal body	3/255	24/29151	0.001168706	0.021409962	0.018118326	CCT5/CCT8/CCT2	3
GO:1904871	positive regulation of protein localization to Cajal body	3/255	24/29151	0.001168706	0.021409962	0.018118326	CCT5/CCT8/CCT2	3
GO:1901654	response to ketone	11/255	416/29151	0.001177876	0.021465563	0.01816538	A2M/ARG1/RPLP0/PARP1/CALM1/F5/ATP5F1A/MSN/GNB1/DSG1/NCOA4	11
GO:0031665	negative regulation of lipopolysaccharide-mediated signaling pathway	3/255	25/29151	0.001319539	0.023556458	0.019934814	LTF/PRDX2/LACRT	3
GO:0070202	regulation of establishment of protein localization to chromosome	3/255	25/29151	0.001319539	0.023556458	0.019934814	CCT5/CCT8/CCT2	3
GO:1903405	protein localization to nuclear body	3/255	25/29151	0.001319539	0.023556458	0.019934814	CCT5/CCT8/CCT2	3
GO:1904867	protein localization to Cajal body	3/255	25/29151	0.001319539	0.023556458	0.019934814	CCT5/CCT8/CCT2	3
GO:0016064	immunoglobulin mediated immune response	9/255	299/29151	0.001353339	0.024037225	0.020341666	IGLL5/C1R/C3/C5/HPX/C4BPA/C4B/HSPD1/CLU	9
GO:0006639	acylglycerol metabolic process	8/255	243/29151	0.001416147	0.025025757	0.021178218	C3/APOA1/APOE/APOA2/APOC3/APOH/CAT/APOB	8
GO:0006638	neutral lipid metabolic process	8/255	245/29151	0.001491287	0.026221176	0.022189849	C3/APOA1/APOE/APOA2/APOC3/APOH/CAT/APOB	8
GO:0019724	B cell mediated immunity	9/255	305/29151	0.001550984	0.027134457	0.022962719	IGLL5/C1R/C3/C5/HPX/C4BPA/C4B/HSPD1/CLU	9
GO:0006910	phagocytosis, recognition	4/255	57/29151	0.00157026	0.027298206	0.023101294	IGLL5/C4BPA/C4B/PTX3	4
GO:0031424	keratinization	5/255	96/29151	0.001575947	0.027298206	0.023101294	TGM1/CASP14/SFN/TGM3/CDSN	5
GO:0051208	sequestering of calcium ion	8/255	249/29151	0.001651048	0.02786784	0.02358335	ANXA6/FGF2/CALM1/DMD/GP1BB/HSP90B1/FLNA/LACRT	8
GO:0042159	lipoprotein catabolic process	3/255	27/29151	0.001656619	0.02786784	0.02358335	APOE/APOB/CTSD	3
GO:0060192	negative regulation of lipase activity	3/255	27/29151	0.001656619	0.02786784	0.02358335	APOA2/APOC3/ANXA1	3

GO:0098760	response to interleukin-7	3/255	27/29151	0.001656619	0.02786784	0.02358335	ATP5F1B/P4HB/PDIA3	3
GO:0098761	cellular response to interleukin-7	3/255	27/29151	0.001656619	0.02786784	0.02358335	ATP5F1B/P4HB/PDIA3	3
GO:1904816	positive regulation of protein localization to chromosome, telomeric region	3/255	27/29151	0.001656619	0.02786784	0.02358335	CCT5/CCT8/CCT2	3
GO:0010596	negative regulation of endothelial cell migration	5/255	98/29151	0.001726423	0.028903137	0.024459477	APOE/APOH/HRG/FGF2/JUP	5
GO:0010769	regulation of cell morphogenesis involved in differentiation	7/255	196/29151	0.001762357	0.029364228	0.024849679	ACTN4/APOA1/FGA/FGB/FGG/P4HB/FLNA	7
GO:0046890	regulation of lipid biosynthetic process	9/255	311/29151	0.001771185	0.02937145	0.02485579	IDH1/ACSL3/C3/APOA1/APOE/APOC3/ANXA1/APOB/FABP5	9
GO:0006656	phosphatidylcholine biosynthetic process	4/255	59/29151	0.001784755	0.029456872	0.024928079	ACSL3/APOA1/APOA2/FABP5	4
GO:0006826	iron ion transport	5/255	99/29151	0.00180549	0.029659195	0.025099297	CP/TF/LTF/HPX/HRG	5
GO:0008228	opsonization	3/255	28/29151	0.001843503	0.029725421	0.02515534	C4BPA/C4B/PTX3	3
GO:0010269	response to selenium ion	3/255	28/29151	0.001843503	0.029725421	0.02515534	APOB/ARG1/RPLP0	3
GO:0034116	positive regulation of heterotypic cell-cell adhesion	3/255	28/29151	0.001843503	0.029725421	0.02515534	FGA/FGB/FGG	3
GO:1990173	protein localization to nucleoplasm	3/255	28/29151	0.001843503	0.029725421	0.02515534	CCT5/CCT8/CCT2	3
GO:0032368	regulation of lipid transport	8/255	254/29151	0.001869482	0.030006038	0.025392815	AGT/APOA1/APOE/APOA2/APOC3/ITGAV/ANXA2/LRP1	8
GO:0035036	sperm-egg recognition	5/255	100/29151	0.001887176	0.030151731	0.025516108	ALDOA/CD9/CCT5/CCT8/CCT2	5
GO:0009132	nucleoside diphosphate metabolic process	8/255	255/29151	0.001915784	0.030469675	0.025785171	LDHA/PGK1/ALDOA/GAPDH/ENO1/PKM/PGAM1/TPI1	8
GO:0046390	ribose phosphate biosynthetic process	11/255	444/29151	0.00196687	0.031140628	0.026352969	ACSL3/COX2/ALDOA/ATP5F1B/ENO1/DLD/PARP1/IMPDH2/ATP5F1A/TKT/TMSB4X	11
GO:0045216	cell-cell junction organization	10/255	380/29151	0.00201943	0.031828768	0.026935312	AGT/JUP/DSP/FLNA/CD9/ACTG1/DSG1/PKP1/PLEC/POF1B	10
GO:1904814	regulation of protein localization to chromosome, telomeric region	3/255	29/29151	0.002043021	0.031913075	0.027006657	CCT5/CCT8/CCT2	3
GO:2001171	positive regulation of ATP biosynthetic process	3/255	29/29151	0.002043021	0.031913075	0.027006657	COX2/ENO1/TMSB4X	3
GO:0030100	regulation of endocytosis	11/255	447/29151	0.002072259	0.032225933	0.027271416	C3/APOE/APOC3/TF/ITGAV/ANXA2/CALM1/CLU/HNRNPK/LRP1/ARFGAP1	11
GO:0085029	extracellular matrix assembly	5/255	104/29151	0.00224115	0.034545298	0.029234194	AGT/LAMB1/COL1A2/LAMB2/FKBP10	5

GO:0006909	phagocytosis	11/255	452/29151	0.002258089	0.03465374	0.029325964	IGLL5/C3/APOA1/APOA2/C4BPA/ANXA1/ITGAV/C4B/PTX3/MYH9/LRP1	11
GO:0043277	apoptotic cell clearance	5/255	105/29151	0.002336708	0.035423587	0.029977453	C3/ANXA1/ITGAV/C4B/LRP1	5
GO:0090303	positive regulation of wound healing	5/255	105/29151	0.002336708	0.035423587	0.029977453	APOH/ANXA1/HRG/PRDX2/ACTG1	5
GO:0009988	cell-cell recognition	6/255	153/29151	0.002338625	0.035423587	0.029977453	ALDOA/CD9/MSN/CCT5/CCT8/CCT2	6
GO:0051701	biological process involved in interaction with host	10/255	389/29151	0.002391523	0.036068703	0.030523386	LTF/ITGAV/P4HB/HSP90AB1/LGALS1/HSPA1A/PTX3/SERPINB3/PHB1/PPIA	10
GO:1902175	regulation of oxidative stress-induced intrinsic apoptotic signaling pathway	4/255	64/29151	0.002408249	0.036165076	0.030604942	HSPB1/P4HB/PARP1/PPIA	4
GO:0006957	complement activation, alternative pathway	3/255	31/29151	0.00248111	0.036942146	0.031262543	CFB/C3/C5	3
GO:0035767	endothelial cell chemotaxis	4/255	66/29151	0.002694838	0.039824649	0.033701881	HRG/HSPB1/FGF2/TMSB4X	4
GO:0002544	chronic inflammatory response	3/255	32/29151	0.002720232	0.039824649	0.033701881	S100A8/S100A9/AHCY	3
GO:0051004	regulation of lipoprotein lipase activity	3/255	32/29151	0.002720232	0.039824649	0.033701881	APOA1/APOC3/APOH	3
GO:1904874	positive regulation of telomerase RNA localization to Cajal body	3/255	32/29151	0.002720232	0.039824649	0.033701881	CCT5/CCT8/CCT2	3
GO:0045807	positive regulation of endocytosis	7/255	212/29151	0.002737819	0.039908266	0.033772642	C3/APOE/TF/ANXA2/CLU/HNRNPK/LRP1	7
GO:0045428	regulation of nitric oxide biosynthetic process	5/255	109/29151	0.002748755	0.039908266	0.033772642	AGT/HSP90AB1/CLU/PTX3/HBB	5
GO:0010595	positive regulation of endothelial cell migration	7/255	213/29151	0.00281024	0.040632347	0.034385401	AGT/ANXA1/HSPB1/ATP5F1B/FGF2/ATP5F1A/TMSB4X	7
GO:0010770	positive regulation of cell morphogenesis involved in differentiation	6/255	160/29151	0.002921285	0.041891708	0.035451144	APOA1/FGA/FGB/FGG/P4HB/FLNA	6
GO:0051917	regulation of fibrinolysis	3/255	33/29151	0.002973088	0.042292283	0.035790133	APOH/F11/HRG	3
GO:0042306	regulation of protein import into nucleus	5/255	111/29151	0.002973393	0.042292283	0.035790133	IPO5/APOD/HSP90AB1/JUP/FLNA	5
GO:0043537	negative regulation of blood vessel endothelial cell migration	4/255	68/29151	0.003003965	0.042554147	0.036011738	APOE/HRG/FGF2/JUP	4
GO:0006006	glucose metabolic process	9/255	337/29151	0.003031604	0.042772508	0.036196527	PGK1/GAPDH/APOD/ENO1/PKM/MDH2/TPI1/FABP5/FAM3C	9

GO:0048260	positive regulation of receptor-mediated endocytosis	5/255	112/29151	0.003090548	0.043429022	0.036752106	C3/TF/ANXA2/CLU/HNRNPK	5
GO:0007160	cell-matrix adhesion	11/255	472/29151	0.003141452	0.043967766	0.037208022	FGA/FGB/FGG/FN1/HRG/APOD/ITGAV/NID1/JUP/ACTG1/ITGA7	11
GO:0009152	purine ribonucleotide biosynthetic process	10/255	405/29151	0.003187749	0.044241119	0.037439349	ACSL3/COX2/ALDOA/ATP5F1B/ENO1/DLD/PARP1/IMPDH2/ATP5F1A/TMSB4X	10
GO:0032488	Cdc42 protein signal transduction	3/255	34/29151	0.003239927	0.044241119	0.037439349	APOA1/APOE/APOC3	3
GO:0061684	chaperone-mediated autophagy	3/255	34/29151	0.003239927	0.044241119	0.037439349	CLU/HSPA8/EEF1A1	3
GO:0032799	low-density lipoprotein receptor particle metabolic process	2/255	10/29151	0.003274778	0.044241119	0.037439349	APOE/ANXA2	2
GO:0032802	low-density lipoprotein particle receptor catabolic process	2/255	10/29151	0.003274778	0.044241119	0.037439349	APOE/ANXA2	2
GO:0032803	regulation of low-density lipoprotein particle receptor catabolic process	2/255	10/29151	0.003274778	0.044241119	0.037439349	APOE/ANXA2	2
GO:0038001	paracrine signaling	2/255	10/29151	0.003274778	0.044241119	0.037439349	FGF2/SERPINB3	2
GO:0050748	negative regulation of lipoprotein metabolic process	2/255	10/29151	0.003274778	0.044241119	0.037439349	APOD/ITGAV	2
GO:0097638	L-arginine import across plasma membrane	2/255	10/29151	0.003274778	0.044241119	0.037439349	AGT/ARG1	2
GO:0016052	carbohydrate catabolic process	8/255	279/29151	0.003325928	0.044558436	0.037707881	LDHA/PGK1/ALDOA/GAPDH/ENO1/PKM/PGAM1/TPI1	8
GO:0090207	regulation of triglyceride metabolic process	4/255	70/29151	0.003336471	0.044558436	0.037707881	C3/APOA1/APOE/APOC3	4
GO:0042632	cholesterol homeostasis	6/255	165/29151	0.003399519	0.045227824	0.038274355	APOM/APOA1/APOE/APOA2/APOC3/APOB	6
GO:0080164	regulation of nitric oxide metabolic process	5/255	115/29151	0.003461979	0.045884333	0.03882993	AGT/HSP90AB1/CLU/PTX3/HBB	5
GO:0055092	sterol homeostasis	6/255	166/29151	0.003501762	0.046236477	0.039127934	APOM/APOA1/APOE/APOA2/APOC3/APOB	6
GO:0070200	establishment of protein localization to telomere	3/255	35/29151	0.00352099	0.046315584	0.039194879	CCT5/CCT8/CCT2	3
GO:0042307	positive regulation of protein import into nucleus	4/255	72/29151	0.00369318	0.048398641	0.04095768	IPO5/HSP90AB1/JUP/FLNA	4
GO:0048259	regulation of receptor-mediated endocytosis	7/255	224/29151	0.00370753	0.048405399	0.040963399	C3/APOC3/TF/ITGAV/ANXA2/CLU/HNRNPK	7

GO:0044827	modulation by host of viral genome replication	3/255	36/29151	0.003816508	0.04944974	0.04184718	HSPA8/PHB1/EEF1A1	3
GO:1904872	regulation of telomerase RNA localization to Cajal body	3/255	36/29151	0.003816508	0.04944974	0.04184718	CCT5/CCT8/CCT2	3
GO:0046461	neutral lipid catabolic process	4/255	73/29151	0.003880866	0.04944974	0.04184718	APOA1/APOA2/APOC3/APOB	4
GO:0046464	acylglycerol catabolic process	4/255	73/29151	0.003880866	0.04944974	0.04184718	APOA1/APOA2/APOC3/APOB	4
GO:0001818	negative regulation of cytokine production	11/255	487/29151	0.003971284	0.04944974	0.04184718	APOA1/APOA2/FN1/ORM1/LTF/ANXA1/ARG1/APOD/HSP90AB1/LGALS7/TMSB4X	11
GO:0034382	chylomicron remnant clearance	2/255	11/29151	0.003979478	0.04944974	0.04184718	APOE/APOC3	2
GO:0034447	very-low-density lipoprotein particle clearance	2/255	11/29151	0.003979478	0.04944974	0.04184718	APOE/APOC3	2
GO:0045917	positive regulation of complement activation	2/255	11/29151	0.003979478	0.04944974	0.04184718	C3/PHB1	2
GO:0051029	rRNA transport	2/255	11/29151	0.003979478	0.04944974	0.04184718	NPM1/TST	2
GO:0051838	cytolysis by host of symbiont cells	2/255	11/29151	0.003979478	0.04944974	0.04184718	APOL1/HRG	2
GO:0070268	cornification	2/255	11/29151	0.003979478	0.04944974	0.04184718	CASP14/CDSN	2
GO:0071830	triglyceride-rich lipoprotein particle clearance	2/255	11/29151	0.003979478	0.04944974	0.04184718	APOE/APOC3	2
GO:0086073	bundle of His cell-Purkinje myocyte adhesion involved in cell communication	2/255	11/29151	0.003979478	0.04944974	0.04184718	JUP/DSP	2
GO:0030048	actin filament-based movement	8/255	288/29151	0.004024713	0.04944974	0.04184718	ACTN4/MYH6/JUP/DSP/FLNA/MYH9/MYH10/ACTC1	8
GO:0019318	hexose metabolic process	10/255	419/29151	0.00404608	0.04944974	0.04184718	PGK1/ALDOA/GAPDH/APOD/ENO1/PKM/MDH2/TPI1/FABP5/FAM3C	10
GO:0045333	cellular respiration	10/255	419/29151	0.00404608	0.04944974	0.04184718	IDH1/COX2/CAT/ATP5F1B/DLD/COX4I1/ATP5F1A/ETFB/MDH2/PLEC	10
GO:0034620	cellular response to unfolded protein	6/255	171/29151	0.004047633	0.04944974	0.04184718	HSPA1A/HSPD1/HSPA5/HSPA8/HSPA6/HSPA9	6
GO:0071711	basement membrane organization	4/255	74/29151	0.004074905	0.04944974	0.04184718	LAMB1/LAMC1/NID1/LAMB2	4
GO:0090670	RNA localization to Cajal body	3/255	37/29151	0.0041267	0.04944974	0.04184718	CCT5/CCT8/CCT2	3
GO:0090671	telomerase RNA localization to Cajal body	3/255	37/29151	0.0041267	0.04944974	0.04184718	CCT5/CCT8/CCT2	3
GO:0090672	telomerase RNA localization	3/255	37/29151	0.0041267	0.04944974	0.04184718	CCT5/CCT8/CCT2	3

GO:0090685	RNA localization to nucleus	3/255	37/29151	0.0041267	0.04944974	0.04184718	CCT5/CCT8/CCT2	3
GO:2001028	positive regulation of endothelial cell chemotaxis	3/255	37/29151	0.0041267	0.04944974	0.04184718	HSPB1/FGF2/TMSB4X	3
GO:0051091	positive regulation of DNA-binding transcription factor activity	11/255	490/29151	0.004156541	0.04963733	0.042005929	AGT/LTF/CAT/S100A8/S100A9/NPM1/HSPA1A/CLU/JUP/PSMA6/PPIA	11
GO:0072593	reactive oxygen species metabolic process	10/255	421/29151	0.004182335	0.049775481	0.042122841	COX2/HP/AGT/CAT/PRDX5/PRDX2/HBB/HBA1/PRDX1/PRDX4	10

Table S4. Percentage of EV Population

Group	EV population	Count	Percentage (/Total EV)
Healthy	$CD63^{+}APLP1^{-}$ EV	24.0±4.73	63.72 %
Healthy	$CD63^{+}APLP1^{+}$ EV	13.67±6.33	36.28 %
GBM	$CD63^{+}APLP1^{-}$ EV	260.67±51.43	31.18 %
GBM	$CD63^{+}APLP1^{+}$ EV	575.33±63.94	68.82 %

Table S5. List of Primers Used in the Study

**Human**	**Forward Primers**	**Reverse Primers**
APLP1	GCG TAG GAT GCG CCA GAT TA	GGT CGT TGA TAA GGG CGA TGA
ENO2	GTG CAC AGG CCA GAT CAA GA	ACA GCA CAC TGG GAT TAC GG
Tuj1	GGG CCT TTG GAC ATC TCT TC	CCT CCG TGT AGT GAC CCT TG
MAP2	CCA ATG GAT TCC CAT ACA GG	CTG CTA CAG CCT CAG CAG TG
GAPDH	CGA GAT CCC TCC AAA ATC AA	TGT GGT CAT GAG TCC TTC CA
**Mouse**		
APLP1	GCC ACT GTC ATT GCT GCT TC	GGG TTA GAC GCC CAC ATA GTC
L1CAM	AAA GGT GCA AGG GTG ACA TTC	TCC CCA CGT TCC TGT AGG T
GAPDH	TCA CTG CCA CCC AGA ACA	GAC GGA CAC ATT GGG GGT AC
